# Supplementary material for: Characterisation of a type II functionally-deficient variant of alpha-1-antitrypsin discovered in the general population
Source: PLoS One. 2019 Jan 11;14(1):e0206955. doi: 10.1371/journal.pone.0206955 (PMC6329500; doi:10.1371/journal.pone.0206955)
Supplement: S1 Table — (DOCX) [file pone.0206955.s002.docx]

**Supplementary Table S1**

**Table S1:** Reagents list.

| **Reagent** | **Provider** | **Code** | **Country** |
| --- | --- | --- | --- |
| pcDNA3.1/Zeo (+) | Thermo Fisher | #V86020 | Monza, Italy |
| pcDNA3.1(+) | Thermo Fisher | # V79520 | Monza, Italy |
| Optimem | Thermo Fisher | # 11058021 | Monza, Italy |
| QuikChange II Site-Directed Mutagenesis Kit | Agilent Technologies | #200523 | Santa Clara, USA |
| Polyethyleneimine “Max” 25kDa | Polysciences Inc | # 23966-1 | Heidelberg, Germany |
| ECL Clarity | Biorad | #1705060 | Segrate, Italy |
| PVDF | Biorad | #1620260 | Segrate, Italy |
| Hyperfilm ECL | GE Healhcare | #28906837 | Milan, Italy |
| DMEM | Sigma | # D5796 | Milan, Italy |
| FBS | Sigma | # F0392 | Milan, Italy |
| PBS | Sigma | # D5773 | Milan, Italy |
| Protease inhibitor cocktail | Sigma | # P8340 | Milan, Italy |
| PEG8000 | Sigma | #1546605 | Milan, Italy |
| HNE | Sigma | #E8140 | Milan, Italy |
| PPE | Sigma | #45124 | Milan, Italy |
| CHT | Sigma | #C4129 | Milan, Italy |
| KLKB1 | RayBiotech | #MD-14-0043P | Cambridge, UK |
| Rabbit anti-A1AT pAb | DAKO | #A0012 | Milan, Italy |
| Sheep anti-A1AT - HRP conjugated pAb | Abcam | #Ab0768 | Cambridge, UK |
