# Supplementary material for: Characterisation of a type II functionally-deficient variant of alpha-1-antitrypsin discovered in the general population
Source: PLoS One. 2019 Jan 11;14(1):e0206955. doi: 10.1371/journal.pone.0206955 (PMC6329500; doi:10.1371/journal.pone.0206955)
Supplement: S1 File — (DOCX) [file pone.0206955.s003.docx]

**Supplementary File 1**

List of the *SERPINA1* orthologues used for the MSA (multiple sequence alignment) plotted below:

**> 1qlp | Input_pdb_SEQRES_A SERPINA1 Human**

> A0A093G1U7 | A0A093G1U7_TYTAL_1_384 | Alpha-1-antiproteinase 2 (Fragment) OS=Tyto alba GN=N341_12020 PE=3 SV=1

> B5BV12 | B5BV12_HORSE_26_421 | Alpha-1-antitrypsin OS=Equus caballus GN=Spi2-13 PE=2 SV=1

> A0A093H5C6 | A0A093H5C6_PICPB_1_371 | Alpha-1-antitrypsin-like GS55-MS (Fragment) OS=Picoides pubescens GN=N307_13630 PE=3 SV=1

> K7FUC4 | K7FUC4_PELSI_37_423 | Uncharacterized protein OS=Pelodiscus sinensis PE=3 SV=1

> A0A091VAK7 | A0A091VAK7_NIPNI_2_372 | Alpha-1-antiproteinase 2 (Fragment) OS=Nipponia nippon GN=Y956_05067 PE=3 SV=1

> U3JIK3 | U3JIK3_FICAL_33_418 | Uncharacterized protein OS=Ficedula albicollis GN=LOC101809980 PE=3 SV=1

> K7FR61 | K7FR61_PELSI_25_416 | Uncharacterized protein OS=Pelodiscus sinensis PE=3 SV=1

> A0A091U8N8 | A0A091U8N8_PHORB_1_384 | Uncharacterized protein (Fragment) OS=Phoenicopterus ruber ruber GN=N337_13209 PE=3 SV=1

> K7FSH8 | K7FSH8_PELSI_22_430 | Uncharacterized protein OS=Pelodiscus sinensis PE=3 SV=1

> A0A093GVB9 | A0A093GVB9_PICPB_1_382 | Alpha-1-antiproteinase 2 (Fragment) OS=Picoides pubescens GN=N307_13631 PE=3 SV=1

> G3TDW3 | G3TDW3_LOXAF_26_418 | Uncharacterized protein OS=Loxodonta africana GN=LOC100664453 PE=4 SV=1

> A0A093GX57 | A0A093GX57_STRCA_1_384 | Alpha-1-antiproteinase 2 (Fragment) OS=Struthio camelus australis GN=N308_06911 PE=3 SV=1

> A0A091K7I5 | A0A091K7I5_COLST_2_372 | Alpha-1-antiproteinase 2 (Fragment) OS=Colius striatus GN=N325_03298 PE=3 SV=1

> U3FMP8 | U3FMP8_CALJA_26_418 | Alpha-1-antitrypsin OS=Callithrix jacchus GN=SERPINA1 PE=2 SV=1

> A0A091NIR3 | A0A091NIR3_9PASS_1_384 | Uncharacterized protein (Fragment) OS=Acanthisitta chloris GN=N310_04938 PE=3 SV=1

> A0A091EGC4 | A0A091EGC4_CORBR_2_372 | Alpha-1-antitrypsin-like GS55-MS (Fragment) OS=Corvus brachyrhynchos GN=N302_00099 PE=3 SV=1

> H0ZQY2 | H0ZQY2_TAEGU_1_372 | Uncharacterized protein OS=Taeniopygia guttata GN=SERPINA12-1 PE=3 SV=1

> A0A091LFJ1 | A0A091LFJ1_9GRUI_1_384 | Alpha-1-antiproteinase (Fragment) OS=Chlamydotis macqueenii GN=N324_04577 PE=3 SV=1

> P17475 | A1AT_RAT_24_410 | Alpha-1-antiproteinase OS=Rattus norvegicus GN=Serpina1 PE=1 SV=2

> W5PZS7 | W5PZS7_SHEEP_28_415 | Uncharacterized protein OS=Ovis aries GN=SERPINA1 PE=3 SV=1

> U3IXC3 | U3IXC3_ANAPL_31_424 | Uncharacterized protein OS=Anas platyrhynchos GN=SERPINA1 PE=3 SV=1

> A0A091FX98 | A0A091FX98_9AVES_2_372 | Alpha-1-antiproteinase 2 (Fragment) OS=Cuculus canorus GN=N303_15534 PE=3 SV=1

> A0A091XH23 | A0A091XH23_OPIHO_1_371 | Uncharacterized protein (Fragment) OS=Opisthocomus hoazin GN=N306_04596 PE=3 SV=1

> Q76HN9 | Q76HN9_TAMSI_24_413 | Alpha1-antitrypsin-like protein OS=Tamias sibiricus GN=CM55-MS PE=3 SV=1

> M7B5J6 | M7B5J6_CHEMY_25_431 | Alpha-1-antiproteinase OS=Chelonia mydas GN=UY3_10486 PE=3 SV=1

> A0A091RAD2 | A0A091RAD2_9GRUI_2_365 | Alpha-1-antiproteinase 2 (Fragment) OS=Mesitornis unicolor GN=N332_06634 PE=3 SV=1

> P97277 | A1AT_MESAU_24_412 | Alpha-1-antitrypsin OS=Mesocricetus auratus PE=2 SV=1

> A0A091RXN3 | A0A091RXN3_NESNO_1_382 | Alpha-1-antiproteinase 2 (Fragment) OS=Nestor notabilis GN=N333_10026 PE=3 SV=1

> G1PM73 | G1PM73_MYOLU_29_418 | Uncharacterized protein (Fragment) OS=Myotis lucifugus GN=SERPINA1 PE=3 SV=1

> H0WJ03 | H0WJ03_OTOGA_26_416 | Uncharacterized protein OS=Otolemur garnettii GN=SERPINA1 PE=3 SV=1

> O54761 | ALMS_ICTTR_24_411 | Alpha-1-antitrypsin-like protein GS55-MS OS=Ictidomys tridecemlineatus PE=2 SV=1

> R0LEI7 | R0LEI7_ANAPL_30_418 | Alpha-1-antitrypsin (Fragment) OS=Anas platyrhynchos GN=LOC101790996 PE=3 SV=1

> A0A087R544 | A0A087R544_APTFO_1_384 | Alpha-1-antiproteinase 2 (Fragment) OS=Aptenodytes forsteri GN=AS27_04572 PE=3 SV=1

> Q76HP0 | Q76HP0_TAMSI_24_413 | Alpha1-antitrypsin-like protein OS=Tamias sibiricus GN=CM55-MM PE=3 SV=1

> L5LNJ6 | L5LNJ6_MYODS_26_417 | Alpha-1-antitrypsin OS=Myotis davidii GN=MDA_GLEAN10008164 PE=3 SV=1

> A0A093R765 | A0A093R765_PHACA_1_384 | Uncharacterized protein (Fragment) OS=Phalacrocorax carbo GN=N336_03476 PE=3 SV=1

> G1NKI3 | G1NKI3_MELGA_25_422 | Uncharacterized protein OS=Meleagris gallopavo GN=LOC100542070 PE=3 SV=2

> A0A093JEM7 | A0A093JEM7_EURHL_1_382 | Uncharacterized protein (Fragment) OS=Eurypyga helias GN=N326_03194 PE=3 SV=1

> A0A093IZH1 | A0A093IZH1_EURHL_2_372 | Alpha-1-antiproteinase 2 (Fragment) OS=Eurypyga helias GN=N326_12390 PE=3 SV=1

> A0A091GSL7 | A0A091GSL7_BUCRH_1_382 | Uncharacterized protein (Fragment) OS=Buceros rhinoceros silvestris GN=N320_09566 PE=3 SV=1

> A0A099Z110 | A0A099Z110_TINGU_1_371 | Uncharacterized protein (Fragment) OS=Tinamus guttatus GN=N309_12029 PE=3 SV=1

> A0A091T9D5 | A0A091T9D5_PHALP_1_384 | Alpha-1-antiproteinase (Fragment) OS=Phaethon lepturus GN=N335_06937 PE=3 SV=1

> A0A091PDX7 | A0A091PDX7_HALAL_1_384 | Alpha-1-antiproteinase 2 (Fragment) OS=Haliaeetus albicilla GN=N329_04313 PE=3 SV=1

> A0A093CUY6 | A0A093CUY6_TAUER_1_386 | Uncharacterized protein (Fragment) OS=Tauraco erythrolophus GN=N340_13303 PE=3 SV=1

> M3WCX1 | M3WCX1_FELCA_28_419 | Uncharacterized protein OS=Felis catus GN=SERPINA1 PE=3 SV=1

> A0A091QM83 | A0A091QM83_MERNU_1_384 | Alpha-1-antiproteinase (Fragment) OS=Merops nubicus GN=N331_08113 PE=3 SV=1

> Q07298 | Q07298_RABIT_24_412 | Alpha-1-antiproteinase S-1 OS=Oryctolagus cuniculus GN=LOC100008980 PE=2 SV=1

> A0A0A0ABV8 | A0A0A0ABV8_CHAVO_1_384 | Uncharacterized protein (Fragment) OS=Charadrius vociferus GN=N301_06973 PE=3 SV=1

> A0A093PVA9 | A0A093PVA9_9PASS_1_371 | Uncharacterized protein (Fragment) OS=Manacus vitellinus GN=N305_01292 PE=3 SV=1

> A0A091JCG9 | A0A091JCG9_9AVES_1_384 | Uncharacterized protein (Fragment) OS=Egretta garzetta GN=Z169_04208 PE=3 SV=1

> R0JRB7 | R0JRB7_ANAPL_32_430 | Alpha-1-antiproteinase 2 (Fragment) OS=Anas platyrhynchos GN=Anapl_03306 PE=3 SV=1

> A0A093PGJ8 | A0A093PGJ8_9PASS_1_385 | Alpha-1-antiproteinase 2 (Fragment) OS=Manacus vitellinus GN=N305_01293 PE=3 SV=1

> O54760 | ALSI_TAMSI_24_413 | Alpha-1-antitrypsin-like protein CM55-SI OS=Tamias sibiricus PE=2 SV=1

> A0A091V8N3 | A0A091V8N3_NIPNI_1_384 | Alpha-1-antiproteinase 2 (Fragment) OS=Nipponia nippon GN=Y956_05068 PE=3 SV=1

> A0A091PB47 | A0A091PB47_9PASS_2_372 | Alpha-1-antiproteinase (Fragment) OS=Acanthisitta chloris GN=N310_04939 PE=3 SV=1

> G1NKH5 | G1NKH5_MELGA_31_418 | Uncharacterized protein (Fragment) OS=Meleagris gallopavo GN=LOC100544555 PE=3 SV=1

> A0A093H779 | A0A093H779_STRCA_2_372 | Alpha-1-antiproteinase 2 (Fragment) OS=Struthio camelus australis GN=N308_06910 PE=3 SV=1

> L5JP19 | L5JP19_PTEAL_26_413 | Alpha-1-antitrypsin OS=Pteropus alecto GN=PAL_GLEAN10020808 PE=3 SV=1

> P01010 | A1AT_PAPAN_17_409 | Alpha-1-antitrypsin (Fragment) OS=Papio anubis GN=SERPINA1 PE=2 SV=1

> A0A093KV79 | A0A093KV79_FULGA_1_371 | Uncharacterized protein (Fragment) OS=Fulmarus glacialis GN=N327_00956 PE=3 SV=1

> A0A091KRT6 | A0A091KRT6_COLST_2_372 | Alpha-1-antitrypsin-like GS55-MS (Fragment) OS=Colius striatus GN=N325_05428 PE=3 SV=1

> A0A091WBP3 | A0A091WBP3_OPIHO_1_384 | Uncharacterized protein (Fragment) OS=Opisthocomus hoazin GN=N306_04595 PE=3 SV=1

> A0A093F995 | A0A093F995_GAVST_1_384 | Alpha-1-antiproteinase (Fragment) OS=Gavia stellata GN=N328_06806 PE=3 SV=1

> S7ME51 | S7ME51_MYOBR_24_418 | Kallistatin OS=Myotis brandtii GN=D623_10004138 PE=3 SV=1

> P50447 | A1AT_PIG_28_421 | Alpha-1-antitrypsin OS=Sus scrofa GN=SERPINA1 PE=2 SV=1

> M3XVV7 | M3XVV7_MUSPF_55_427 | Uncharacterized protein OS=Mustela putorius furo GN=SERPINA1 PE=3 SV=1

> A0A091KQF0 | A0A091KQF0_9GRUI_2_372 | Alpha-1-antiproteinase 2 (Fragment) OS=Chlamydotis macqueenii GN=N324_04579 PE=3 SV=1

> G1KWY8 | G1KWY8_ANOCA_22_428 | Uncharacterized protein OS=Anolis carolinensis GN=LOC100560217 PE=3 SV=2

> A0A091G0Q5 | A0A091G0Q5_9AVES_1_384 | Alpha-1-antitrypsin (Fragment) OS=Cuculus canorus GN=N303_15535 PE=3 SV=1

> A0A091LM39 | A0A091LM39_CATAU_1_384 | Alpha-1-antitrypsin (Fragment) OS=Cathartes aura GN=N323_12911 PE=3 SV=1

> E1C206 | E1C206_CHICK_40_435 | Uncharacterized protein OS=Gallus gallus GN=SERPINA5 PE=3 SV=2

> G3I296 | G3I296_CRIGR_24_411 | Alpha-1-antitrypsin OS=Cricetulus griseus GN=I79_017525 PE=3 SV=1

> Q8JIA6 | Q8JIA6_SPHPU_38_426 | Alpha-1-antitrypsin (Fragment) OS=Sphenodon punctatus PE=2 SV=1

> O54763 | A1AT_CALCN_24_412 | Alpha-1-antiproteinase OS=Callosciurus caniceps PE=2 SV=1

> A0A091QJI6 | A0A091QJI6_MERNU_2_367 | Alpha-1-antiproteinase 2 (Fragment) OS=Merops nubicus GN=N331_08112 PE=3 SV=1

> A0A091NC43 | A0A091NC43_APAVI_1_385 | Alpha-1-antiproteinase 2 (Fragment) OS=Apaloderma vittatum GN=N311_02610 PE=3 SV=1

> A0A091PLB4 | A0A091PLB4_APAVI_2_372 | Alpha-1-antiproteinase 2 (Fragment) OS=Apaloderma vittatum GN=N311_05384 PE=3 SV=1

> O62663 | O62663_RABIT_67_455 | ATS-22 OS=Oryctolagus cuniculus PE=2 SV=1

> A0A087VMC1 | A0A087VMC1_BALRE_1_385 | Alpha-1-antiproteinase (Fragment) OS=Balearica regulorum gibbericeps GN=N312_01149 PE=3 SV=1

> F1PCE5 | F1PCE5_CANFA_28_419 | Uncharacterized protein OS=Canis familiaris GN=SERPINA1 PE=3 SV=2

> A0A091LUF5 | A0A091LUF5_CARIC_1_384 | Alpha-1-antiproteinase (Fragment) OS=Cariama cristata GN=N322_05679 PE=3 SV=1

> A0A093DUW7 | A0A093DUW7_9AVES_1_384 | Alpha-1-antitrypsin (Fragment) OS=Pterocles gutturalis GN=N339_11665 PE=3 SV=1

> K7FUQ7 | K7FUQ7_PELSI_3_378 | Uncharacterized protein OS=Pelodiscus sinensis PE=3 SV=1

> B5BV05 | B5BV05_HORSE_26_421 | Alpha-1-antitrypsin OS=Equus caballus GN=Spi2-6 PE=2 SV=1

> M7B3E7 | M7B3E7_CHEMY_25_431 | Alpha-1-antiproteinase 2 OS=Chelonia mydas GN=UY3_10483 PE=3 SV=1

> A0A091V9J5 | A0A091V9J5_NIPNI_1_374 | Alpha-1-antiproteinase 2 (Fragment) OS=Nipponia nippon GN=Y956_05070 PE=3 SV=1

> F7CYP1 | F7CYP1_HORSE_26_423 | Uncharacterized protein OS=Equus caballus GN=LOC100065191 PE=3 SV=1

> M7B3F1 | M7B3F1_CHEMY_55_425 | Alpha-1-antiproteinase 2 OS=Chelonia mydas GN=UY3_10488 PE=3 SV=1

> P23035 | A1AF_RABIT_24_412 | Alpha-1-antiproteinase F OS=Oryctolagus cuniculus PE=1 SV=1

> L8YAZ3 | L8YAZ3_TUPCH_26_418 | Alpha-1-antitrypsin OS=Tupaia chinensis GN=TREES_T100005837 PE=3 SV=1

> D2HEM3 | D2HEM3_AILME_26_421 | Putative uncharacterized protein (Fragment) OS=Ailuropoda melanoleuca GN=PANDA_009257 PE=3 SV=1

> R0LHU9 | R0LHU9_ANAPL_26_414 | Alpha-1-antiproteinase (Fragment) OS=Anas platyrhynchos GN=Anapl_03302 PE=3 SV=1

> Q76HP1 | Q76HP1_TAMSI_24_413 | Alpha1-antitrypsin-like protein OS=Tamias sibiricus GN=CM55-ML PE=3 SV=1

> A0A093CLJ8 | A0A093CLJ8_TAUER_1_371 | Uncharacterized protein (Fragment) OS=Tauraco erythrolophus GN=N340_13304 PE=3 SV=1

> A0A093GS00 | A0A093GS00_PICPB_1_370 | Alpha-1-antiproteinase 2 (Fragment) OS=Picoides pubescens GN=N307_08460 PE=3 SV=1

> A0A093QJ65 | A0A093QJ65_PHACA_2_371 | Alpha-1-antiproteinase 2 (Fragment) OS=Phalacrocorax carbo GN=N336_04429 PE=3 SV=1

> G1S644 | G1S644_NOMLE_109_501 | Uncharacterized protein OS=Nomascus leucogenys GN=SERPINA1 PE=3 SV=2

> G1NKH1 | G1NKH1_MELGA_1_383 | Uncharacterized protein (Fragment) OS=Meleagris gallopavo GN=LOC100542224 PE=3 SV=1

> P34955 | A1AT_BOVIN_28_415 | Alpha-1-antiproteinase OS=Bos taurus GN=SERPINA1 PE=1 SV=1

> A0A094KEC1 | A0A094KEC1_ANTCR_1_384 | Alpha-1-antitrypsin (Fragment) OS=Antrostomus carolinensis GN=N321_03316 PE=3 SV=1

> A0A087VMC2 | A0A087VMC2_BALRE_1_370 | Uncharacterized protein (Fragment) OS=Balearica regulorum gibbericeps GN=N312_01150 PE=3 SV=1

> A0A099YYG0 | A0A099YYG0_TINGU_1_384 | Alpha-1-antitrypsin (Fragment) OS=Tinamus guttatus GN=N309_12030 PE=3 SV=1

> A0A093PHW0 | A0A093PHW0_9PASS_2_372 | Alpha-1-antiproteinase 2 (Fragment) OS=Manacus vitellinus GN=N305_01291 PE=3 SV=1

> K7FUR6 | K7FUR6_PELSI_30_421 | Uncharacterized protein OS=Pelodiscus sinensis PE=3 SV=1

> A0A0A0A7A0 | A0A0A0A7A0_CHAVO_1_371 | Alpha-1-antiproteinase 2 (Fragment) OS=Charadrius vociferus GN=N301_06972 PE=3 SV=1

> A0A091CQE0 | A0A091CQE0_FUKDA_24_413 | Alpha-1-antiproteinase S OS=Fukomys damarensis GN=H920_17268 PE=3 SV=1

> A0A093FVA3 | A0A093FVA3_TYTAL_2_372 | Alpha-1-antiproteinase 2 (Fragment) OS=Tyto alba GN=N341_12019 PE=3 SV=1

> A0A093P9B6 | A0A093P9B6_PYGAD_2_372 | Alpha-1-antiproteinase 2 (Fragment) OS=Pygoscelis adeliae GN=AS28_10539 PE=3 SV=1

> A0A091GLC8 | A0A091GLC8_9AVES_2_372 | Alpha-1-antiproteinase 2 (Fragment) OS=Cuculus canorus GN=N303_15536 PE=3 SV=1

> Q64118 | A1AT_MERUN_24_406 | Alpha-1-antitrypsin OS=Meriones unguiculatus PE=1 SV=1

> F6SWD3 | F6SWD3_ORNAN_1_379 | Uncharacterized protein OS=Ornithorhynchus anatinus GN=LOC100086374 PE=3 SV=1

> A0A091T7N1 | A0A091T7N1_PHALP_2_372 | Alpha-1-antiproteinase 2 (Fragment) OS=Phaethon lepturus GN=N335_06938 PE=3 SV=1

> A0A091NZH9 | A0A091NZH9_9PASS_6_368 | Alpha-1-antiproteinase 2 (Fragment) OS=Acanthisitta chloris GN=N310_08234 PE=3 SV=1

> A0A091PCC0 | A0A091PCC0_HALAL_1_361 | Uncharacterized protein (Fragment) OS=Haliaeetus albicilla GN=N329_04314 PE=3 SV=1

> A0A091MK58 | A0A091MK58_CARIC_2_372 | Alpha-1-antiproteinase 2 (Fragment) OS=Cariama cristata GN=N322_05680 PE=3 SV=1

> A0A091F2Q1 | A0A091F2Q1_CORBR_1_371 | Uncharacterized protein (Fragment) OS=Corvus brachyrhynchos GN=N302_00098 PE=3 SV=1

> A0A093RZ50 | A0A093RZ50_9PASS_2_372 | Alpha-1-antiproteinase 2 (Fragment) OS=Manacus vitellinus GN=N305_01294 PE=3 SV=1

> K7A8Q9 | K7A8Q9_PANTR_24_422 | Serpin peptidase inhibitor, clade A (Alpha-1 antiproteinase, antitrypsin), member 3 OS=Pan troglodytes GN=SERPINA3 PE=2 SV=1

> A0A093IEQ9 | A0A093IEQ9_EURHL_2_372 | Alpha-1-antiproteinase 2 (Fragment) OS=Eurypyga helias GN=N326_11967 PE=3 SV=1

> A0A091K0Z5 | A0A091K0Z5_9AVES_2_372 | Alpha-1-antiproteinase 2 (Fragment) OS=Egretta garzetta GN=Z169_04209 PE=3 SV=1

> A0A091HV30 | A0A091HV30_BUCRH_2_372 | Alpha-1-antiproteinase 2 (Fragment) OS=Buceros rhinoceros silvestris GN=N320_01151 PE=3 SV=1

> Q66KX6 | Q66KX6_XENLA_43_431 | MGC85345 protein OS=Xenopus laevis GN=serpina3k PE=2 SV=1

> A0A091LT78 | A0A091LT78_CARIC_2_369 | Alpha-1-antitrypsin-like GS55-MS (Fragment) OS=Cariama cristata GN=N322_03284 PE=3 SV=1

> A0A091SNX6 | A0A091SNX6_9AVES_1_384 | Uncharacterized protein (Fragment) OS=Pelecanus crispus GN=N334_06797 PE=3 SV=1

> A0A087R545 | A0A087R545_APTFO_1_361 | Uncharacterized protein (Fragment) OS=Aptenodytes forsteri GN=AS27_04573 PE=3 SV=1

> A0A093H648 | A0A093H648_STRCA_1_372 | Alpha-1-antiproteinase 2 (Fragment) OS=Struthio camelus australis GN=N308_06913 PE=3 SV=1

> G1NKH9 | G1NKH9_MELGA_30_422 | Uncharacterized protein OS=Meleagris gallopavo GN=LOC100544713 PE=3 SV=1

> A0A091KNU4 | A0A091KNU4_9GRUI_1_370 | Uncharacterized protein (Fragment) OS=Chlamydotis macqueenii GN=N324_04578 PE=3 SV=1

> K9IXP7 | K9IXP7_DESRO_26_418 | Putative alpha-1-antichymotrypsin OS=Desmodus rotundus PE=2 SV=1

> A0A091KYD1 | A0A091KYD1_COLST_1_382 | Alpha-1-antitrypsin (Fragment) OS=Colius striatus GN=N325_04385 PE=3 SV=1

> A0A091QU84 | A0A091QU84_MERNU_2_372 | Alpha-1-antiproteinase 2 (Fragment) OS=Merops nubicus GN=N331_02960 PE=3 SV=1

> A2I7N2 | SPA36_BOVIN_24_413 | Serpin A3-6 OS=Bos taurus GN=SERPINA3-6 PE=3 SV=1

> P07758 | A1AT1_MOUSE_24_413 | Alpha-1-antitrypsin 1-1 OS=Mus musculus GN=Serpina1a PE=1 SV=4

> A0A091T7I8 | A0A091T7I8_PHALP_1_361 | Uncharacterized protein (Fragment) OS=Phaethon lepturus GN=N335_06936 PE=3 SV=1

> A0A091SWC2 | A0A091SWC2_NESNO_2_372 | Alpha-1-antiproteinase 2 (Fragment) OS=Nestor notabilis GN=N333_10025 PE=3 SV=1

> F7CZD2 | F7CZD2_ORNAN_29_431 | Uncharacterized protein OS=Ornithorhynchus anatinus GN=SERPINA4 PE=3 SV=1

> A0A093C116 | A0A093C116_9AVES_1_371 | Uncharacterized protein (Fragment) OS=Pterocles gutturalis GN=N339_11666 PE=3 SV=1

> Q00898 | A1AT5_MOUSE_24_413 | Alpha-1-antitrypsin 1-5 OS=Mus musculus GN=Serpina1e PE=1 SV=1

> A0A093FFU5 | A0A093FFU5_GAVST_1_364 | Uncharacterized protein (Fragment) OS=Gavia stellata GN=N328_06805 PE=3 SV=1

> A0A091QVX2 | A0A091QVX2_9GRUI_1_379 | Uncharacterized protein (Fragment) OS=Mesitornis unicolor GN=N332_04248 PE=3 SV=1

> F1SCC6 | F1SCC6_PIG_27_422 | Uncharacterized protein OS=Sus scrofa GN=LOC100153899 PE=1 SV=2

> A0A093IN74 | A0A093IN74_EURHL_1_371 | Uncharacterized protein (Fragment) OS=Eurypyga helias GN=N326_12391 PE=3 SV=1

> A0A0D9REC0 | A0A0D9REC0_CHLSB_49_447 | Uncharacterized protein OS=Chlorocebus sabaeus PE=3 SV=1

> Q63969 | A1AT_MUSSA_24_413 | Alpha-1-antiproteinase OS=Mus saxicola GN=Serpina1 PE=2 SV=1

> G5B496 | G5B496_HETGA_31_415 | Alpha-1-antiproteinase S OS=Heterocephalus glaber GN=GW7_16189 PE=3 SV=1

> A0A091U6W4 | A0A091U6W4_PHORB_2_371 | Alpha-1-antiproteinase 2 (Fragment) OS=Phoenicopterus ruber ruber GN=N337_13210 PE=3 SV=1

> A0A091KQX5 | A0A091KQX5_9GRUI_2_372 | Alpha-1-antiproteinase 2 (Fragment) OS=Chlamydotis macqueenii GN=N324_04576 PE=3 SV=1

> Q5R536 | AACT_PONAB_24_422 | Alpha-1-antichymotrypsin OS=Pongo abelii GN=SERPINA3 PE=2 SV=1

> M3XVL7 | M3XVL7_MUSPF_24_426 | Uncharacterized protein OS=Mustela putorius furo GN=SERPINA4 PE=3 SV=1

CLUSTAL Omega (1.2.4) MSA of the SERPINA1 orthologues.

A0A093GX57 ----------------------DRRQAEGENLAHLKIAPSNADFAFRFYKQVAAEAG-DK 37

A0A099YYG0 ----------------------DHSQVEGENLPHLKIAPGNAEFAFRFYKQVAAEGG-DK 37

G1NKH9 ---HKNEQPH---------SLGDHSHVESENSAHMKITPRNAEFAFRFYKQVTEEGG-NK 47

U3IXC3 -------QED---------QDTAHSPAEGENLAHLKIAPSNADFAFRFYKQVTEEEG-NK 43

A0A091KYD1 ----------------------DYSQAEGENLAHVKIAPSNADFAFKFYKQIRDEVGNNK 38

A0A091QVX2 ---------------------------EDENLAYIKIVHSNIDFAFKFYKQIREEAG-NK 32

A0A091NC43 ----------------------DHSQDEGENLAHIKIASSNTDFAFRLYKQVREEAG-NE 37

A0A091GSL7 ------------------------SQAEGKNLAHVKIASSNADFAFKFYKQVREENS-NK 35

A0A093PGJ8 ----------------------DHSQAEDENLPHVKIAPSNADFAFRFYKQVREEAG-NK 37

A0A091NIR3 ----------------------DHSQAEGEHLPHTKIAPSNSNFAFRFYKQVREEAG-NK 37

A0A091G0Q5 ----------------------DHSQTEGEKLGLVKIVPSNTDFAFQFYKQVREEEG-NK 37

A0A093GVB9 ------------------------SNDESENLAHVKIIDNNADFAFRFYRQVREEAG-NK 35

A0A093CUY6 ----------------------DHSHTEGENSAHVKIAPSNVDFAFRFYKQVREEAG-NK 37

A0A091QM83 ----------------------DHSQAEGENLAHVKIVSSNSDFAFRFYKQVREEGD-NK 37

A0A091PDX7 ----------------------DHSQAEDENLAHVKIASSNTDFAFRFYKQVRKEAS-NK 37

A0A091WBP3 ----------------------DHSQAEDENLAHDKIALSNADFAFRFYKQVREEAG-NK 37

A0A091SNX6 ----------------------DHTHAEGENLAHVKIAPSNAEFAFRFYKQVREEAG-NK 37

A0A094KEC1 ----------------------DHSQAEGENLAHVKIAPSNADFAFRFYKQIREEDS-NK 37

A0A0A0ABV8 ----------------------DHSQSEDENLPHVKIAPSNAEFAFRFYQQIREEVG-NK 37

A0A093JEM7 ------------------------DHSQAENSAHVKIAPGNADFAFRFYKQVREEVG-NK 35

A0A093G1U7 ----------------------DSYQSGGENLAHVKITPSNADFAFRFYKQVRDEAS-NK 37

A0A091JCG9 ----------------------DHSQSEGENLAHVKIASSNADFAFKFYKQLREEVG-NK 37

A0A091U8N8 ----------------------DHSQAEGENSAHVKIAPSNTDFAFRLYKQIREEVG-NK 37

A0A091RXN3 ------------------------SQAEGENLAHVKIAPNNADFAFRFYKQAREEAG-NK 35

A0A093R765 ----------------------DHSQPDGENLAHIKMAPSNYDFAFRFYKQVREEVG-NK 37

A0A087VMC1 ----------------------DHSQVEDENLALVKIAPSNAEFAFRFYKQVREEAG-NK 37

A0A093F995 ----------------------DHSQAEGENLAHVKIAPSNIDFAFRFYKQVREEAG-NK 37

A0A093DUW7 ----------------------DDFQDEGENLAHVKIAPSNADFAFRFYKQIREKAD-NK 37

A0A091T9D5 ----------------------DHSQPEGENLAHVKIAPSNADFAFRFYKQVREEAG-NK 37

A0A091LUF5 ----------------------DHSQAEGENSAHVKIAPSNADFAFSFYKQVREEAG-NK 37

A0A091V8N3 ----------------------DHFQAEGENLAHVKIAASNADFAFRFYKQVREEVG-NK 37

A0A091LFJ1 ----------------------DHSPAEGENLAHVKIASSNADFGFRFYKQVREEVG-SK 37

A0A087R544 ----------------------DHSQAEGENLAHVKIAPSNADFAFRFYKQVREEAG-NK 37

A0A091LM39 ----------------------DHSQAEGENLAHVKIAPGNADFAFRFYKQVREQAG-NK 37

Q66KX6 ---------------SDEKHDHRDHHHSNESMPCLKIAPYNAHFSFSLYRKIAADNP-TE 44

A0A099Z110 --------------------------------SCRRIVPSNTDFAFRFYRQASAEDP-DK 27

G1NKI3 --DHHNE------KPKVTHLHEQHPHEADSLAFSQHIIPSNTDFAFRFYRQATVQAP-GK 51

E1C206 --DHHNE------EPKATHLHEQHPHEEDSLAFCQHIIPSNTDFAFRFYRQATVQAP-GK 51

R0LEI7 -------------EPQATHLQERHSHEGDPLGFCQRIVPSNTDFAFRFYRQASAQAP-GK 46

A0A091F2Q1 --------------------------------SCQRIVPSNTDFAFQFYRQANTQEA-GK 27

A0A093IN74 --------------------------------SCQRIVPSNTDFAFQFYKQATSQEP-DK 27

A0A093C116 --------------------------------SCKWIIPSNRDFAFRFYRQATTQEP-GK 27

A0A093PVA9 --------------------------------SCQRIVPSNTDFAFRFYRQATTQEP-GK 27

A0A091KNU4 --------------------------------SCKRIVPSNTNFAFRFYRQATTQEP-GK 27

A0A087VMC2 --------------------------------SCQQIVPSNTNFAFRFYRQATTQER-GK 27

A0A091PCC0 ------------------------------------------DFAFRFYRQATTQEP-GK 17

A0A093FFU5 --------------------------------SCQQIVPSNTHFAFQFYRQATTQEP-GK 27

A0A091XH23 --------------------------------SCQQIVPSNTDFAFRFYRQATIQEP-GK 27

A0A091T7I8 ------------------------------------------EFAFRFYRQATTQEP-GK 17

A0A093CLJ8 --------------------------------SCRRIVPSNIDFAFRFYRQATTQEP-GK 27

A0A093KV79 --------------------------------SCQQIVPSNTDFAFRFYRQATTQEP-GK 27

A0A087R545 ------------------------------------------DFAFWFYRQATTQEP-GK 17

G1KWY8 -PGHHNDQETDEN-PAEHQHLHLDEEQDNKTAKCHKIAPSNADFAFTLYRQIASDGA-GK 57

G1NKH5 -----HDPNEPND----------HTHHNAEAVACLKLVPNNADFAFKFLNEVALEEP-NK 44

A0A093H648 -------------------------------MACLKLVPNNADFAFQFFKEVTLETP-NK 28

A0A091PB47 --------------------------------ACLKLVPNNADFAFQFFKEVTLEAP-NK 27

A0A093QJ65 --------------------------------ACLKLVPNNADFAFQFFNKITLEAP-NK 27

A0A091K7I5 --------------------------------ACLKLVPNNADFAFELFKEVALEAP-NE 27

A0A091K0Z5 --------------------------------ACLKLVPNNADFAFQFFREVALEAP-NK 27

R0LHU9 ----HHDPNEPKD----------HVHYAGEAIACLKLVPNNADFAFHFFNKVTLEEP-NK 45

H0ZQY2 -------------------------------MACLKLVPNNADFAFQFFREVTQEAP-NK 28

A0A091GLC8 --------------------------------ACLKLVPNNADFAFQLFKEIALETP-NK 27

A0A091HV30 --------------------------------ACLKLVPNNADFAFQFFKEVTLEAP-DK 27

A0A093RZ50 --------------------------------ACLKLVPNNADFAFQFFKEVTLEAP-DK 27

A0A091SWC2 --------------------------------ACLKLVPNNADFAFQLFKEVILEVP-NR 27

A0A091QU84 --------------------------------ACLKLVPNNADFAFQFFKEVTLDSP-DK 27

A0A093GS00 --------------------------------ACLKLVPNNADFAFRFFKEVTLEAP-NK 27

A0A093IEQ9 --------------------------------ACLKLVPNNADFAFQLFKEVTLEAP-NK 27

A0A091PLB4 --------------------------------ACLKLVPNNADFAFHFFKEVTLEAP-NK 27

A0A093FVA3 --------------------------------ACLKLVPNNADFAFQFFKEVTLEAP-NK 27

A0A091U6W4 --------------------------------ACLKLVPNNADFAFQFFKEVTLEAP-NK 27

A0A091KQX5 --------------------------------ACLKLVPNNADFAFQFFKEVTLEAP-NK 27

A0A091MK58 --------------------------------ACIKLVPNNADFAFQFFKEVTLEAP-NK 27

A0A091T7N1 --------------------------------ACLKLVPNNADFAFQFFKEVSLEAP-NK 27

A0A0A0A7A0 --------------------------------ACLKLVPNNADFAFQFFKEVTLEAP-NK 27

A0A091VAK7 --------------------------------ACLKLVPSNADFAFQFFKEVTLEAP-NK 27

A0A093P9B6 --------------------------------ACLKLVPNNADFAFQFFKEVTLEAP-NK 27

K7FR61 --DHHNGHGDHKD----------TDHEESHKNHASKLSPCNADFAFRIFKQIASDAP-EK 47

M7B3F1 ---------------------------------CLKLAPSNADFAFRLYKQITSEAP-KK 26

K7FUR6 -------QDDQK-------DTNHTEEHALLNQACVKQGSSYADFTFRFYKQAVLEEA-DK 45

Q8JIA6 --------------------ETHAGAPPSKNMTCHKIAPSNADFAFRFYKQIAAEAP-AK 39

A0A093H779 -----------------------------------KLASSNTDFAFAFYKLVTSKAT-DK 24

G1NKH1 ---------------------------PNKNKTFVKVVHSNADFAFSFYKLVASEAT-DQ 32

U3JIK3 -----------------------EEASPLKNKTFVKLVFSNADFAFSFYKLVASEAM-DK 36

A0A091EGC4 -----------------------------------KLVFSNADFAFSFYKLVASEAT-DK 24

A0A093PHW0 -----------------------------------KLVFSNTDFAFSFYKLVASEAM-DQ 24

A0A091NZH9 ---------------------------------------SNADFAFSFYKLVASEAT-DQ 20

R0JRB7 ---------DPKGAYYPGHSSHGEGAYPDKNKTFVKIAPSNADFAFSFYKLVASEAT-DQ 50

A0A091KRT6 -----------------------------------KLLPSNADFAFSFYKLITSETT-DQ 24

A0A091FX98 -----------------------------------KLVPSNADFAFSFYKLVSLQET-NQ 24

A0A091LT78 -----------------------------------KLVPSNTDFAFSFYKLVSSEAT-DQ 24

A0A091QJI6 -----------------------------------KLVPSNADFAFSFYKLVTSEAT-NQ 24

A0A093H5C6 -----------------------------------KLVPSNADFAFSFYKLVTSEAT-DQ 24

A0A091RAD2 -----------------------------------KLIPSNADFAFSFYKLVASEAT-DQ 24

A0A091KQF0 -----------------------------------KLVPSNADFAFLFYKLIASEAT-HQ 24

A0A093IZH1 -----------------------------------KLVLSNAEFAFSFYKLVASEET-DQ 24

A0A091V9J5 --------------------------------AFVKLVPSNADFAFSFYKLVASEET-DQ 27

K7FUQ7 -----------------------------EGKAYFKLAHSNVAFAFKFYKQVISEAT-DK 30

M7B3E7 --DHRNDWGGHKDASTREHNCPVEEVYPCESKAYFKLAPSNIDFAFRFYKQVISEAA-DK 57

K7FUC4 --EHH----------P-----RVEENPKSKIPACCKIAASNADFAFRFYKQVTSDAA-EK 42

K7FSH8 VQPHHVPDHHDDKDSQKVQLCPVGADVRSENTTFLRVAPSNADFAFRFYKQIKSEAG-NK 59

M7B5J6 --EHHNDQDGQTDNNPPQQLSPVGGKVVTEHMTFMKVVASNADFAFRFYKQIKSEAA-DK 57

S7ME51 ----------QADGPQ-------EGPGAEEVSPSLQIAPSNAAFAFNFYHLVASLNP-GS 42

M3XVL7 VNPEHYRQEHTGNPHQ-------EASSTGEGSPSLKIAPGNTAFALHFYHLMASQSP-GS 52

K9IXP7 --------PDQMNVTQ-------EHQHTETPVDLLRFASSNTDFAFSLYRQLALKNP-NK 44

A0A0D9REC0 ---HPNCPLDKENPTQ-------EDQDRGTHVD-LGLASTNVDFAFSLYKQFVLKAP-DK 48

K7A8Q9 ---HPNSPLDEENPTQ-------ENQDRGTHVD-LGLASANVDFAFSLYKQLVLKAP-DK 48

Q5R536 ---HPNCPLDEENPTQ-------ENQDRGTHVD-LGLASTNVDFAFSLYKQLVLKAP-DK 48

A2I7N2 -------HCLPENVTP-------EEQHKVTSVDGHSLASSNTDFAFSLYKQLALKDP-NK 45

F1SCC6 ----PADDLASKTVTL-------KGQITKLPAHNTAVVSSNTDFAFSLYKQLVSLDP-NK 48

F6SWD3 -------------------------------LPSYQIAQSNRDFAFSLYKQLVTEGP-GK 28

F7CZD2 ----LGHGSHRDWPPQ-------DSQDPKEALPYHQVATSNGNFAFRLYKQLISERP-DK 48

A0A091CQE0 -----------AEDTQ-VTNAHSHDKK---HLSCHKIAPSLAEFAFSTYRVLAQQSN-TS 44

G5B496 -----------------ETHAHSHDLE---HLSGHKIAPSLAEFAFSIYQVLAQQSN-TS 39

H0WJ03 --------DPQGDTSQ---DTSKHEHN---LPACHQIAPNLAEFALSLYRHLAHQSN-TT 45

U3FMP8 --------DPQGDAAQ-KMDTSHHDHD---HQTHNKIAPNLAEFTLRLYRQLAHQSN-TT 47

1qlp -------MDPQGDAAQ-KTDTSHHDQD---HPTFNKITPNLAEFAFSLYRQLAHQSN-ST 48

G1S644 --------DPQGDAAQ-KTDTSHHDQD---HPTFNKIAPKLAEFAFSLYRQLAHQSN-SS 47

P01010 --------DPQGDAAQ-KTDTPPHDQN---HPTLNKITPSLAEFAFSLYRQLAHQSN-ST 47

F7CYP1 --------DLQGDAVP-ERHATKDDNEHPQEPAEHKIAPNLADFAFSLYRHVAHQSN-TT 50

B5BV12 --------DLQGCAVQ-ETHATAHDEEHLQEPAEHKIAPNLADFAFSLYRHVAHQSN-TT 50

B5BV05 --------DLQGCAVQ-ETHATAHDEEHLQEPAEHKIAPNLADFAFSLYRHVAHQSN-TT 50

L8YAZ3 --------DSHGDAAQ-ETDASGNEHD---HLSCHRIAPHLSNFALRLYQEVAQKSN-TT 47

G3TDW3 --------SPEGDPAQ-ETGSSKCDHDQ--HQARLDISPSLVNFAFSLYREVARESN-TT 48

G1PM73 ----------QGVAVQ-DTDAAEHDH----ESASHKIAPNLADFAFSLYRQMANQST-TT 44

L5LNJ6 --------DNQGVTVQ-DTDAAEHDH----ESASHKIAPNLADFAFSLYRQVAHQSN-TT 46

L5JP19 ----------QGVAVQ-ET------DEHDQKGAIYKIAPNLVDFAFSLYRQVAHQSN-TS 42

M3WCX1 ----------QGAAVQ-ETVASQHDHEHHEAPACHKIAPNLADFAFSMYRQVAHESN-HT 48

F1PCE5 ----------QGDAVQ-ETDAPHHDPEH--QPACHKIAPNLADFAFSLYRQVAQESN-TT 46

M3XVV7 -------------------------------PACHKIAPNLADFAFSMYRQVAHGSN-TT 28

D2HEM3 --------DLQGDAVQ-DTDASQHDHEHHAEPACHKIAPNLADFAFRMYRQVAHESN-KT 50

P50447 ----------QGHAVQ-ETDVPRHDHEQHQEAACHRIAPNLADFAFSLYRQVARQSN-TS 48

W5PZS7 ----------QGHAVQ-ET-----DDTSHQEAACHKIAPNLANFAFSIYHKLAHQSN-TS 43

P34955 ----------QGHAVQ-ET-----DDTSHQEAACHKIAPNLANFAFSIYHHLAHQSN-TS 43

P23035 -----------ADEAQ-ETAVSSHEQD---HPACHRIAPSLAEFALSLYREVAHESN-TT 44

Q07298 -----------ADEAQ-ETAVSSHEQD---HPACHRIAPSLAEFALSLYREVAHESN-TT 44

O62663 -----------ADEAQ-ETAVSSHEQD---HPACHRIAPSLAEFALSLYREVAHESN-TT 44

O54761 -----------AEDAQ-ETGASKHDQE---HPASHRIAPNLAEFALSLYRVLAHESN-TT 44

O54763 -----------AGDAQ-ETDASKDDHE---HPACHKIAPNLAEFAFDLYRVLARQSN-TT 44

Q76HP1 -----------AQDAQ-ETEASKQDQE---HPASHRIAPHLAEFALSLYRVLARQSN-TT 44

Q76HP0 -----------AQDAQ-ETEASKQDQE---HPASHRIAPHLAEFALSLYRVLARQSN-TT 44

Q76HN9 -----------AQDAQ-ETEASKQDQE---HPASHRIAPHLAEFALSLYRVLARQSN-TT 44

O54760 -----------AQDAQ-ETEASKQDQE---HPASHKIAPHLAEFALSFYRVLARQSN-TT 44

Q64118 -----------AEDAE-KTDSSHQ---------DHIMASNLADFAFGLYRVLSHQSN-TT 38

P97277 -----------AEDAQ-ETDASKQDQE---HQACCKIAPNLADFSFNLYRELVHQSN-TT 44

G3I296 -----------AEDAQ-ETDASQQDQE---HQACCKIASNLADFALSLYRELVHESN-TT 44

P17475 -----------AEDAQ-ETDTSQQDQS----PTYRKISSNLADFAFSLYRELVHQSN-TS 43

Q63969 -----------AEDVQ-ETDTSQKDQS----PASHEMATNLGDFAFSLYRELVHQSN-TS 43

P07758 -----------AEDVQ-ETDTSQKDQS----PASHEIATNLGDFAISLYRELVHQSN-TS 43

Q00898 -----------AEDVQ-ETDTSQKDQS----PASHEIATNLGDFAISLYRELVHQSN-TS 43

* : .

A0A093GX57 NIFFSPLSLSAAFAMLSLGARAATRQQLHKALAFNLTEIQEWEVHEGFQRLLQLLNDPHR 97

A0A099YYG0 NIFFSPLSISAAFAMLSLGARAATCHELHKGLTFNMTEMEEREVHEGFRHLLQLLNDPHR 97

G1NKH9 NIFFSPLSLSTAFAMLSLGARSNTLSQLYKCLSFNLTEMEEQEIHEGFQDLLQLLNDPHR 107

U3IXC3 NVFFSPLSLSTAFAMLSLGARAHTLSQLHKCLAFNLTELEEREIHHSFQRLLQLLNDPHR 103

A0A091KYD1 NIFFSPLSISIAFATLSLGARSNTLRELHKGLAFNLTE---KEIHEGFQHALQLLNDPNR 95

A0A091QVX2 NIFFSPLSISAAFAMLSLGARSNTLLQLQKGLAFNMTEIEEQEIHKGFQQVLQLLNEPHQ 92

A0A091NC43 NIFFSPLSISTAFAMLSLGARSSTLSQLHEGLAFNLTEVEEQEIHEGFQRVLQLLNDSHQ 97

A0A091GSL7 NIFFSPLSISTAFAMLSLGARSNTLNQLHKGLAFNLTEMEEQEIHEGFQRVLQLLNEPGQ 95

A0A093PGJ8 NIFFSPLSISTAFAMLSLGARSNTLRELHKGLFFNQTEMEKQEIHEGFRCVLQLLSDPHR 97

A0A091NIR3 NIFFSPLSISTAFAMLSLGARSNTLRQLHKGLAFNLTEREEQEIHEGFQHVLQLLNDPHR 97

A0A091G0Q5 NIFFSPLSISTAFTMLSLGARSNTLSQLHKGLAFNLTEMEEQEIHEGFQRVLQMLNNPHQ 97

A0A093GVB9 NIFFSPLSISTAFAMLSLGARSNTLSQLHKGLVFNLTEMEEQEIHQGFQRILQLLNNPQR 95

A0A093CUY6 NIFFSPLSISTAFAMLSLGARSNTLSQLHKGLAFNLTEMEEQEIHEGFQRVLQLLNDPHR 97

A0A091QM83 NIFFSPLSISTAFAMLCLGAKSNTLNQLHKGLAFNLTEIEEQEIHEGFQHVLRLLNDPHQ 97

A0A091PDX7 NIFFSPLSISTAFAMLSLGARSNTLGQLHKGLAFNLTEIEEQDIHEGFQRILQLLNDPHR 97

A0A091WBP3 NIFFSPLSISTAFSMLSLGARANTLSQLHKGLAFNLTEMEEQEIHEGFQRVLQLLNDPHR 97

A0A091SNX6 NIFFSPLSIATAFAMLSLGARSNTLSELQKGLAFNLTEMEEQEIHEGFQHILQLLNDPHR 97

A0A094KEC1 NIFFSPLSISTAFAMLSLGARSNTLRQLHKGLAFNLTKMEQQEIHQGFQHVLQLLNDPHR 97

A0A0A0ABV8 NIFFSPLSISTAFAMLSLGARSNTLNQLHKGLAFNLTEVEEQEIHVGFQRVLQLLNDPHR 97

A0A093JEM7 NIFFSPLSISTAFAMLSLGARSNTLRQMHKGLTYNLTEMEEQEIHAGFQRVLQLLNDPHR 95

A0A093G1U7 NIFFSPLSISTAFAMLSLGARSNTLNELHKGLAFNLTEVEEQEIHEGFKHVLQLLNDPHR 97

A0A091JCG9 NIFFSPLSISTAFTMLSLGARSNTLSQLYKGLAFNLTELEEQEIHKGFQRVLQLLNDPYR 97

A0A091U8N8 NIFFSPLSISTAFAMLSLGARSNTRSQLHKGLAFNLTEMEEQEIHEGFQRVLQLLNDPHR 97

A0A091RXN3 NIFFSPLSISTAFAMLSLGARSNTLNQLHKGLAFSLTEMEEPEIHEGFHHVLQLLNDPHR 95

A0A093R765 NILFSPLSISTAFAMLSLGARSNTLSQLHKGLAFNLTEMEEQEIHEGFHRVLQLMNDPHR 97

A0A087VMC1 NIFFSPLSISTAFAMLSLGARSNTLKQLHKGLAFNLTDMEEREIHEGFQHVLQLLNDPHR 97

A0A093F995 NIFFSPLSISTAFAMLSLGARSNTLSQLHKGLTFNLTEMEEQEIHEGFQRVLQLLNDPHR 97

A0A093DUW7 NIFFSPLSISTAFAMLSLGARSNTLSQLHKGLTFNLTEMEEQEIHEGFQRVLQLLNDPHR 97

A0A091T9D5 NIFFSPLSISTAFAMLSLGARSNTLSQLHKGLAFNLTEMEEKDIHEGFQCVLQLLNNPHR 97

A0A091LUF5 NIFFSPLSISTAFAMVSLGARTNTLSQLHKGLAFNLTEMEQQEIHQGFQHVLQLLNDPHR 97

A0A091V8N3 NIFFSPLSISTAFAMLSLGARSNTLSQLHKGLAFNLTEIEEQEIHEGFQRLLQLLNDPHR 97

A0A091LFJ1 NIFFSPLSISTAFAMLSLGARSNTLSQLHKGLAFNLTEMEEQEIHKGFQRVLQLLNDPHR 97

A0A087R544 NIFFSPLSISTAFAMLSLGARSNTLSQLHKGLAFNLTEMEEQEIHEGFQYVLQLLNDPHR 97

A0A091LM39 NIFFSPLSISTAFAMLSLGARSNTLSQLHKGLAFNLTEMEEQEIHEGFQCVLQLLNDPHR 97

Q66KX6 NIFLSPVSISTAFAMLSLGARGQTLKQITEGLSFNTTEISEEEIHKGFQHLLHMLNDPNS 104

A0A099Z110 NIFFSPVSISVGFALLALGSRSSTQAQVLEGLAFNLTEIQEEEIHRGFRHLLLELNRPDS 87

G1NKI3 NVFFSPVSVSAAFALLALGSRATTQAQLLEGLAFNLTNNREEEIHRGFHHLLLLLNRPGR 111

E1C206 NIFFSPVSVSAAFALLALGSRAATQAQLLEGLAFNLTNNREEEIHRGFHHLLLLLNRPGS 111

R0LEI7 NIFFSPVSVSAAFALLALGSRAASRVQVLEGLAFNLTSTREEEIHDGFRHLLSLLNRPGS 106

A0A091F2Q1 NIFFSPVSISAAFALLALGSRGTSQAQVLEGLAFNLTTIQKKEIHEGFHHLLLLLNRPGS 87

A0A093IN74 NVFFSPVSISTAFALLALGSRATSRAQVLEGLAFNLTDIREEEIHDSFRHLLLLLNGPGS 87

A0A093C116 NIFFSPVSISAAFALLALGSRATSQAQVLEGLSFNLTSTREEEIHSGFHHLLLLLTRPGS 87

A0A093PVA9 NIFFSPVSISAAFALLALGSRATSQAQVLEGLAFNLTNTREEEIHDGFRHLLLLLTRPGN 87

A0A091KNU4 NIFFSPVSISTAFALLALGSRATSQAQVLEGLAFNLTDTQEEEIHNI-HHRLLLLTRPGS 86

A0A087VMC2 NIFFSPISISTAFALLALGSRATSQAQVLEGLAFNLTNTHEEEIHNGFHHLLFLLNHPGS 87

A0A091PCC0 NVFFSPVSISTAFALLALGSRATSQAQVLEALAFNLTNTREEEIHDGFHHLLLLLKRPGS 77

A0A093FFU5 NIFFSPVSISTAFALLALGSRATSQAQVLEGLAFNLTDTQEEEIHNGFCH-------PGS 80

A0A091XH23 NIFFSPVSISIAFALLALGSRATSQAQVLEGLAFNLTNTREEEIHNGFRHLLLPLNRPGS 87

A0A091T7I8 NIFFSPVSISTAFALLALGSRATSQAQVLEGLAFNLTNTREEEIHNGFRHLLLFLNRPGS 77

A0A093CLJ8 NIFFSPVSISTAFALLALGSRANSQTQVLEGLAFNLTNTREAEIHDGFHHLLLLLNRPGS 87

A0A093KV79 NIFFSPVSISTAFALLALGSRATSQTQVLEGLAFNLTDTREEEIHDGFRHLLLLLTRPGS 87

A0A087R545 NVFFSPVSISTAFALLALGSSATSQAQVGEGLAFNLTDTREEEIHEGFRHLLLLLNRPGS 77

G1KWY8 NVFFSPVSISTAFAMLSLGAKSETQSQIHKGLAFNLSEMEEKELHEGFQHLIHMLNRPKN 117

G1NKH5 NIFFSPVSISTTFAMLALGARSITKTQILEGLAFNLTEIQENEIHEGFHNLVHMLSHPES 104

A0A093H648 NIFFSPVSISAAFAMLAFGARSATQTQILEGLAFNLTEMHEKEIHEGFHHLIHMLSHPES 88

A0A091PB47 NIFFSPVSISAAFAMLALGARSATQTQILEGLAFNLTEIQEKEIHEGFHNLLHMLSHPEG 87

A0A093QJ65 NIIFSPVSISIAFAMLALGARSATQTQILEGLAFNLTEIQEKEIHEGFHNLLHMLSHPGS 87

A0A091K7I5 NIFFSPVSISSAFAMLALGARSTTQTQILEGLTFNLTEIQEKEIHEGFHNLIHMLSDPEN 87

A0A091K0Z5 NIFFSPVSISTAFAMLALGARSTTQIQILEGLSFNLTEIHEKEVQEGFHNLFHMLSHPES 87

R0LHU9 NIFFSPVSISTAFAMLALGARSTTQTQILEGLAFNLTEIQEKEIHEGFHNLIHMLSHPES 105

H0ZQY2 NIFYSPVSISAAFAMLALGARSATQSQILEGLAFNLTEIQEKEIHEGFHNLIHMLNHPEG 88

A0A091GLC8 NIFFSPVSISTAFAMLALGARSTTQTQILEGLAFNLTEIQEKEIHEGFHNLIHMLSHPEN 87

A0A091HV30 NIFFSPVSISIAFAMLGLGARSDTRSQILEGLAFNLTEIQESEIHEGFHNLIHMLSHPES 87

A0A093RZ50 NIFFSPVSISSAFAMLALGARSATQTQILEGLAFNLTEIEEKEIHEGFHNLIHMLSHPEG 87

A0A091SWC2 NIFFSPVSISTAFAMLALGARSTTQTQILEGLTFNLTEIQEKEIQEGFHNLIHMLSHPES 87

A0A091QU84 NIFFSPVSISTAFAMLALGARSTTQTQILEGLAFNLTEIQKKEIHEGFQNLIHMLSHPES 87

A0A093GS00 NIFFSPVSISTAFAMLALGARSTTQTQILEGLAFNLTEIQEKEIHEGFHNLIHMLSHPEN 87

A0A093IEQ9 NIFFSPVSISTAFAMLALGARSTTQIQILEGLAFNLTEIQEKEVHEGFHNLIHMLSHPES 87

A0A091PLB4 NIFFSPVSISTAFAMLALGARSTTQTQILEGLAFNLTEIQEKEIHEGFHNLIHMLSHPEG 87

A0A093FVA3 NIFFSPVSISTTFAMLALGARSTTQTQILEGLAFNLTEIQEKEIHEGFHNLYHMLSHPEN 87

A0A091U6W4 NIFFSPVSISTAFAMLALGARSTTQTQILEGLTFNLTEIQEKEIHEGFHNLIHMLSHPES 87

A0A091KQX5 NIFFSPVSISTAFAMLALGARSTTHTQILEGLAFNLTEIQEKEIHEGFHNLFHMLSHPES 87

A0A091MK58 NIFFSPVSISTAFAMLALGARSTTQTQILEGLAFNLTEIQEKEIHEGFHNLIHMLSHPES 87

A0A091T7N1 NIFFSPVSISTAFAMLALGARSTTQTQILEGLAFNLTEIQEKEIHEGFHNLVHMLSHPQS 87

A0A0A0A7A0 NIFFSPVSISAAFAMLALGARSATQTQILEGLAFNLTEIQEKEIHEGFHNLIHMLSHPEN 87

A0A091VAK7 NIFFSPVSISTAFAMLALGARSTTQTQILEGLTFNLTEIQEKEIHEGFHNLIHMLSHPES 87

A0A093P9B6 NIFFSPVSISTAFAMLALGARSTTQTQILEGLTFNLTEIQEKEIHEGFHNLVHMLSHPES 87

K7FR61 NVFLSPISISTAFAMLALSAKSATLTQILEGLAFNLTELQEQEIHDSFHHLIHMLNHPDR 107

M7B3F1 NIFFSPVSISTAFAMLALGAKSATLTQILEGLAFNLTEVQEKEIHHGFHGLIHILNHPDG 86

K7FUR6 NVFFSPMSIATAFAMLAMGAKSATLTQIFEGMGFNLTEIQEEEIHESFRHQLRMMNCPNN 105

Q8JIA6 NVFFSPVSISTAFAMLTLGAKSTTQSQIYEGLAFNLTEIEEQEIHEGFRHFIQMFSLSDR 99

A0A093H779 NIFFSPISISTSFAMVALGAKSATLMQVLDGLAFNLKKTQEEEIHEGFCQLLSMLNRPES 84

G1NKH1 NIFFSPISISASLAMLALGAKSATLTQILEGLAFNLKKTQDQEIHEGFCQLLHMLNRSDS 92

U3JIK3 NIFFSPISISASFAMLALGAKAVTLTQILEGLAFNLKKTQEQEIHEGFCQLLHSLNRSDS 96

A0A091EGC4 NIFFSPISISASFAMLALGAKAVTLTQILEGLAFNLKKTQEQEIHEGFCQLLHMLTRSDS 84

A0A093PHW0 NIFFSPMSISASFAMLALGAKSVTLTQILEGLAFNLKKTQELEIHDNFCQLLHMLIRSDS 84

A0A091NZH9 NIFFSPVSISASFAMLALGAKSATLTQILEGLAFNLKKTQEQEIHEGFCQLVHMLXXX-- 78

R0JRB7 NIFFSPISISASFAMLALGAKSATLTQILEGLAFNLKKTQEQEIHEGFCQLLHMLNRSDG 110

A0A091KRT6 NIFFSPISISVSFAMLALGAKSETLMQILEGLAFNLKKTQEQEIHEGFCQLLHLLNRSDS 84

A0A091FX98 NIFFSPISISVSFAMLALGAKSATLTQILEGLAFNLKKTQEEEIHEGFCQLLHLLTRSDC 84

A0A091LT78 NIFFSPISISASFAILALGAKSATLAQILEGLAFNLKKTQEQEIHEGFCQLLHIK---IS 81

A0A091QJI6 NIFFSPISISASFAMLALGAKSATLVQILEGLAINLRKTQEQEIHEGFCQLLHMLD---- 80

A0A093H5C6 NIFFSPISISASFAMLALGAKAATLTQILEGLAFNLKKTQEQQIHEGFCQSLHTLNRSES 84

A0A091RAD2 NIFFSPISISASFAMLALGAKSATLTQILEGLAFNLKKTQEQEIHEGFCQLLHML----- 79

A0A091KQF0 NIFFSPISISASFAMLALGAKTATLTQILEGLAFNLKKIQELEIHEGFCQLLHMLTRSDS 84

A0A093IZH1 NIFFSPISISASFAMLALGAKSATLTQILEGLAFNLKKIQDQEVHEGFCQLLRMLNGSDS 84

A0A091V9J5 NIFFSPISISASFAMLALGAKSATLTQILEGLAFNLGKTQEQEIHEGFCQLLHMLNRSDS 87

K7FUQ7 NIFFSPVSISTSFAMLALGAKSDTLDQIKKGLTFDLNKTQEKEIHEGFCHLVHVLNRPDK 90

M7B3E7 NIFFSPISISTAFAMLALGAKSATLNQIHKGLTFDLNKTQEKEIHEGFCHLICALNRPDR 117

K7FUC4 NIFFSPLSISTAFAMVTLGAKSTTLTQILEGLAFNLTEIEEQEIHEAFRDVIHSLNHPDN 102

K7FSH8 NIFFSPLSIATAFAMLTLSAKSSTLSQIHTGLSFNLTEIEEREIHEGFRDLILILNRPDS 119

M7B5J6 NIFFSPLSISTAFAMLTLGAKSATLSQIHKGLSFNLTEIEEREIHEGFHRLLQMLNHPDS 117

S7ME51 NIFFSPLSISAAYAMLALGAREDTWAQILQGLAFNLTDTSEPDIHRGFQHLLHTLNLPHD 102

M3XVL7 NIFFSPLSISASYAMLSLGARSHSQTQILEGLGFNLTELSEQDIHRGFQHLLHTLHLPGN 112

K9IXP7 NVVFSPMSISMALAFLSLGARDTTLTEILRGLKFNLTETSETEIHQGFQHLLRALSQPSN 104

A0A0D9REC0 NVIFSPLSISTALAFLSLGAHNTTLMEILRGLKFNLTETSEAEIHQSFQHLLRTLNQSSD 108

K7A8Q9 NVIFSPLSISTALAFLSLGAHNTTLTEILKGLKFNLTETSEAEIHQSFQHLLRTLNQSSD 108

Q5R536 NVIFSPLSISTALAFLSLGAHNTTLTEILTGLRFNLTETSEAEIHQSFQHLLRTLNQSSD 108

A2I7N2 NVIFSPLSVSIALAFLSLGAHGPTVTEILEGLKFNLTETPETEIHQGFQHLLQTFNQPSN 105

F1SCC6 NVIFSPLSVSIALAFLSLGARGSTLTELLEGLKFNLTKTPEAEIHQGFQHLLRALSQPSN 108

F6SWD3 NVFFSPLSISTSLAMLSLGAQSATLTQILEGLCFNLSHISEREIHEGFQHIVRTLNLTSN 88

F7CZD2 NVFLSPLSISTTLAMLSLGARSATLTQMLEGLHFNLTQISEREIHDGFQHIIRTLNLHNQ 108

A0A091CQE0 NIFFSPVSIASALAMLSLGAKGDTHDQILQGLQFNLTKTAEDDIHNGFKDLLHILNTPNS 104

G5B496 NIFFSPVSIASALAMLSLGAQGDTHAQILQGLEFNLTEVAEADIHKGFKDLLHILNTPNS 99

H0WJ03 NIFFSPMSVATAFAMLSLGAKADTHTQMLEGLHFNLTTTHEDEIHEGFRELLHTLNQPDN 105

U3FMP8 NIFFSPMSIATAFAMLSLGTKADTHTEILEGLNFNLTETPEAQVHEGFQELLRTLNQPDS 107

1qlp NIFFSPVSIATAFAMLSLGTKADTHDEILEGLNFNLTEIPEAQIHEGFQELLRTLNQPDS 108

G1S644 NIFFSPVSIATAFAMLSLGTKADTHDEILEGLNFNVTEIPEAQVHEGFQELLRTLNQPDS 107

P01010 NIFFSPVSIATAFAMLSLGTKADTHSEILEGLNFNLTEIPEAQVHEGFQELLRTLNKPDS 107

F7CYP1 NIFFSPVSIATAFALLSLGAKGDTHTQILEGLRFNLTELAEAQIHDGFQHLLNALNHSDN 110

B5BV12 NIFFSPVSIATAFALLSLGAKGDTHTQILEGLSFNLTELAEAQIHDGFQHLLRVLNHSDN 110

B5BV05 NIFFSPVSIATAFALLSLGAKGDTHTQILEGLSFNLTELAEAQIHDGFQHLLNALDHSDN 110

L8YAZ3 NIFFSPVGIATAFAMLSLGTKADTHTQILEGLDFNLTEKAEADVHRGFHNLLETLNRPDN 107

G3TDW3 NIFFSPVSIAVAFALLSLGAKSDTHTQILEGLKFNLTKTPEAEIHEGFQHLLQTLNQPDS 108

G1PM73 NIFFSPVSIATAFAMLSLGAKGDTHTQILEGLDFNLTERAEADIHRGFENLLHTLNQPDN 104

L5LNJ6 NIFFSPVSIATVFAMLSLGAKGDTHTQILEGLNFNLTERAEADIHRGFENLLHTLNQPDN 106

L5JP19 NIFFSPVSIATAFATLSLGTKGDTHTQILEGLDFNLTERAEADIHKGFHHLLDALNRPDN 102

M3WCX1 NIFFSPVSIATALAMLSLGSKGDTHTQILEGLGFNLTERAEGEVHEGFQQLLHTLNRPDS 108

F1PCE5 NIFFSPVSIATAFAMLSLGTKGDTHTQIMQGLGFNLTERAEREVHQGFHQLLSILNQPDN 106

M3XVV7 NIFFSPVSIATAFALLSLGAKGDTHSEIMKGLRFNLTERAEGEVHQGFQQLLRTLNHPDN 88

D2HEM3 NIFFSPVSIATAFAMLSLGAKGDTHSQIMKGLGFNLTERAEGEVHQAFQQLLHTLNHPDN 110

P50447 NIFLSPVTIARAFAMLSLGTKGATHAEILEGLQFNLTEKAEAEIHEGFQHLLHTLNQPDN 108

W5PZS7 NIFFSPVSIASAFAMLSLGAKGNTHTEILEGLGFNLTELAEAEIHKGFQHLLHTLNQPNH 103

P34955 NIFFSPVSIASAFAMLSLGAKGNTHTEILKGLGFNLTELAEAEIHKGFQHLLHTLNQPNH 103

P23035 NIFFSPVSIALAFAMLSLGAKGDTHTQVLEGLKFNLTETAEAQIHDGFRHLLHTVNRPDS 104

Q07298 NIFFSPVSIALAFAMLSLGAKGDTHTQVLEGLKFNLTETAEAQIHDGFRHLLHTVNRPDS 104

O62663 NIFFSPVSISLAFAMLSLGAKGDTHTQVLEGLKFNLTETAEAQIHDGFRHLLHTVNRPDS 104

O54761 NIFFSPVSIAMALASLSLGTKADTHTQIMEGLGFNLTETAESDIHQGFQHLLQTLNKPNS 104

O54763 NIFFSPVSVATALAALSLGTKGDTHTQILEGLDFNLTEMAETDIHQGFQHLLQTLNRPNN 104

Q76HP1 NIFFSPVSIAIALAMLSLGTKGDTHTQILEGLDFNLTEMVEADIHQGFQHLLQTLNRPNT 104

Q76HP0 NIFFSPVSIATALAMLSLGTKGDTHTQILEGLDFNLTEMAEADIHQGFQHLLQTLNRPNT 104

Q76HN9 NIFFSPVSIATALAMLSLGTKGDTHTQILEGLDFNLTEMAEADIHQGFQHLLQTLNRPNT 104

O54760 NIFFSPVSIATALAMLSLGTKGDTHTQILEGLDFNLTEMAEADIHQGFQHLLQTLNRPNT 104

Q64118 NIFLSPLSIATALAMLSLGSKDDTKAQLLQGLHFNLTETSEADIHKGFQHLLKTLNRPDN 98

P97277 NIFFSPVSIATAFAMLSLGTKGVTHTQILEGLGFNLTEIAEAEVHKGFHNLLQTFNRPDN 104

G3I296 NIFFSPVSIATAFAMLSLGTKGATHTQILEGLEFNLTQIAEGEIHKGFHHVLQTLNKPDN 104

P17475 NIFFSPMSITTAFAMLSLGSKGDTRKQILEGLEFNLTQIPEADIHKAFHHLLQTLNRPDS 103

Q63969 NIFFSPVSIATAFALLSLGSKGDTQTQILEGLQFNLTQTSEADIHKVFQHLLQTLNRPDS 103

P07758 NIFFSPVSIATAFAMLSLGSKGDTHTQILEGLQFNLTQTSEADIHKSFQHLLQTLNRPDS 103

Q00898 NIFFSPVSIATAFAMLSLGSKGDTHTQILEGLQFNLTQTSEADIHNSFQHLLQTLNRPDS 103

*:. **: :: : : :.: : :: : . :::

A0A093GX57 EVQLSMGNALFVDDQLKLLKKFVDDVTNFYDSEAISSNFQNASEAKKEINDYIEMKTHGM 157

A0A099YYG0 EVQLSMGNALFVDDQLKLLQKFLDDVTNFYDSEAVSSNFQDISEAKKKINDYIERKTHGK 157

G1NKH9 EIQLNMGNTLFIDERLKLQQKFLDDVTNFYYSEAVSMDFQNSEHAREEINNYIKAKTHGK 167

U3IXC3 EVQLSMGNALFINNELKLRQKFLEDVTTFYDSEAISSDFQNSEKVVNEINNYIKTKTHGK 163

A0A091KYD1 EVQLDIGNALFIDDQVKPLQKFLDDVRNFYYAELISSNFQNPPEAVKEINKYIETKTRGK 155

A0A091QVX2 DVQLSIGNALFIDDRLNLLQKFLDDVTYFYYSEATSSNFQNSAEATKEINNYIDTKTSGK 152

A0A091NC43 EVQLSMGNALFIDDQMKLLQKFLDDVTNFYYSEAISTSFKNSTKATEEINKYIETKTHGK 157

A0A091GSL7 EVQLSMGNALFIHKRLKLLQKFLDDVTNFYYSEAISSNFQNSSEAIKEINKYIETKTHGK 155

A0A093PGJ8 EVQLNMGNALFIDDRLKLLQKFLDDVIHFYYSEAISSNFQNPPEAIKEINEYVERKTHGK 157

A0A091NIR3 DVQLNMGNALFIDGRLQLLEKFLRDVTKFYYSEAISSDFQNSPEAVKEINKYIETKTHSK 157

A0A091G0Q5 EVQLSIGNALFIDDRLKILQKFLDDITNFYYSEVVSSNFQNSAEATKKINDYIETKTQGK 157

A0A093GVB9 EVQLSLGNALFIDDRLKLLQNFLDGVTNFYYSEAISSNFQNLPEATKEINKYIETKTHGK 155

A0A093CUY6 EVQLSMGNALFVDDRLKVLQKFSDDVTNFYYSEAISSNFQNSSKATKEINNYIETKTHGK 157

A0A091QM83 EFQLSMGNGLFIDDRLKLLQKFLDDVTNFYHSEPMYSNFQNSSEALKEINKYIETKTHGK 157

A0A091PDX7 EVQLSMGNALFIDNRMKLLQKFLDDVTNFYYSEAISSNFQNSSEAIKEINNYIDTKTHGK 157

A0A091WBP3 EVQLNMGNALFIDNRLKLLQTFLDDVTNFYHSEAISSNFQNSPEATKEINKYIETKTHGK 157

A0A091SNX6 EVQLSMGNALFIDKRMKLLQKFLDDVTNFYYSEAIPSNFQNSPAALKEINNYTETKTHGK 157

A0A094KEC1 EVQLNMGNALFIDDQLKLLQNFLNDVTNFYYSEAISSNFQNPPEAIKEINKYIETKTHGK 157

A0A0A0ABV8 EVQLSMGNALFVDDELKLLQKFLDDVTNFYYSEGISTSFRNSAEAIKEINNYIETKTHGK 157

A0A093JEM7 EVQLNMGNALFIDDRLKLFQKFLDDVKNFYYSEAISSNFQDLPEATKEINKHIETKTRGK 155

A0A093G1U7 EVQLNMGNILFIDDQLKLLQKFLDDVTNFYYSEAISSKLQNSAEAAKEINKYIETKTHGK 157

A0A091JCG9 DVQLSMGNALFTDERLKLLQKFLGDITNFYYSEAIPSNFQNSPEAIKQINNYTETKTHGK 157

A0A091U8N8 EVQLDMGNALFIDDQLKLLQKFLDDVTKFYYSEAISSNFQNSPEATKEINKYIETKTHGK 157

A0A091RXN3 EVQLNMGNAVFISDQLKLLQKFLDDVTYFYYSEAISSNFQNSLEAIKEINKYIETKTRGK 155

A0A093R765 EVQLSMGNALFIDDRLKLLQKFLDDVTNFYYSEVISSNFQNSPEAIKEINNYIETKTHGK 157

A0A087VMC1 EVQLSMGNALFIDNQLKLLQKFLDDVTNFYYSEAISSNFQNSPEAIKEINDYIETKTHGK 157

A0A093F995 EVQLNMGNALFIDDGLKLLQKFLDDVTNFYYSEAISSNFQNSSEARKEINKYIETKTRGK 157

A0A093DUW7 EVQLSMGNALFIDDRLKLLQKFLDDITNFYYSEAVSSDFQNSPEAVKEINKYIETKTHGK 157

A0A091T9D5 EVQLNMGNALFIDDRLKLLQKFLDDVTNFYYSEAISSNFQNSPEAIKQINKYIETKTQGK 157

A0A091LUF5 EVQLSMGNALFIDDRLKLLQKFLDDVTNFYYSEAVSSNFENSAEAIKEINNHIETKTHGK 157

A0A091V8N3 EVQLSMGNALFIDDRLKLLQKFLDDVTNLYYSEAISSNFQNPPEATKEINNYIETKTHGK 157

A0A091LFJ1 EVQLNMGNALFIDDRLKLLQKFLDDVTNFYYSEAISSNFQNSLEAIKEINNYTETKTHGK 157

A0A087R544 EVQLSIGNALFIDDRLKLLQKFLDDVTNFYYSEAISSNFQNSPEATKEINNYIETKTHGK 157

A0A091LM39 EVQLSMGNALFIDDRLKLLQKFLDDVTNFYYSEPISTNFHNSPEATKEINNYIETKTHGK 157

Q66KX6 EMQLNSGNALFIDKDFQIIQKFVEDAKQFYEAEPVSTDFHNTEEATKQINSYAEKKTNGK 164

A0A099Z110 QMQLSMGNALFMDENVTPLKTFLKDSKNMYKAEVISSNFQNSTEAKNEINDYVRNKTHGK 147

G1NKI3 QVELSMGNTLFVDKHLEPLTTFLKDIKKLYKAEIISSNIQNSTEAKKEINDHLKNKTHGK 171

E1C206 QVELSMGNTLFMDKHLKPLTTFLKDIKKLYKGEIISSNFQNSTEAKKEINEHMKNKTHGK 171

R0LEI7 QVQLSMGNTLFIDEHLKPLKTFLKDIQKTYRGKIISSNFQNSTEAKKVINDHIKNQTHGK 166

A0A091F2Q1 QVQLSMGNTLFMDKHLKPTKTFLKDVQKLYRGKVVSSSFQNSTEAKKEINDYVKNKTHGY 147

A0A093IN74 QVELSMGNTIFMDKNLNPLKSFLKDIKKIYKGKVVSSNFQNSTEAKNQINDHIKNKTHGN 147

A0A093C116 QVQLNMENTLFMDKHLKPRKTFLKDIKKLYKGKVVSSNFQNSTEAIKEINDHIKNKTHGN 147

A0A093PVA9 QVQLSMGNTLFMDNHLKPLKTFLKDIKKLYRGNVVSSNFQNSTEAKKEINDYVKNETHGN 147

A0A091KNU4 QVQLSMGNTLFIDKHLKPLQTFLKDIKKLYKGKVISSNFQNATEAKKDINDHIKNKTHGN 146

A0A087VMC2 QVQLSMGNTVFMDKHLKPLKTFLKDIKKLYKGKVVSSNFQNSTEAKKEINDHIKNKTHGN 147

A0A091PCC0 QGKLSMGNALFMDKQLKPLKTFLKDIKKLYKGKIVSTNFQNSTEAKKEINDHVKNKTHGN 137

A0A093FFU5 QVQLSMGNTLFMDKHLKPQKTFLKDVKQLYKGKIVSSNFQNSTEAKKEINDHIKNETHGN 140

A0A091XH23 QVQLSMGNTMFMDKHLKPLKMFLKDIKKLYKGKVISSNFQNSTEAKKEINEHINNITHGN 147

A0A091T7I8 QVQLSMGNTLFMDKHLKPLNTFLKDIKKLYKGKVVSSNFQNSTEAKKEINDHIRNKTHGN 137

A0A093CLJ8 QVQLSMENTLFMDKHLKPLKKFLRDIKKLYKGKVFSSNFQNSTEAKKEINDHIKNKTHGN 147

A0A093KV79 QVQLSMGNTLFIDKHLKPLKTFLKDIKKLYKGKVVSSNFQNSTEAKKEINDHIKNKTHGN 147

A0A087R545 QVQLSMGNTLFMDKHLKPLKTFLKDIKKLYKGKVVSSNFQNSSEAKKEINEHIKNKTHGN 137

G1KWY8 KAEINIGNALFIEESLKVLPKFLEDAKTLYESEGFSSNFRNTTIAKKQINDYVVNKTHGK 177

G1NKH5 GVQLNMGNAIFLTEKLKPLEKFLDDAKPLYQLQVLATDFNNPTEAEKEINDYIEKKTQGK 164

A0A093H648 EVQLNMGNVMFLTGKLKPLKKFLDDAKALYQMDAFTADFNNPAEAEKQINDYIERKTHGK 148

A0A091PB47 GVQLDMGNAIFVTERLKPLKTFLDDAKALYQLETYTTDFKNPTEAEKQINDYIEKKTHGK 147

A0A093QJ65 GVQLNMGNTIFLTEKLKPLKRFLDDAKSLYQLEVFTADFNNPTETEKQINDYTERKTHGK 147

A0A091K7I5 GVQLNMGNAIFLTEKLKPLKKFLDDAEALYQLEAFTTDFKNPTEAEEQINDYIERKTYGK 147

A0A091K0Z5 GVQLNMGNAIFLTEKLKPLQKFLDDAKALYELEAFTADFNNPVEAEKQINDYIERKTHGK 147

R0LHU9 GVQLNMGSAIFLTEKLKPLKKFLDDAKALYQLETLTTNFNNPTEAEKQINDYIEKKTHGK 165

H0ZQY2 GVQLNMMNAIFVTEKLKLLRKFLDDAKALYQLEAFTTDFNKPTEAEKQINDYIERKTHGK 148

A0A091GLC8 GVQLNMGNAIFLTEKLKPLKQFLDDAEGLYQLEAFTTDFNNPTKVEKQINDYIERKTHGK 147

A0A091HV30 GVQLNMGNAIFLTEKMKPLKKFLDDAKALYQLEAFTTDFNIPVEAEKQINDYVERKTHGK 147

A0A093RZ50 GVQLNMGNAIFVTEKLKPLKKFLDDAKALYQLEAFTTDFNNPPAAEKQINDYIERKTHRK 147

A0A091SWC2 RVQLNMVNAIFLTEKLKPLKPFLDDAKALYQLEAFTTDFNNPREAEKQINDYVEKKTHGK 147

A0A091QU84 GVQLNMGNVIFLTEKLKPLKEFLDDAKALYQLEAFTTDFNNPMEAEKQINDYIEKKTHGK 147

A0A093GS00 GVQLNMGNAIFVTEKLKPLKKFLDDAEALYQLEAFTTDFSNPTEAEKQINDYIETKTHGK 147

A0A093IEQ9 GVQLNMGNAIFLTEKLKPLKKFLDDAKALYQLEAFTTDFNSPTEAEKQINDYIERKTHGK 147

A0A091PLB4 GVQLSMGNAIFLTEKLKPLKKFLDDAKGLYQLEAFTTDFNKPTEAEKQINDYIERKTHGK 147

A0A093FVA3 GVQLNMGNAIFLTEKLRPLKKFLDDAKALYQLEAFTTDFNNPTEAEKQINDYVERKTHGK 147

A0A091U6W4 GVQLNMGNAIFLTEKLKPLKKFLDDAKALYQLEAFTTDFNNPTEAEKQINDYTERKTHGK 147

A0A091KQX5 GVQLNMGNAIFLTEKLKPLKKFLDDAKALYQLEAFTTDFNNPTEAEKQINDYIERKTCGK 147

A0A091MK58 GVQLNMGNVIFLTEKLKPLKKFLDDAKALYQLEAFTTDFNNLTEAEKQINDYIERKTYGK 147

A0A091T7N1 EVQLNMGNAIFLTEKLKPLKKFLDDAKVLYQLEAFTTDFNSPTEAEKQINDYIERKTHGK 147

A0A0A0A7A0 GVQLNMGNAIFLTEKLKPLKKFLDDAKALYQMEAFTIDFNNPTEAKKQVNDYIERKTHGK 147

A0A091VAK7 GVQLNMGNAIFLPERLKPLKKFLDDAKALYQLEAFTTAFNNPMEAEKQINDYIERKTHGK 147

A0A093P9B6 GVQLNMGNAIFLTEKLKPLKKFLDDAKALYQLEAFTTDFNNPTKAEKQINDYIERKTHGK 147

K7FR61 ELQLDMGNALFLKEKLKPLEKFLADVKNLYEAEAFTVNFKNTAEAQKQINDYVEKKTHGK 167

M7B3F1 ELQLNMGNALFLKEKLKTLEKFLADVKNLYEAEAFSINFKNTAEAQKQINDYVEKKTHGK 146

K7FUR6 KIQMSMGNALFLAMGFQPLQKFLDDVKTFYEAEFFSTDFQNSTEAEEKINGYVNKKTHGK 165

Q8JIA6 EILLNMGNALFIDEKLKPIPKFLDDIKSFYASEGFSSNFTDSAEAEKQINDYIKKKTKGE 159

A0A093H779 ELQLSLGSALFIDEALKPLQKFLDDVKRFYESEVFSIDFNNSSGAENQINSYIKEKTNGK 144

G1NKH1 NLHLSLGNALFIEETLKPLQKFLEDAKSFYQSEVLSADFNNSSGAEIQINSYIEEKTNKK 152

U3JIK3 EFQLSLGSALFIEETLKPLQKFLDDVKSFYESEVFSTYFNNSVGAESQINSYIEEKTNGK 156

A0A091EGC4 EFHLSLGNALFIEETLKPLQTFLDDVKSFYESEVFSTDFNNSVGAENQINSYIEEKTNGK 144

A0A093PHW0 EFQLSLGNALFIEETLKPVQKFLDDVKRFYESEVFSSDFNNSVGAENQINSYIEEKTNGK 144

A0A091NZH9 --XXSLGNALFIEETLKPLQKFLDDVKSFYESEVFSTDFNNSVGAENQINSYIEEKTNGK 136

R0JRB7 DLQLSLGNVLFIEDMLKPLQKFLDDVKSFYESEVFSTDFNNSSSAENQINSYIEEKTNGK 170

A0A091KRT6 ELQLSLGNALFIGKTLKPLQKFLDDVKNFYESEVFSTDFNNSSGAEIQINRYIEKKTNGK 144

A0A091FX98 ELQLSLGNALFIEETLKPLQTFLNDVKNFYESEVFSTDFSNSSGAENEINSYIEEKTNGK 144

A0A091LT78 ELQLSRGNALFIEQSLKPLQKFLDDVKSFYESEVFSTDFNNSFGAENQINSYIEEKTNGK 141

A0A091QJI6 -LQLSLGNALFIEETLKPLQKFLDDVKNFYESEVFSTCFNNSSGAENQINSYIEEKTKGK 139

A0A093H5C6 ELQLSLGNALFIEVTLKPLQKFLDDVKSFYDSEVFSTDFNNSSGAENQINSYIEEKTSGK 144

A0A091RAD2 --QLSLGNALFIEETLKPLQKFLDDAKSFYESEVFSADFNNSSGAENQINSYIEEKTNGK 137

A0A091KQF0 ELQLSLGNALFIKETLKPLQKFLDDVKSFYDSEVFSADFNNSSGAENQINSYIEEKTNGK 144

A0A093IZH1 DLQLSLGNALFIEDTLKPLQKFLDDVKSFYESEVFSTDFNNSSGAENQINSYIEEKTNGK 144

A0A091V9J5 ELQLSLGNALFIEETLKPLQKFLDDVKSFYESEVFSSDFNNSSGAENQINSYIEEKTNGK 147

K7FUQ7 NIQVKMGNALFLQKTLKPLNKFLEDLKNFYKSEVFSTDFYNSTNALKQINNYIENKTHGK 150

M7B3E7 EIQLNMGNALFLRETLKPLKKFLEDVKNFYKSEVFSSDFNNSTNAVKQINNYIEKKTHGK 177

K7FUC4 EIQVNLGNALFVDEKLKLLDKFLDDVKSFYEAEALPSNFQNSAETEKQINDYIEKKTHGK 162

K7FSH8 EIQLNMGNALFVDNQLKLLEKFLKDVKSLYASEAFQSNFQNSAEAEKQINDYIEKKTHGK 179

M7B5J6 EIQLNMGNALFVDDRLKLLEKFLADVKSLYESETFPSNFQNSAEAEKQINDYIEKKTHGK 177

S7ME51 KLETRVGSALFLSQDLPLRPRFLNDTAAFYGASLFPADFLDSEGTTQLINSHVKKETRGK 162

M3XVL7 GLEMHMGNALFLSQDLRILPGFLNDSMTFYESKLFLTDFHNSVGTTQLINDHVKEETQGK 172

K9IXP7 LLQLSMGNAMFVHEKLELLGKFRDDAKALYASEAFSTNFQDSAAAKKLINDYVEEKTQGK 164

A0A0D9REC0 GLQLSMGNAMFIEEQLSLLDRFMEDAKRLYGSEAFATDFQDSAVAKKFINDYVKNRTRGK 168

K7A8Q9 ELQLNMGNAMFVEEQLSLLDRFTEDAKRLYGSEAFATDFQDSAAAKKLINDYVKNRTRGK 168

Q5R536 ELQLSMGNAMFVEEQLSLLDRFMEDAKRLYGSEAFATDFQDSAAAKKLINDYVKNRTRGK 168

A2I7N2 QLQLSVGNAIFVQEELKLLDKFIEDARVLYSSEAFPTNFRDPEAAKSLINDYVKNKTQGK 165

F1SCC6 LLQLNVGNAMFVDERLKLLDKFVQDARELYFSEVFSISFKDSDAAVEFINNYVKNKTKGK 168

F6SWD3 DLEINMGNSLFVRKQMKLQEKFQGDLQRLYAAEALSTNFKDSAGAEKQINAYVEKKTRGR 148

F7CZD2 NLETHLGNSLFLDERLTPKEAFLEGIKELYATEAFKTDFQDAVGAEKQINDHVRKETHGK 168

A0A091CQE0 KHQLTTGNGLFVDHNLKLTAKFSEETKNHYHAEAFPANFSNPEEAMKLINTYVQERTKGK 164

G5B496 ERELTTGNGLFVDQTLRFTAKFLEETRNHYHAEAFPVNFSNPEDAVKQLNIYVQRRTKGK 159

H0WJ03 QLQLTTGNGLFISQGLKLVAKFLEDVKKMYHSEAFSVNFQDTEEAKKQINSYVEKGTQGK 165

U3FMP8 QLQLTTGNGLFLNESLKLVDKFLEDVKKLYHSEAFSVNFRDPEKAKKQINDYVEKGTQGK 167

1qlp QLQLTTGNGLFLSEGLKLVDKFLEDVKKLYHSEAFTVNFGDTEEAKKQINDYVEKGTQGK 168

G1S644 QLQLNTGNGLFLNKSLKLVDKFLEDVKKLYHSEAFSVNFGDTEEAKKQINDYVEKGTQGK 167

P01010 QLQLTTGNGLFLNKSLKVVDKFLEDVKNLYHSEAFSVNFEDTEEAKKQINNYVEKGTQGK 167

F7CYP1 QLQLTTGNGLFIDKTLKLVDKFQEDVKNLYHSEAFSINFGDIEEAKKQINDYVENGTQGK 170

B5BV12 QLQLTTGNGLFIDKTLKLVDKFQEDIKNLYHSEAFSINFGDIEEAKKQINDYVENGTQGK 170

B5BV05 QLQLTTGNGLFIDESAKLLDKFLEDIKKLYHSEAFSINFRDTEEAKKQINDYVEKGTQGK 170

L8YAZ3 QLQLTTGNGLFINNSMKLVDKFMEDAKKLYHSEAFSVNFKDTESAKEQINKYVEKGTQGK 167

G3TDW3 QLQLTTGNGLFINESLKLVDKFLEDAKKLYHSEAFSINFRDNEAAKKQINDYVEKGTQGK 168

G1PM73 QLQLTTGNGLFIDDSVKLVDKFLEDVKKTYHSKAFSVNFKHSEEAKKQINDFVEKGTQGK 164

L5LNJ6 QLQLTTGNGLFIDDSVKLVGKFLEDVKKTYHSEAFSVNFKHSEEAKKQINDFVEKGTQGK 166

L5JP19 QLQLTTGNGLFIDENAKLVSKFLEDVKKLYHSEAFSVNFKDSEAAKKQINDYVEKGTQGK 162

M3WCX1 QLQLTTGSGLFINESMKLLSKFLEDVKNLYHSEAFSINFGDSQEAKKRINDYVEKGTQGK 168

F1PCE5 QLQLTTGSGLFINDTIKLLNKFLEDVRKLYHSEAFTINFRHTEEAKKQINNYVEKGTQGK 166

M3XVV7 QLQLTTGNGLFIAEGMKLLDKFLEDVKNLYHSEAFSTNFGDTEAAKKQINDYVEKGTQGK 148

D2HEM3 QLQLTTGNGLFVAESVKLLNKFLEDVKSLYNSEAFSINFGDTEAAKKQINDYVEKGTQGK 170

P50447 QLQLTTGNGLFIDEKAKLVPKFLEDVKNLYHSEAFSINFRDTEEAKKCINDYVEKGSQGK 168

W5PZS7 QLQLTTGNGLFINESAKLVDTFLEDVKNLYHSKAFSINFRDADEAKKKINDYVEKGSHGK 163

P34955 QLQLTTGNGLFINESAKLVDTFLEDVKNLYHSEAFSINFRDAEEAKKKINDYVEKGSHGK 163

P23035 ELQLAARNALVVHENLKLQHKFLEDAKNLYQSEAFLVDFRDPEQAKTKINSHVEKGTRGK 164

Q07298 ELQLAAGNALVVHENLKLQHKFLEDAKNLYQSEAFLVDFRDPEQAKTKINSHVEKGTRGK 164

O62663 ELQLAAGNALVVHENLKLQHKFLEDAKNLYQSEAFLVDFRDPEQAKTKINSHVEKGTRGK 164

O54761 QLQLTTGNGLFIDHNLKLLDKFLQDVKNLYHSEAFSTDFTNTEEAKKQINTYVEKGTQGK 164

O54763 QLQLTTGNGLFIDQSLKLADKFLEDVKNLYHSEAFSTNFTDSEEAKKQINGYVEKGTQGK 164

Q76HP1 QLQLTSGNGLFIDRNLKLLDKFLEDVKSLYHSEAFPTNFTNTEEARQQINSYVEKGTQGK 164

Q76HP0 QLQLTSGNGLFIHQNLKLLDKFLEDVKSLYHSEALPTNFTNTEEARQQINSYVEKGTQGK 164

Q76HN9 QLQLTSGNGLFIHQNLKLLDKFLEDVKSLYHSEAFPTNFTNTEEARQQINSYVEKGTQGK 164

O54760 QLQLTSGNGLFIDRNLKLLDKFLEDVKSLYHSEAFSTNFTNTEEARQQINSYVEKGTKGK 164

Q64118 ELQLTTGSSLFVNNSLNLVEKFLEEVKNHYHSEAFFVNFADSEEAKKTINSFVEKATHGK 158

P97277 ELQLTTGNGLFIHNNLKLVDKFLEEVKNDYHSEAFSVNFTDSEEAKKVINGFVEKGTQGK 164

G3I296 ELQLTTGNGLFIHDNLKLVATFLEEAKNNYHSEAFSLNFTDSEEAKKVINAFVEKGTQGK 164

P17475 ELQLNTGNGLFVNKNLKLVEKFLEEVKNNYHSEAFSVNFADSEEAKKVINDYVEKGTQGK 163

Q63969 ELQLSTGNGLFVNNDLKLVEKFLEEAKNHYQSEVFSVNFAKSEEARKMINDFVEKGTQGK 163

P07758 ELQLSTGNGLFVNNDLKLVEKFLEEAKNHYQAEVFSVNFAESEEAKKVINDFVEKGTQGK 163

Q00898 ELQLSTGNGLFVNNDLKLVEKFLEEAKNHYQAEVFSVNFAESEEAKKVINDFVEKGTQGK 163

. :. * * . : . :* . :

A0A093GX57 FVNLLKSLDPYTVMVLVNYIFFK--GYWEQPFSSLLTSDDDFFMDAKKSVKVKMMHRNEA 215

A0A099YYG0 FVDFLKSLDSDTVMILVNYIFFK--GYWEQPFSSLLTKDADFFIDAKKSVKVKMMHRSKV 215

G1NKH9 FVDLLDSLDEDVMMILTNYVYFK--GYWEEPFESYDTRDDDFFVDAEHPVKVKMMYKSTY 225

U3IXC3 FVDFLKNLSPDALMILINYIYFK--GYWEQPFKSFHTRDDDFFVDAKNSVKVKMMHQSKI 221

A0A091KYD1 IVDVLKDLDRDTVMVLANYIFFK--GYWEKPFSNLRTRDDDFLVDAKNSVKVKMMQQNAN 213

A0A091QVX2 IVDLVKSLDADTVMVLVNYVLFK--GYWERPFNNLSTRDDDFLLDANNSVKVKMMHQNKV 210

A0A091NC43 IVDFLRELGESTVMVLVNYIFFK--GYWEKPFSNLATRNDDFFLDAKNSVKVEMMHQSKA 215

A0A091GSL7 IVDLLRRLDADTVMVLVNYIYFK--GYWENPFDSSSTREDDFLLDDKNSVKVKMMHQSNY 213

A0A093PGJ8 IVDLVESLDPNTMMVLVNYIFFK--GSWEKPFNNLNTRDDDFFLDAKNSVKVKMMHQNKA 215

A0A091NIR3 IVDLVKSLDPETVMVLVNYIFFK--GSWERPFSNLATREDDFFLDTKNSVKVKMMHQSKD 215

A0A091G0Q5 IVGLLKNLDPDTVMVLVNYIFFK--GNWERPFNNLITRDDEFFLDAKNSVKVKMMHENKA 215

A0A093GVB9 IVDLLKSLDEDSKMVLVNYIFFK--GYWERPFNKLSTRDDDFLLDDKNSVKVKMMHQNKV 213

A0A093CUY6 IVDLLQSLGPGTVMVLVNYIFFK--GYWETPFNELNTRDDDFWLNAKNSVKVKMMHQNKH 215

A0A091QM83 IVDLLKGLDADTVMVLVNYIFFK--GYWEKPFNNLSTRDYDFKLDDKNSVKVKMMHHSKV 215

A0A091PDX7 IVPLLKKLSSDTKMILVNYIFFK--GYWEKPFKNLNTRDDDFFLDAKNSVKVKMMHQKDV 215

A0A091WBP3 IVDLLQSLDSDTAVVLINYVFFK--GYWEKPFNNLSTRDDDFLLDAKNSVKVKMMHQEKA 215

A0A091SNX6 IVRLLESLSPDTVMVLINYVFFK--GYWEKPFNSLSTRDEDFLLDAKNSVKVKMMHQHKT 215

A0A094KEC1 IVDLFKNLEPDTVIVLVNYIFFK--GYWEKPFNNLVTRDDDFLLDAKNSVKVKMMHQNKA 215

A0A0A0ABV8 IVDLVKTLDPETMMVLVNYIFFK--GYWEKPFNNLATRDDDFMLDAKNSVKVKMMHQSKA 215

A0A093JEM7 IVDLLKSLDPDTVMVLVNYIFFK--GYWEKPFNNLATRDDDFLLDSKNSVKIKMMHQNKP 213

A0A093G1U7 IVDLLKSVDDETMMVLVNYIFFK--GYWEKPFNNLVTRDSDFLLDDKNSVKVKMMHQSKV 215

A0A091JCG9 IVGLLQSLDPDTVMVLVNYIFFK--GYWEKPFNSLSTRDDDFLLDAKNSVKVKMMHQSKA 215

A0A091U8N8 IVDLLESLSPDAVMVLVNYIFFK--GYWEKPFNNLVTRDDDFLLDAKNSVKVKMMHQNKP 215

A0A091RXN3 IVDLLKNLDSDTVVVLVNYIFFK--GYWEKPFNNLYTRDDDFFLDAKNSVKVKMMHQNKG 213

A0A093R765 IVDLFKSLDPDTVMVLVNYIFFK--GYWERPFNSLVTRDDDFLLDAKNSVKVKMMYQNKA 215

A0A087VMC1 IVRLFNSLDKDTVMVLVNYIFFK--GYWEKPFKNFATRDDDFLLDAKNSVKVKMMHQSKN 215

A0A093F995 IVDLLKTLDENTVMVLVNYIFFK--GYWEKPFNNLATRDDDFLLDGKNSVKVKMMHQNKA 215

A0A093DUW7 IVGLLKSLDSDTVMVLVNYIFFK--GYWEKPFNSLTTRDDDFFLDAKNSIKVKMMHQNKV 215

A0A091T9D5 IVDLLKSLDSNAVMVLVNYILFK--GYWEKPFNNLVTRDDDFLLDAKNSVKVKMMHQNKV 215

A0A091LUF5 IVGLLKNLDADTVMVLVNYIFFK--GYWEKPFNNLATRDDDFLLDAKNSVKVKMMHQNKP 215

A0A091V8N3 IVDLLKSLDSGTVMVLVNYIFFK--GYWERPFNNLATRDDDFLLDAKNSVKVKMMHQNKD 215

A0A091LFJ1 IIGLLNSLDPDTVMVLVNYIFFK--GYWEKPFNSFATRENDFLLDAKNSVKVKMMHQNKA 215

A0A087R544 VVGLLKSLDPDTVMVLVNYIFFK--GYWEKPFNNLVTRDNDFLLDAKNSVKVKMMHQNSA 215

A0A091LM39 IVGLLNSLDPDTVMVLVNYIFFK--GYWEKPFNNLATRDEDFLLDAKNSVKVKMMHQNKV 215

Q66KX6 ITELLSTVDEKTLLVLINYIYFR--GQWEKPFEKENTVDGEFHVDKDTVVTVPMMHKNGM 222

A0A099Z110 INRIVKNLAPDTLMVLVNYIYFK--AYWENPFNIKGTRKDHFYVNEKTSVEVEMMHRDGF 205

G1NKI3 INQILKDLDPNSLMVLVNYIYFKGKAYWENPFNMKGTHTDYFYVNAKTLVEVKMMIRDSF 231

E1C206 INQILKDLDPNSLMVLVNYIYFK--AYWENPFNTKGTHKDYFYVNEKTLVEVKMMIRDSF 229

R0LEI7 INQMFTDLDPNSLMVLVNYIYFK--AYWENPFNVKGTRKDYFYVNAKTSVEVKMMTRDSF 224

A0A091F2Q1 INQILEDLDPNTLMVIVNYIYFK--AYWENPFNIKGTHKDYFHVNAKTSAEVEMMVRDGF 205

A0A093IN74 INQILKDLDPSTLIVIVNYIYFK--AYWENPFNIKGTHKEYFYVNAKTSVEVKMMTRDGF 205

A0A093C116 INQILKDLDPNTLMVIINYIYFK--GYWENPFNIKGTRKDYFHVNAKTSAEVEMMAQDGF 205

A0A093PVA9 IKQILKELDPNTLMVIVNYIYFK--AYWENPFSIRGTHKDYFHVNAKTSVEVKMMTRDGF 205

A0A091KNU4 INQIVKDLDQNTLMVIVNYIYFK--AYWENPFNLKGTRKDYFHVNAKTSVEVKMMTRDGF 204

A0A087VMC2 INQILKDLDPNTLMVIVNYIYFK--AYWENPFNIKGTHKDYFHVNAKTSVEVKMMTRDGF 205

A0A091PCC0 INQILKDLDPNTLMVIVNYIYFK--AYWENPFSIKGTRNDYFHVNAKTSVEVKMMTRDGF 195

A0A093FFU5 INQILKDLDPNTLMVIVNYIYFK--AYWENPFNIKGTHKDYFHVNAKTSVEVKMMTRDGF 198

A0A091XH23 INQILKDLDPNTLMVIVNYIYFK--AYWENPFNIKGTHKDYFHVNAKTSVEVKMMTRDGF 205

A0A091T7I8 INQILKDLDPNTLMVIVNYIYFK--AYWENPFNIKGTHKDYFHVNMKNSVEVKMMTRDGF 195

A0A093CLJ8 MNQILKDLDPNTLMVIVNYIYFK--AYWENPFNIKGTHKDYFHVNAKTSVEVKMMTRDGF 205

A0A093KV79 INQILKDLNPNTLMVIVNYIYFK--AYWENPFNIKGTHKDYFHVNAKTSVEVKMMTRDGF 205

A0A087R545 INQILQDLDPNTLMVIVNYIYFK--AYWENPFNIKRTHKDYFHVNAKTSVEVKMMTRDGF 195

G1KWY8 IPQAVDQLDPSTVMVLLNYIFFK--GFWEHPFNTQSITEEDFFVNADTTVKVNMMFQSSH 235

G1NKH5 ITNLVKEIDPQTVMLLASFVFFR--GNWEKAFKPENTKEREFFVDAETTVKVPMMYQVGK 222

A0A093H648 ITSLVKDMDPQTVMLLTSFIFFK--GKWEKPFKPELTEERDFFVDEETTVKVPMMHQTGI 206

A0A091PB47 ITNLVNNMDPQTLMLLASFVFFK--GNWEKPFKPEHTEEREFFVDAETTVKVPMMYQMGR 205

A0A093QJ65 ITDLVKNMDQQTVMLLASFVFFK--GNWENPFKPEHTKERDFFVDAETTVKVPMMHQMGR 205

A0A091K7I5 IKNLIKDMDPQTVMILTSFVFFK--GNWEKPFKPEHTEEREFFVDAKTTVKVPMMHQIGR 205

A0A091K0Z5 ITNLVKDMDPHTVMLLASFIFFK--GDWEKPFKPEHTEEREFFVDGETTVKVPMMHQEGI 205

R0LHU9 ITNLVKEMDPQTVMLLATFVFFR--GNWEKPFKPENTEEREFFVDAETIVKVPMMYQTGR 223

H0ZQY2 ITNLVKDMDPQTVMLLASFVYFK--GNWEKPFEAEHTEEREFFVDAETTVKVPMMYQMGR 206

A0A091GLC8 ITNLVKDMDPQTVMFLTSFVFFK--GNWEKPFKPEHTEVREFFVDAETTVKVPMMHQTGR 205

A0A091HV30 ITNLVKNMDPQTVMLLATFVFFK--GNWEKPFNPEHTEERKFFVDAETTVKVPMMHQTGR 205

A0A093RZ50 ITNLVKDMDPQTVMLLASFVFFK--GNWEKPFKPEDTEEREFFVDAETTVKVPMMYQTGR 205

A0A091SWC2 ITNLVKDMDPQTVMLLGSFVFFK--GNWEKSFKPEHTEEREFFVDAETTVKVPMMHQTGR 205

A0A091QU84 ITNLVKDMDTQTVMLLASFVFFK--GNWEKPFKPEHTEEREFFVDAETTVKVPMMHQTGR 205

A0A093GS00 ITNLVKDMDPQTVMLLASFVFFK--GNWEKPFKPEHTEEREFFVDDETTVKVPMMYQTGR 205

A0A093IEQ9 ITNLVKDMDPQTVMLLASFVFFK--GNWEKPFNSEYTEEREFFVDAETTVKVPMMHQTGR 205

A0A091PLB4 ITNLVKDMNPQTVMLLASFVFFK--GNWEKPFKPEHTEEREFFVDAETTVKVPMMYQMGR 205

A0A093FVA3 ITNLVKDMDPQTVMLLASFVFFK--GNWEKPFNPEHTEEREFFVDDETTVKVPMMHQMGR 205

A0A091U6W4 ITNLVKDMDPQTVMLMASFVFFK--GNWEKPFKPEHTEVREFFVDAETTVKVPMMHQTGT 205

A0A091KQX5 ITNLVKDMDPQTVMLLASFVFFK--GNWEKPFKSELTEEREFFVDAETSVKVPMMQQTGR 205

A0A091MK58 ITNMVKDMDPQTVMLLASFVFFK--GNWEKPFKPEHTEEREFFVDAETTVKVPMMHQTGR 205

A0A091T7N1 ITNLVKDMDPQTVMLLASFVFFK--GNWEKPFKPEHTEEKEFFVDAETTVKVPMMHQTGR 205

A0A0A0A7A0 ITNLVKDMDPRTVMLLASFVFFK--GNWEKAFKPEHTEEREFFVDAETTVKVPMMHQTGR 205

A0A091VAK7 ITNLVKDIDPQTVMLLASFVFFK--GNWEKPFKPEHTEEKEFYVDAETTVKVPMMHQTGR 205

A0A093P9B6 ITDLVKDMDPQTVMLLASFVFFK--GNWEKPFKPEHTEEREFFVDAETTVKVPMMHQTGR 205

K7FR61 IVELVKDLDPETAMILVSYIFFK--GKWEKPFQPEHTTERDFFVDEKTTVKVPMMHRMGM 225

M7B3F1 IVELVKGLDPETAMILVSYIFFK--GKWEKPFMPEDTKERDFFVDEETIVKVPMMRRMGM 204

K7FUR6 IPKLVGHLDPNTIMVLVNYMYFK--AFWENPFDPFHTHKEDFLVDEKTSVKVNMMCRDTN 223

Q8JIA6 IVDLVKNLGPDTVMVLVNYILLK--AYWEHPFNYEATREEDFFVDGKTSVKVDMMNPDSR 217

A0A093H779 IVKLVEDLDPVTAMVLVNYVFFK--AHWEKPFSTLYTKREDFFVDKKTSVKVDMMYRKGY 202

G1NKH1 IVKLVENLDPLTTMVLVNYVFLK-EAHWQKPFSDSYTKKEDFFVDKKTSVKVDMMYRKGY 211

U3JIK3 IVKLVENLDPLTAMVLVNYVFFK-EAHWEKPFSTAQTKQEDFFVDQKTSVKVDMMYRKGY 215

A0A091EGC4 IVKLVENLDPLTAMVLVNYVFFK--AHWEKPFSTSQTKQEDFFVDQKTSLQVDMMYRKGY 202

A0A093PHW0 IVKLVENLDPLTAMVLVNYVFLR--AHWEKPFSTLYTRQEDFFVDQKTSVKVDMMYRKGH 202

A0A091NZH9 IVKLVENLDPLTAMVLVNYVFFK--AHWKKPFSTSYTIQEDFFVDEQTPVKVDMMYRKGY 194

R0JRB7 IFKLVENLDPLTAMVLVNYVFFK--AHWEKPFSALYTQQEDFFVDEKTTVKVDMMYRKGY 228

A0A091KRT6 IVKLVESLDPLTAMILVNYVFFK--AHWEKPFSTSYTKQEDFFVDQETSVKVDMMYRKGY 202

A0A091FX98 IVKLVENIDPLTAMVLVNYVFFK--AHWEKPFSTSYTKQDDFFVDQKISVKVDMMYRKGY 202

A0A091LT78 IVKLVENLDPLTAMVLVNYVFFK--AHWKKPFSTSYTKQEDFFVDQKTSVKVDMMYRKGY 199

A0A091QJI6 IVKLVENLDPLTAMVLVNYVFFK--AHWEKPFSTSYTKREDFFVDQKTSVKVDMMYRKGY 197

A0A093H5C6 IVKLVENIDPLTALVLVNYVFFK--AHWEKPFSTLYTKQEDFFVDQETSVKVDMMYRKGY 202

A0A091RAD2 IVKLVENLDPLTAMVLVNYVFFK--ADWEKPFSTSYTKKEDFFVDQTKSVKVDMMYRKGY 195

A0A091KQF0 IVKLVQNLDPLTALVLVNYVFFK--AHWEKPFSTSHTKQEDFFVDQKTSVKVDMMYRKGY 202

A0A093IZH1 IFKLVENLDPLTAMVLVNYVFFK--AHWEKPFSTSYTKQEDFFVDQNTSVKVDMMYRKGY 202

A0A091V9J5 IVKLVENLDPLTAMVLVNYVFFK--GHWEKPFSTSYTKQEDFFVDQKTSVKVDMMYRKGY 205

K7FUQ7 IANLVQDLDPLTVMVLINYIFFE--AHWEKPFDSSYTKEEDFFVDRKTSVKVNMMYRKGV 208

M7B3E7 IVDLVQDLDPLTVMILINYVFFK--ADWEKPFNYFYTKEEDFFVDGKTSVKVNMMYRKGS 235

K7FUC4 IAHLVQGLDPLTVMVLINYIYFK--AYWEHPFSDMLTHEADFFVDGKTSVKVNMMTRDGR 220

K7FSH8 IANLVKNLDPLTVIVLVNYIFFK--AHWQNPFNNLTTKEDDFFVDAKTSVKVKMMNREEE 237

M7B5J6 IANLVKDLDPLTVMVLVNYIFFK--ARWENPFSNLNTQEDDFFVDAKTSVKVNMMNRDKD 235

S7ME51 IDNLVSRLSPDTVMVLVNYIYFKGR--WENPFGRYVTKPQDFHVDENTVVKVPMMFQDQY 220

M3XVL7 IVDLVSHLSTDITMVLVNYIYFKGKTLWEKPFVPSMTTTQDFHVDENTVVKVPMMLQDTQ 232

K9IXP7 IVDLVKSLDTRTVMILVNYIFFKAK--WMTPFDPNDTVQSKFYLSKRRSVQVPMMSLEDL 222

A0A0D9REC0 ITDLIEDLDSQTVMVLVNYIFFKAK--WKMPFDPHDTHDSRFYWSKRRWVKVPMMSLQHV 226

K7A8Q9 ITDLIKDLDSQTMMVLVNYIFFKAK--WEMPFDPQDTHQSRFYLSEKKWVMVPMMSLHHL 226

Q5R536 ITDLIKDLDSQTMMVLVNYIFFKAK--WKMPFDPQDTHQSRFYLSKKKWVMVPMMSLHHL 226

A2I7N2 IEELFKDLSPRTELVLVNYVYFKAQ--WKTRFDPKHTEKTEFHVSDNKTVEVPMMTLD-L 222

F1SCC6 IVDLFKQLSPDTVLVLVNCIYFKAK--WKTPFDPSFTTEADFHVSKNRTVRVPMMGISGR 226

F6SWD3 IVELVKDLDETTAMVLVNYIFFKAK--WKTPFDPRDTQEKDFFVDDTTIVTVPMMTQKKA 206

F7CZD2 IVDLVRELDRNSVMVLVNYIYFKAK--WQKPFDAKQTAEEDFFVNEKTVVSVPMMKQKDV 226

A0A091CQE0 IVDLVKGLDEDTVLALVNYIFFRGQ--WEKPFEAEYTSEENFHVNEETTVKVPMMKRLGI 222

G5B496 IVDLVKVLDKDTVLALVNYIFFRGK--WEKPFEAEHTSEEDFHVNEETTVKVPMMKRLGM 217

H0WJ03 IVDLVKELDKDTALALVNYIFFKGK--WEKPFEAELTTEEDFHVDETTTVKVPMMNRLGM 223

U3FMP8 IVDLVKELDEDTAFALVNYIFFKGK--WERPFEVKNTEEEDFHIDQVTSVKVPMMRRLGM 225

1qlp IVDLVKELDRDTVFALVNYIFFKGK--WERPFEVKDTEEEDFHVDQVTTVKVPMMKRLGM 226

G1S644 IVDSVKELDRDTVFALVNYIFFKGK--WERPFEVKDTKEEDFHVDQATTVKVPMMRRLGM 225

P01010 VVDLVKELDRDTVFALVNYIFFKGK--WERPFEVEATEEEDFHVDQATTVKVPMMRRLGM 225

F7CYP1 IVDLVKDLDKDTVLALVNYIFFKGK--WEKPFEPESTTEQDFHVDEKTTVRVPMMHRLSS 228

B5BV12 IVDLVKDLEKDTVLALVNYIFFKGK--WEKPFEPESTTEQDFHVDEKTTVRVPMMHRLSS 228

B5BV05 IVDLVKDLDKDTVLALVNYIFFKGT--WEKPFEPEYTTEQDFHVDEKTTVRVPMMNHLSS 228

L8YAZ3 IVDLVKELNEDAVLALVNYIFFKGK--WQKPFDAEHTTEGDFHVDEATTVKVPMMKRLGM 225

G3TDW3 IVDVVKYLDDNTVLALVNYIFFKGK--WKKPFEEEKTQDGDFHVDEATTVTVPMMNRLGM 226

G1PM73 IVDLVQELSQDTVLALVNYIFFKGK--WVEPFNASHTTEKDFHVDEATTVKVPMMKRQDR 222

L5LNJ6 IVDLVKELDQDTVLALVNYIFFKGK--WVKPFDASETTEEDFHVDEATTVKVPMMKRTGM 224

L5JP19 ITDLVKDLNEDTVFALVNFIFFKGK--WEKPFEVKQTTEEDFHVDQDTTVKVPMMNRLGM 220

M3WCX1 IVDLVQDLDKDTVFALVNYIFFKGK--WEKPFEPEHTTEEDFHVDEDTTVRVPMMSRLGM 226

F1PCE5 IVDLVKDLDEDTVFALVNYIFFKGK--WEKPFEVEHTTEEDFHVDEHTTVKVPMMSRLGM 224

M3XVV7 IVDLVKDLDKDTAFALVNYIFFKGK--WEKPFEAEHTTVEDFHVDEHTTVKVPMMSRLGM 206

D2HEM3 IVDLVQDLDKDTVFALVNYIFFKGK--WEKPFEVEHTSVEDFHVDEHTTVQVPMMSRLGM 228

P50447 IVDLVDELDKDTVFALVNYIFFKGK--WEKPFEVEQTTEEDFHVDEETTVKVPMMNRLGM 226

W5PZS7 IVDLVKDLDQDTVFALVNYISFKGK--WEKPFEVEHTTERDFHVNEQTTVKVPMMNRLGM 221

P34955 IVELVKVLDPNTVFALVNYISFKGK--WEKPFEMKHTTERDFHVDEQTTVKVPMMNRLGM 221

P23035 IVDLVQELDARTLLALVNYVFFKGK--WEKPFEPENTKEEDFHVNATTTVRVPMMSRLGR 222

Q07298 IVDLVQELDARTLLALVNYVFFKGK--WEKPFEPENTKEEDFHVDATTTVRVPMMSRLGM 222

O62663 IVDLVQELDARTLLALVNYVFFKGK--WEKPFEPENTKEEDFHVDATTTVRVPMMSRLGM 222

O54761 IVDLVKDLNRDSVLALVNYIFFKGK--WEKPFEVDHTKEEDFHVDQVTTVRVPMMNRMGM 222

O54763 IVDAVKTLDKNTVFALVNYIFFKGK--WEKPFEVEHTTEGDFHVDQATTVKVPMMNRLGR 222

Q76HP1 IVELVKELDRDTVLALVNYIFFKGK--WLKPFNVKSNREEDFHVDEATTVRVPMMYRVGM 222

Q76HP0 IVELVKELDRDTVLALVNYIFFKGK--WEEPFNEEDTKEEDFHVDEATTVRVPMMNRLGM 222

Q76HN9 IVELVKELDRDTVLALVNYIFFKGK--WLKPFNEEHTREEDFHVDEATTVRVPMMNREGR 222

O54760 IVELLKELDRDTVLALVNYIFFKGK--WKQPFNEEQTREKDFHVDEATTVRVPMMNRLGM 222

Q64118 IVDLVKDLEIDTVLALVNYIFFRGK--WEKPFDPELTEEADFHVDKSTTVKVPMMNRMGM 216

P97277 IVDLVKDLDKDTVLALVNYIFFKGK--WKKPFDADNTEEADFHVDKTTTVKVPMMSRLGM 222

G3I296 IVDLVKDLDKDTVLAMVNYIFFKGK--WKKPFRAENTEEADFHVDQSTTVKVPMMKRLGM 222

P17475 IVDLMKQLDEDTVFALVNYIFFKGK--WKRPFNPEHTRDADFHVDKSTTVKVPMMNRLGM 221

Q63969 IVDAVKDLDEDTVFALANYIFFQGK--WKTPFDPEHTTEADFHVNESTTVRVPMMNLMRM 221

P07758 IAEAVKKLDQDTVFALANYILFKGK--WKKPFDPENTEEAEFHVDESTTVKVPMMTLSGM 221

Q00898 IVEAVKKLEQDTVFVLANYILFKGK--WKKPFDPENTKQAEFHVDESTTVKVPMMTLSGM 221

. . : . : . : :. * * * . : **

A0A093GX57 YNTY-RDEKLSCWV--VEIPYKGNVAALFVLPDEGTMKQVEAALLKETVSNWAKSLNNR- 271

A0A099YYG0 YNVY-KDEKLSCWV--VEIPYKGDVSALFVLPDEGAMKQVEDVLLKETVSNWAKSLKSR- 271

G1NKH9 YNIH-RDEKLSCWI--VEIPYTGNAAALFVLPDEGSMNHVEDALLQDTVSNWSQSFEKR- 281

U3IXC3 HNIH-RDEKLSCWV--VEIPYKGDVAALFVLPDEGTMKQVEDALLKDTVSNWSQSLEER- 277

A0A091KYD1 FNIH-RDENLSCWV--VEIPYKGNVTSFFILPDEGTMEQVEDSLLKETVSKWLHSLEKR- 269

A0A091QVX2 FNVH-RDGELSCWV--VEIPYKGNATALFVLPDEESMKQVEDALLRKTVTKWIHSLKKR- 266

A0A091NC43 FNVY-RDDKLSCWI--VEIPYKGNVAALFVLPDEGTMKQVEDALLKETVSNWMQSLKLR- 271

A0A091GSL7 FNIH-RDEKLSCWV--VEIPYKGNVAALFILPDEGKMKQVEDALLKETMSNWMQSFKER- 269

A0A093PGJ8 FNIH-RDENLSCWV--VEIPYTGNVTSLFVLPDEGKMKHVEDALLKETVSNWMESLKIR- 271

A0A091NIR3 FNIH-RDEKLSCWV--VEMPYKGNVTSLFVLPDEGAMKQVEDALLKETVSNWMESLKKR- 271

A0A091G0Q5 FNIH-RDEKFSCWV--VEIPYKGNAAALFILPDEEKMKQVEDALLEETVSNWIQSLEKR- 271

A0A093GVB9 FNVH-RDERLSCWV--VEIPYKGNAAALFVLPDEGAMKQVEDALVKETVSNWTQSLRKR- 269

A0A093CUY6 FNIH-RDEKFSCWV--VEIPYKGNAVALFVLPDEGTMKQVEDALIKETVSNWMQSLEKR- 271

A0A091QM83 FNVH-RDENLSCWV--VEIPYKGTASALFILPDEGTMKQVEDALLEETLSNWMQSLKKR- 271

A0A091PDX7 FNIH-RDEKLSCWI--VEIPYKGNAAALFVLPDEGMMKQVEDALLKETVFNWMQSLEGR- 271

A0A091WBP3 FNIH-RDEKMSCWV--VEIPYKGNAISLFVLPDEGTMKQVEDALLRETVSNWMQSFQKR- 271

A0A091SNX6 FNVH-RDEKLSCWV--VEIPYKGNASALFVLPDEGTMSQVEDVLLKETVSNWMQSLEER- 271

A0A094KEC1 FNIH-RDEQLSCWV--VEIPYKGNVTSLFILPDEGTMKQLEDALLKETVSKWIKSLEKR- 271

A0A0A0ABV8 FNVH-RDEKMSCWV--VEIPYKGNAVALFILPDEGTMKQVEDVLLKETVSNWMQSLKKR- 271

A0A093JEM7 FNIH-RDEKLSCWV--VEIPYKGNATSLFVLPDEGTMKKVEDALLKETVSNWMQSLEKR- 269

A0A093G1U7 FNIH-RDEKLSCWV--VEIPYKGDATSLFVLPDEGTMKQVEDTLLKETVSNWIQSLKKR- 271

A0A091JCG9 FNVH-RDEKLSCWV--VEIPYKGNAAALFVLPDEGTMKQVEDALLKETVFNWMQSFEKR- 271

A0A091U8N8 YNIH-RDEKVSCWV--VEIPYKGNAIALFILPDEGTMNQVEDALVKETVSNWMQSLKKR- 271

A0A091RXN3 FNIH-RDENLSCWV--VEIPYKGNATSLFILPDEGTMQQVEDALLKETVYNWMQSLKKR- 269

A0A093R765 FNIH-RDEKLSCWV--LEMPYKGNAAALFILPDEGTMRQVEDALLKETMSKWMQSLEKR- 271

A0A087VMC1 FNIH-RDEKLSCWV--VEIPYKGNATALFVLPDEGTMKQVEDALLKETVSNWMQSLENR- 271

A0A093F995 FNIY-RDEKLSCWV--VEIPYKGNATSLFVLPDEGTMKQVEDALLKETVSNWMQSLEKR- 271

A0A093DUW7 FNIH-RDDKLSCWV--VEIPYKGNATALFVLPDEGSMNQVEDALLKETVSNWMQSLEKR- 271

A0A091T9D5 FNIH-RDEKLSCWV--VEIPYKGNATSLFVLPDEGTMKQVEDALLKETVSNWMQSLEKR- 271

A0A091LUF5 FNIH-RDEKLSCWV--VEIPYKGNAAALFILPDEGTMKQVEDALLKETVSNWMQSLEQR- 271

A0A091V8N3 FNIH-RDEKLSCWV--VEIPYKGNASALFILPDEGTMKQVEDALLKETVSNWMQSLEKR- 271

A0A091LFJ1 FNIH-RDKKLSCWV--VEIPYKGNATALFVLPDEGTMKQVEDALLKETVSNWMQSLEKR- 271

A0A087R544 FDIH-RDENLSCWV--VEIPYKGNAAALFVLPDEGTMKQVEDALLKETVSNWMESLEKR- 271

A0A091LM39 FNIH-RDEKLSCWV--VEIPYKGNAAALFVLPDEGTMKQVEDALLKETVSNWMQSLEKR- 271

Q66KX6 YNVA-YDDQLGCTV--VLMPYKGNATALFILPDEGKLRQVEEALETPVVKSWRKIFRR-- 277

A0A099Z110 YKSY-FDRKLSCEV--VQIPYTGDAMTLFVLPSRGKMKRLEDALAKDTVSKWEKSLER-- 260

G1NKI3 YDIY-SDKKLSCKV--VRIPYKGNVSALFILPNEGKMKWLEDGLMKDTVSKWEKSLER-- 286

E1C206 YDIY-SDKKLSCKV--VRIPYKGNVSALFILPNEGKLKWLEDGLKKDTVSKWEKSLER-- 284

R0LEI7 YKMY-SDKMLSCKV--VQIPYKGDVAALFVLPNEGKMKWLENALTKETVSKWEKLLER-- 279

A0A091F2Q1 YKAY-SDRKLSCKV--VQIPYKGDVAALFILPNKGKMKQLEHALTKKTVSKWERSLQR-- 260

A0A093IN74 YKTY-SDRKLSCEV--VQIPYKGDVAAFFILPNEGKMKHLEDALTKDTLSKWEKTLKR-- 260

A0A093C116 YKTY-SDRKLSCEV--VQVPYKGEVAALFVLPTEGKMKRLEDALTKDTVSRWEKSLKR-- 260

A0A093PVA9 YKAY-SDRKLSCKV--VQIPYKGDVAALFILPNEGKMKQLERGLTKDTVSKWEKSLQR-- 260

A0A091KNU4 YKTY-SDRKLSCEV--VQIPYKGDVAALFILPRQGKMKQLEDALTKDTVSKWEKSLER-- 259

A0A087VMC2 YKTY-SDRKLSCEV--VQIPYKGDVAALFILPNEGKMKQLEEALTTDIVSKWEKSLER-- 260

A0A091PCC0 YKTY-SDQKLSCKV--VQIPYKGDVAALFILPNEGKMKQLEDALTKDTLSKWEKSLER-- 250

A0A093FFU5 YKTY-SDRKLSCEV--VQIPYKGDVAALFILPNGGKMKQLEDALTKDTVSKWEKSLER-- 253

A0A091XH23 YKTY-SDKKLSCNV--VQIPYKGDVAALFILPNEGKMKQLEDALTKDTVSKWEKSLER-- 260

A0A091T7I8 YKTY-SDRKLSCKV--VQIPYKGDVAALFILPNERKMKQLEDALTKDIVSRWEKSLQR-- 250

A0A093CLJ8 YQTY-SDRKLSCEV--VQLPYKGDVAALFILPNEGKMKQLEDALTKDTLSKWEKSLER-- 260

A0A093KV79 YKTY-SDRKLSCEV--VQIPYKGDAAALFILPSEGKMKQLEDALTKDTVSKWEKSLER-- 260

A0A087R545 YKTY-SDRKLSCKV--VQMPYKGDVAALFVLPNEGKMKQLEDALTKATVSKWEKSLER-- 250

G1KWY8 YKYL-HDDDLFCSV--VEIPYKGGASAFFILPDEGKMQQVEDSLVKGHLYKWAKALEY-- 290

G1NKH5 FDLY-FDEDLPCTV--VRLHYNGSATAFLVLPAEGKMKQLERTLDKERVKKWSDNLFK-- 277

A0A093H648 FDFY-FDEKLPCTV--VRLHYNGSATAFLVLPEKGKMKHLEQMLVKETVHEWSENLFQ-- 261

A0A091PB47 FDFY-FDEDLSCTV--VRLNYNGSATAFLVLPAKGKMKKLEQALDKETIQHWSDHLFQ-- 260

A0A093QJ65 FDFY-FDEELSCTV--VRLHYNGTATAFLVLPAKGKMKQLEQTLVKETIQQWSDHLFQ-- 260

A0A091K7I5 FDFY-FDKELSCTV--VRLHYNGSATAFLVLPAEGKMKQLEQTLVKEIIQEWSDHLFQ-- 260

A0A091K0Z5 FDFY-FDEELSCTV--VRLHYNGSATAFLVLPGKGKMKQLEQTLIKENIQKWSDNLFQ-- 260

R0LHU9 FDLY-FDMELSCTV--VRLHYNGSATAFLVLPAKGKMKQLEQALVKETIREWSDRLLQ-- 278

H0ZQY2 FDFY-FDEELSCTV--VRLHYNGSATAFLVLPAKGKMKQLEQTLDKETIQKWSDHLFQ-- 261

A0A091GLC8 FDFY-FDGDLSCTV--VRLHYNGSATAFLVLPSKGKMKQLEQTLVKETIQKWSDHLFQ-- 260

A0A091HV30 FDLY-FDEELSCTV--VRLHYNGSATAFLVLPAKGKMKQLEQTLVKEIIQKWSDHLFQ-- 260

A0A093RZ50 FDFY-FDEELSCTV--VRLHFNGSATAFLVLPAKGKMKQLEQTLDKETIQKWSDHLFQ-- 260

A0A091SWC2 FDFY-FDEDLSCTV--VRLHYNGSATAFLVLPAKGKMKQLEQTLVKETIQEWSDHLFK-- 260

A0A091QU84 FDFY-FDEELSCTV--VRLHYNGSATAFLVLPAKGKMKQLEQTLVKETIQEWSDNLFQ-- 260

A0A093GS00 FDFH-FDEELSCTV--VRLHYNGSATAFLVLPAKGRMKQLEQTLVKEIIQEWSDHLFQ-- 260

A0A093IEQ9 FDFY-FDEKLSCTV--IQLHYNGSATAFLVLPAKGKMKQLEQTLVKETVQEWSDRLFQ-- 260

A0A091PLB4 FDFY-FDEELSCTV--VRLHYNGSATAFLVLPAKGKMKQLEQTLVKETIQKWSDHLFQ-- 260

A0A093FVA3 FDFY-FDEELSCTV--VRLHYNGSATAFLVLPAKGKMKQLEQSLVKETVQKWSDHLFQ-- 260

A0A091U6W4 FDFY-FDEELSCTV--VRLHYNGSATAFLVLPAKGKMKQLEQTLVKEIIQEWSENLFQ-- 260

A0A091KQX5 FDFY-FDEKLSCTV--VRLHYNGSATAFLVLPAKGKMKELEQTLDKEVIQEWSDRLFQ-- 260

A0A091MK58 FDFY-FDEELSCTV--VRLHYNGSATAFLVLPAKGKMKQLEQTLVKERIQKWSDHLFQ-- 260

A0A091T7N1 FDFY-FDEELSCTV--VRLHYNGSATAFLVLPAEGKMKQLEQTLVKETIQEWSDHLFQ-- 260

A0A0A0A7A0 FDFY-FDEELSCTV--VRLHYNGSATAFLVLPAKGKMKQLEQTLVKETIQEWSDGLFQ-- 260

A0A091VAK7 FDFY-FDEELSCTV--VRLHYNGSATAFLVLPAKGKMKQLEQTLVKETIQKWSDHLFQ-- 260

A0A093P9B6 FDFY-FDEELSCTV--VRLHYNGSATAFLVLPAKGKMKQLEQTLVKETIQEWSDHLFQ-- 260

K7FR61 FDFH-FDAELSCTV--VQLHYNGSATAFFIMPEKGKMKQVEDILQKETVSKWSRSVWR-- 280

M7B3F1 FVHH-FDVELSCTV--VQLYYNGSATAFFILPNKGKMKQVEDALQKETVSRWSTSLHR-- 259

K7FUR6 YESH-YDKQLSCWL--VQIPYSGNAKAIFILPDKGKMKQVEAALSQETVCKWEKLLQK-- 278

Q8JIA6 YNSL-HDKKLSCLVQWVDLPLQRNVAATFILPDEGKMKQVEDALSVEVLTRLEKSLRKER 276

A0A093H779 YRNY-YDEELSCWL--VQIPYNGNAAALLVLPDEGKMKQVEDALLKRTVSRWEKFLLD-- 257

G1NKH1 YRNY-FDEELSCWL--VQIPYNGNAAALFVLPDEGKMKQVEDALLKRTVSKWEKLLQD-- 266

U3JIK3 YRNY-FDEELSCWL--VQIPYNGNVAALFVLPDEGKMKQVEDALLKKTVTTWEKSLQD-- 270

A0A091EGC4 YRNY-FDEELSCWL--VQIPYNGDVVALFVLPDEGKMKQVEDALLKRTVTKWEKSLQD-- 257

A0A093PHW0 YRNY-FDEELSCWL--VQISYKGDVAALFVLPDEGKMKQVEDALLKKTVSKWEKSLQD-- 257

A0A091NZH9 YRNY-FDEELSCWL--VQIPYNGDAAALFVLPDKGKMKQVEDALLKRTVSKWEKFLQD-- 249

R0JRB7 YRNY-FDEVLSCWL--VQIPYNGNVAALFVLPDEGKMKQVEDALLKRTMSKWVKFLQD-- 283

A0A091KRT6 YKTY-FDEELSCWL--VQIPYNGNAAALFVLPDEGKMKQVEDALLKRTVSKWEKFLQD-- 257

A0A091FX98 YRNY-FDEELSCWL--VQIPYNGNAAALFILPDKGKMKQVEDALLKKTVSKWEKSLQD-- 257

A0A091LT78 YRSY-FDEELSCWL--VQIPYNGNAAALFVLPDEGKMKQVEDALLKRTVSKWEKFLQD-- 254

A0A091QJI6 YRNY-FDEDLSCWL--VQIPYNGNAAALFVLPDEGKMKQVEDALLKRTVSKWEKLLQD-- 252

A0A093H5C6 YRNY-FDEELSCWL--VQIPYNGNAAALFVLPDEGKMKQVEDALLKRTVSKWEKFLQD-- 257

A0A091RAD2 YRNY-FDEDLSCWL--VQIPYNGNTAALFVLPDEGKMKQVEDALLKKTVSKWEKSLQD-- 250

A0A091KQF0 YRNY-FDEELYCWL--VQIPYNGNAAALFILPNEGKMKQVEDALLKRTVSKWEKFLQD-- 257

A0A093IZH1 YRNF-FDEELSCWL--VQIPYSGNAAALFILPDKGKMKQVEDALLKRTVSKWEKLLQD-- 257

A0A091V9J5 YRNY-FDEELSCWL--VQIPYNGNAAALFILPDEGKMKQVEDALLKRTVSKWEKFLQD-- 260

K7FUQ7 YKHH-YDKELSCWL--VEIPYHGKAIALFILPDEEKMKVVENALLKKTLFKWKKAVQE-- 263

M7B3E7 YKHH-YDKELSCWL--VQIPYSGKAVALFILPDEGKMKEVEKALMNKTLSKWKKAFKE-- 290

K7FUC4 YNSY-HDKELSCQL--VEIPYKGNATALFILPDEGKMKQVEDALLKETVAKWIKSLKK-- 275

K7FSH8 YKTY-RDEMLACWV--VEIPYKGNAAALFILPDEGKMEQVEEALLKETVSKWAKSLEQ-- 292

M7B5J6 YHTH-RDEELSCWV--VEIPYKGNATALFILPDEGKMKQVEEALLKETVSKWTTSLQE-- 290

S7ME51 KQWYLHDKHVPCSV--LRMDYKGGARALFILPDPGKMAQVEEVLTPEMLRRWSSLLEKRS 278

M3XVL7 HHWYLHDRFLPCSV--LRMDYQGNMVAFFILPDQGKMAHVEEVLTPEMLTRWNNLLQKRK 290

K9IXP7 YTPYFRDKELSCTV--VELRYTSNDSMLLILPDQGKMKEVEAALLPETLRRWRGSLQMR- 279

A0A0D9REC0 TTPYFRDEELSCTV--VELKYIGNASALFILPDQDKMEEVEAMLLPETLKRWKDSLEFR- 283

K7A8Q9 TIPYFRDEELSCTV--VELKYTGNASALFILPDQDKMEEVEAMLLPETLKRWRDSLEFR- 283

Q5R536 TTPYFRDEELSCTV--VELKYTGNASALFILPDQDKMEEVEAMLLPETLKRWRDSLEFR- 283

A2I7N2 ETPYFRDEELGCTL--VELTYTSNDSALFILPDKGKMQDLEAKLTPEMLTRWRNSLQPR- 279

F1SCC6 TLPYFRDEELACTV--VELPYTSNDSALFILPDDGRMAAVEAQLLPETLRRWRDSLHQR- 283

F6SWD3 SHRFLHDEELACLV--LEMNYKGNASALFILPDEGKMKLVEEALTRETITRWNNQLRRR- 263

F7CZD2 YHYYFYDKWLSCSV--LRMDYKGNATAFFILPDVGKMREVENSLQPGMLMRWDRFFKRSS 284

A0A091CQE0 FRVF-HCTTIQSWV--LLMDYQGNVTALFLLPDKGKLQHLEETLTEELIDKFLSK--TQK 277

G5B496 FRVF-HCSTIQSWV--LLMDYQGNITALFLLPDEGKLQHLEKTLTKELISKFLSK--TEK 272

H0WJ03 FNTH-YCEMLSSWV--LMMDYVGNATAIFFLPDEGKLQHLEDKLTKEVVTKFLDN--RQR 278

U3FMP8 FNIH-HCDKLEGWV--LLMKYLGNATALFFLPDDGKLQHLENELTHDVISKSLEA--ETR 280

1qlp FNIQ-HCKKLSSWV--LLMKYLGNATAIFFLPDEGKLQHLENELTHDIITKFLEN--EDR 281

G1S644 FNIH-HCEKLSSWV--LLMKYLGNATAIFFLPDEGKLQHLENELTHDIITKFLEN--ENR 280

P01010 FNIY-HCEKLSSWV--LLMKYLGNATAIFFLPDEGKLQHLENELTHDIITKFLEN--ENR 280

F7CYP1 FDVQ-YSDTLSSWV--LLLDYAGNATAFFILPDQGKLRHLEDTLTKGILATRFRTSSPPP 285

B5BV12 FDVQ-YSDALSSWV--LLLDYAGNATAFFILPDQGKLQHLEDTLTKGILARFLGN--RHS 283

B5BV05 FDVQ-YSVALSSWV--LLLDYAGNATAFFILPDQGKLQHLEDTLTKGILARFLGN--RHS 283

L8YAZ3 FEIQ-HCDTLSSWV--LLMDYVGNATAFFIMPDEGKMQHMERVMNKQILSKFLEN--RHT 280

G3TDW3 FDLH-YHEELSSWV--LLMDYVGNATAIFILPKPGELQQLEDTLTKELLAQWLTD--RQR 281

G1PM73 YNLH-YCDKLSSQV--LLMDYVGNATAFFILPDEGKLRHMENLLTKEDLSKALGE--RHY 277

L5LNJ6 YKLH-QCHKLSSWV--LLMDYVGNATAFFILPNEGKMRHMEDLLTKEDLSMALKN--RDK 279

L5JP19 FDLH-RCDTLSSWV--LLMDYVGNATAVFLLPDEGKLQHAEDLLTKQVLSKFLEK--RYV 275

M3WCX1 FDVH-YCDMLSSWS--LLMDYVGNATAFFVLPDQGKMQRLEEMLNRDILAKALEK--RHS 281

F1PCE5 FDIQ-HCATLSSWV--LLMEYVGNATAFFILPDEGKMQHLESKLTKEVLAKFLEK--RYA 279

M3XVV7 FDVH-HCNKLSSWV--LLMDYVGNATAIFILPDQGKMHHLEDTLTKEVLAKFLEN--RHT 261

D2HEM3 FDVH-HCDKLSSWV--LLMDYVGNATAIFLLPDQGKMQQLEDTLTKEVLAKFLEH--RHT 283

P50447 FDLH-HCDKLSSWV--LLMDYVATATAFFILPDQGKLHQLEDMLTKEIRAKFLEK--RYP 281

W5PZS7 FDLH-YCDKLASWV--LLLDYVGNVTACFILPDLGKLQQLEDKLNNELLAKFLEK--KYA 276

P34955 FDLH-YCDKLASWV--LLLDYVGNVTACFILPDLGKLQQLEDKLNNELLAKFLEK--KYA 276

P23035 YDLF-HCSTLASTV--LRMDYKGNATALFLLPDEGKLQHLEDTLTTELITKFLAK--SSL 277

Q07298 YVKF-HCSTLASTV--LRMDYKGNATALFLLPDEGKLQHLEDTLTTELIAKFLAK--SSF 277

O62663 YVTL-HCSTLASTV--VLMDYKGNATALFLLPDEGKLQHLEDTLTTELIAKFLAK--SSL 277

O54761 FEVH-YCSTLASWV--LQMDYLGNATAIFLLPDEGKLQHLEDTITKEILAKFLKN--RES 277

O54763 FDLL-YCTTLASWV--LQMDYLGNATAIFLLPDEGKLQHLEDTITKEILSKFLKN--RHT 277

Q76HP1 FPVD-YCRTLASRM--LQMDYLGNATAIFLLPDKGKMQHLEDTISTEILSKFLKD--RQT 277

Q76HP0 FHLH-RCSTLASWV--LQMDYLGNATAIFLLPDKGKMQHLEDTVTMEILSKFLKN--RET 277

Q76HN9 FHLH-HCSTLASWV--LQMDYLGNATAIFLLPDEGKMQHLEDTVTTEILSKFLKN--RQT 277

O54760 FHLH-HCSTLASWV--LQMDYLGNATAIFLLPDKGKMRHLEDTVTTEILTKFLKN--RET 277

Q64118 FDVH-YCDTLSSWV--LLMDYLGNATAIFILPDEGKMQHLEQTLTKEHIYKFLQN--RHT 271

P97277 FDVH-YVSTLSSWV--LLMDYLGNATAIFILPDDGKMQHLEQTLNKEIIGKFLKD--RHT 277

G3I296 FDVH-HCSTLSSWV--LLMDYLGNATAIFILPDEGKMQHLEQTLNKEIISKFLEN--RHT 277

P17475 FDMH-YCSTLSSWV--LMMDYLGNATAIFLLPDDGKMQHLEQTLTKDLISRFLLN--RQT 276

Q63969 LDVH-YCSTLSSWV--LMMDYLGNATAVFLLPDDGKMQHLEQTLNKELISKFLLN--RHR 276

P07758 LHVH-HCSTLSSWV--LLMDYAGNATAVFLLPDDGKMQHLEQTLSKELISKFLLN--RRR 276

Q00898 LDVH-HCSTLSSWV--LLMDYAGNATAVFLLPDDGKMQHLEQTLNKELISKFLLN--RRR 276

. : : :.:* : * :

A0A093GX57 ----QIYLHLPKFSISGSYDVKHLFQEMGVTEVFSDQADLSGMTE-NTRLKVSKAIHKAK 326

A0A099YYG0 ----KIHLYIPKFSISGCYDVKNLFKKMGVTEVFGDQANLSGMTE-NTSLKVSKAMHKAT 326

G1NKH9 ----SVDLYLPKLSISGSYDVKRLFLKMGVTDMFSNNADFSGVAK-NTHLKVSKAIHKAK 336

U3IXC3 ---RPIELYLPKFSISGSYDVKSIFIKMGVTDVFSNNADLSGVAE-GDRLMVSKAIHKAV 333

A0A091KYD1 ----NIYLDLPKFSISGTYDVKGLFEKLGVTEVFSDQADLSGVVE-KPLLKVSKAIHEAT 324

A0A091QVX2 ----KIFLDLPKFSISGTYDVKSLFEKMGVTEVFSDHADLSGVAE-DTPLKVSKTIHKAL 321

A0A091NC43 ----EIYLDLPKFSISGSYDVKSLFEKMGITEVFSDQADLSGVAE-KRLLKVTKATHKAM 326

A0A091GSL7 ----KIYLHLPKFSISALYDVKSLFEKMGVTEVFSDLADLSGVAE-NTLLKVSAAIHKAM 324

A0A093PGJ8 ----EVYLDLPKFSVSGSYDVKSLFEKMGVTEVFSNQADLSGVAE-ETLLKVSKAIHKAT 326

A0A091NIR3 ----KIYLDLPKFSISGSYDVKNLFEKMGVTEVFSDQADLSGVAE-NSLLKVSKAIHKAA 326

A0A091G0Q5 ----KIYLDLPRFSISGSYDVKRLFKKMGVTELFSDQADLSGVAE-KTLLKVSKAIHKAT 326

A0A093GVB9 ----EIYLDLPKFSISGSYDVKSMFEKMGVTEVFSNQADLSGVSE-NIHLKVSRALHKAV 324

A0A093CUY6 ----KIYLDLPKFSISAFYDIKSLLEKMGVTEVFSDQADLSGVAG-KTGLTVSKAIHKAV 326

A0A091QM83 ----KIYLDLPKFSISGSYNVKSLFAKMGVTEVFSDQADLSGVAE-NTLLKVSKAIHKAM 326

A0A091PDX7 ----NIYLDLPKFSMSGSYDVKGLFQKMGVTEAFSNQADFSGVVE-KKPLSVSKAIHKAV 326

A0A091WBP3 ----KIYLDLPKFSISSSYDVKSLFEKMGVTEVFSHQADLSGMAE-KALLKVSKAIHKAV 326

A0A091SNX6 ----NIYLDLPKFSISSSYDVKSLFQKMGVTEVFSGQADLSGIAE-NALLKVSRAIHKAT 326

A0A094KEC1 ----KIYLDLPKFSVSGSYDVKNLFEKMGVTEVFSDQADLSGVAE-KTLLKVSKAIHKAT 326

A0A0A0ABV8 ----KIYLDLPKFSISGSYDIKSLFEKMGVTEVFSDQADLSGVAE-KTRLKVSKAIHKAV 326

A0A093JEM7 ----KIYLDLPKFSVSSCYDVKSLFEKMGVTEVFSDQSDLSGVAE-NSLLKVSKAIHKAM 324

A0A093G1U7 ----KIYLDLPKFSISGCYDVKSLFKKMGVTEVFSDQADLSGVSE-ETRLKVSRAIHKAT 326

A0A091JCG9 ----KIYLDLPKFSISGSYDVKSLFEKMGVTEVFSDDADLSGIAE-KTLLKVSKAIHKAV 326

A0A091U8N8 ----KIYLDLPKFSISGSYDVKSLFEKIDVTEVFSDEADLSGVAE-NARLKVSKAIHKAM 326

A0A091RXN3 ----KIYLDLPKFSISSAYDVKSLFEKMGVTELFSDQADLSGVAE-KTLLKVSKAIHKAM 324

A0A093R765 ----KIYLYLPKFSISTSYDVKSLFEKMGVTEVFSDQADLSGVAE-NTLLKVSKAIHKAM 326

A0A087VMC1 ----KIYLDLPKFSISGSYDIKSLFEKMGVTELFSDAADLSGMAE-NTLLKVSKAIHKAT 326

A0A093F995 ----KIYLDLPKFSISGSYDVKSLFEKMGVTELFSGKADLSGVAE-KSLLKVSKAIHKAT 326

A0A093DUW7 ----KIYLDLPKFSISGSYDVKNLFEKMGVTEVFSDQADLSGVAE-KTLLKVSKAIHKAM 326

A0A091T9D5 ----KIYLYLPKFSISGCYDVKSLFKKMGVTEVFSDQADLSGVAE-KTLLKVSRAIHKAT 326

A0A091LUF5 ----KIYLDLPKFSISSSYDVKSLFEKMGVTEVFSDQADLSGVAE-NTLLKISKAIHKAM 326

A0A091V8N3 ----KIYLDLPKFSISGSYDVKSLFEKMGVTEVFSNQADLSGVAE-KTPLKASKAIHKAM 326

A0A091LFJ1 ----KIYLDLPKFSISGSYDVKSLFEKMGVTEVFSNQADLSGVAE-KTLLKVSKAIHKAM 326

A0A087R544 ----KIYLDLPKFSISGSYDVKSLFQKMGVTEVFSDQADLSGVAE-KTLLKVSKAIHKAM 326

A0A091LM39 ----KIYLDLPKFSISGSYDVKSLFEKMGVTEVFSDQADLSGVAE-KTLLKVSKAIHKAK 326

Q66KX6 ---RSVNLTLPKFSISATLDLVKELTKLGVTDVFSDGSNLSGITE-APPLRVSKAVHKAL 333

A0A099Z110 ---RRIEVHIPKLSIYGTYDLKKMFMNLGVTDVFSDHADLSGITG-EPDIKVSKAAHKAL 316

G1NKI3 ---RRMEVHIPKVSISGTYDLKKIVMNLGVTDVFSDQADLSGITG-KSDLKVSRAIHKAL 342

E1C206 ---RRMEVHIPKVSISGTYDLKKMAMNLGVTDVFSDQADLSGITG-KSDLKVSRAIHKGL 340

R0LEI7 ---RRVEVYIPKLSISGTYDLKKMFMNLGVTDVFSDWADLSGITG-KPDLKISRAMHKSL 335

A0A091F2Q1 ---WRMELHIPKLSISGTYDLKKILMNLGVTDVFSDQADLSGITG-NPDVKVSKATHKAL 316

A0A093IN74 ---RRIEVYIPKLSILGTYDLKKMLMNLGVTDVFSDLADLSGITE-KPDVKVPKAAHKAL 316

A0A093C116 ---RRIELYIPKLSISGTYDLKKMFINLGATDVFSDRADLSGITE-KPDVKVSKAAHKAL 316

A0A093PVA9 ---RRIEVHIPKLSVLGTYDLKKILMNLGVTDVFSDQADLSGITG-NLDVKVSKAAHKAL 316

A0A091KNU4 ---RRMEVHIPKLSISGTYDLKKMLMNLGVTDVFSDRADLSAITG-KPQVMVSKAAHKAL 315

A0A087VMC2 ---RRIELYIPKLSISGTYDLKKMFMTLGVTDVFSDQADLSGITG-RPDLKVSKVTHKAL 316

A0A091PCC0 ---RRIEVYIPKLSISGTYDLKKMFMNLGVTDVFSDRADLSGITG-RLDVKVSKAAHKAL 306

A0A093FFU5 ---RRIQVCIPKLSISGTYDLKKMFINLGVTDVFSDWADLSGITG-KPEVKVSKAAHKAL 309

A0A091XH23 ---QRIEVYIPKLSISGTYDLKKLFMNLGVTDVFSDRADLSGITG-KPDVKVSKAAHKAL 316

A0A091T7I8 ---RRIELYIPKLSISSTYDLKKLFMNLGVTDVFSDRADLSGITG-KPDVKVSKAAHKAL 306

A0A093CLJ8 ---RRIEVYIPKLSISGTYDLKKMFMNLGVTDVFSDRADLSGITG-KHDLKVSKAVHKAL 316

A0A093KV79 ---RRIEVYIPKPSISGSYDLKKMFMNLGVTDVFSDQADLSGITG-KPDVKVSKAAHKAL 316

A0A087R545 ---RRIEVYIPKLSISGSYDLKKIFMNLGVTEVFSDRADLSGITG-KPDVKVSKAAHKAL 306

G1KWY8 ---KQLDLYIPKFSLSKSYDVKALFQRLGVTHVFDGNADLSGIVE-EGNLQVSKAIHKAV 346

G1NKH5 ---SKIQLYFPKFSISGSYEITNILNKMGIVDVFTNQADLSGITG-VPELKVSKVVHKAA 333

A0A093H648 ---RMISLYLPKFSISGSYEVKHTLNKMGIIDVFTDQADLSGITG-APELKVSKVIHKTA 317

A0A091PB47 ---SRMSLYLPKFSVSGRYEISDILSKMGMVDVFTNQADLSGITG-TQDLKVSRAVHQAA 316

A0A093QJ65 ---RLVNLYFPKFSISGNYEIKNILSKMGIVDVFTDQADLSGITG-TLELKLSKVVHKAA 316

A0A091K7I5 ---RLVSLYFPKFSISGSYEITKTLSKMGIVDVFTDHADLSGITG-TSELKVSKVVHKAA 316

A0A091K0Z5 ---SMISLYFPKFSISGSYEITNTLSKMGIVDVFTDQADLSGIAG-APELKVSKVVHKAA 316

R0LHU9 ---SKMRLYFPKFSISGSYEITNILSKMGIVDVFTDRADLSGITG-APELKVSKVVHKAA 334

H0ZQY2 ---RFMNLYFPKFSISGSYEISNTLRKMGIVDVFTSQADLSGITG-SPDLKVSKVVHKAS 317

A0A091GLC8 ---SLVSLYFPKFSISESYEIKNTLSKMGIVDVFTDQADLSGITG-ALELKVSKVVHKAA 316

A0A091HV30 ---SLVSLYFPKFSISGSYEITNTLSKMGIVDVFTDQADLSGITG-TPELKVSKVVHKAA 316

A0A093RZ50 ---SFMDLYFPKFSISGSYEISNTLRKMGIVDVFTNQADLSGITG-TPELKVSKVVHEAS 316

A0A091SWC2 ---RLLSLYFPKFSISGSYEITNMLSRMGIVDVFTDQADLSGITG-TPELKVSQVVHKAS 316

A0A091QU84 ---SPMSLYFPKFSISGSYEITNILSKMGVVDLFTNQADLSGITG-TPELKVSKAVHKAA 316

A0A093GS00 ---RLVRLYFPKFSISGSYEITNTLSKMGIVDVFTDQADLSGITG-TPDLKVSKVVHKAS 316

A0A093IEQ9 ---SLVSLYFPKFSISGSYEITNTLRRMGIVDVFTDRADLSGITG-TPELKVSKVFHKAA 316

A0A091PLB4 ---SLISLYFPKFSISGSYEITNTLSKMGIVDVFTNQADLSGITG-TSELKVSKVVHKAA 316

A0A093FVA3 ---SLMRLYFPKFSIFGSYEITNTLSKMGIVDVFSDQADLSGITG-TPELKVSKVIHKAA 316

A0A091U6W4 ---SLVSLYFPKFSISGSYDIMNTLSKMGIVDVFTNQADLSGITG-VPELKVSKVVHKAA 316

A0A091KQX5 ---SLMILYFPKFSISGSYEITNTLSKMGIVDVFTDQADLSGITG-APELKVSKVVHKAA 316

A0A091MK58 ---SLVSLYFPKFSISGSYEIMNILRKMGIVDVFTDQADLSGITG-APELKVSKVVHKAA 316

A0A091T7N1 ---RLVSLYFPKFSISGSYEITNTLSKMGIVDVFTDQADLSGITG-APELKVSKVVHKAA 316

A0A0A0A7A0 ---SLMSLYFPKFSISGSYEITNTLSKMGIVDVFTDQADLSGITG-APELKVSKVVHKAA 316

A0A091VAK7 ---SLVSLCFPKFSISGSYEITNTLSKMGIVDVFTDQADLSGITG-APELKVSKVVHKAA 316

A0A093P9B6 ---SLMSLYFPKFSISGSYEITNTLSKMGIVDVFTDQADLSGITG-TPELKVSKVFHKAA 316

K7FR61 ---SSVNLYIPKFSISATYDLKNHLINMGITDVFTDQADLSGITG-ERELKVSRVVHKAV 336

M7B3F1 ---SSAILYIPKFSISATYDLKDHLIKMGVTDVFTKQADLSGITG-KPELQVSRVVHKAV 315

K7FUR6 ---RSIDLYMPKFSISGFYDVKFLFEKMGITDVFSGHADLSGITA-TSNLRVSKAIHKAV 334

Q8JIA6 ---SRIHLSIPRFSISGTYDVKEIFRRMGVTEVFTNQADLSGITG-SPELKVSRAVHRAH 332

A0A093H779 ---RKIHLHIPRFSISGTYDVKKIVKQMGMIDLFTEQADLSGITE-EPGLMVSKVIHKAL 313

G1NKH1 ---RKIHLHIPKLSISGTYDVKKIVREVGIIDLFTEQADLSGITE-DPGLMVSKVIHRAV 322

U3JIK3 ---RKIHLHIPKFSISGTYDVKNIVQQMGMVNLFTEQADLSGITE-EPGLMVSKVIHRAM 326

A0A091EGC4 ---RKIHLHIPKFSISGTYDVKKIIKQMGMVDLFTEQADLSGITE-EPGLMVSKVVHRAM 313

A0A093PHW0 ---RKIHLHIPKFSISGTYDVKRIVKQMGMIDLFTDQADLSGITE-EPGLKVSKVIHKAV 313

A0A091NZH9 ---RKIHLHIPKFSISGTYDVKRIVKQMGMIDVFTEQADLSGITE-EPGLMVSKVIHKAM 305

R0JRB7 ---RKIHLHIPKFSISGTYDVKKIVKQMGMTDIFTEHADLSGITE-EPGLMLSKVIHRAV 339

A0A091KRT6 ---RKIHLHIPKFSISGTYDVKRIVKQVGMIDLFTEQADLSGITE-EPGLMVSKVIHRAV 313

A0A091FX98 ---RKIYLHIPKFSISGTYDVKKIVKQVGMIDLFTEQADLSGITE-KPGLTVSKVIHRAV 313

A0A091LT78 ---RKIHLHIPKFSISGTYDVKRIVKQVGMIDLFTEQADLSGITE-EPGLMVSKVIHRAM 310

A0A091QJI6 ---RKIHLHIPKFSISGTYDVKELVKQVGIIDLFTEQADLSGITE-EPGLMVSKVIHRAM 308

A0A093H5C6 ---RKIHLHIPKFSISGTYDVKKIVKQVGMIDLFTEKANLSGITE-EPGLMVSKVIHRAV 313

A0A091RAD2 ---RKIHLHIPKISISGTYDVKRIVKEVGMIDLFTEQADLSGITE-EPGLMVSKVIHRAV 306

A0A091KQF0 ---RKIHLHIPKFSISGTYDVKRIVKQAGMIDLFTEQADLSGITE-EPGLMLSKVIHRAV 313

A0A093IZH1 ---RKIHLHIPKFSISGTYDVKRIVKQVGIIDLFTEQADLSGITE-EPGLMVSKVIHRAV 313

A0A091V9J5 ---RKIHLHIPKFSISGTYDVKRIVKQVGMIDLFTEQADLSGITE-EPGLMVSKVIHRAV 316

K7FUQ7 ---RTIYLHIPKFSISGTYDVKEIFQKMGVTDVFTDQADLSGITG-ISNLKVSKVIHKAL 319

M7B3E7 ---RTIYLHIPKFSISGTYDIKEIFQKMGVIDVFTDQADLSGITE-KSELKVSKVIHKAV 346

K7FUC4 ---GRIELYIPKFSISAFYDIEEIFKRMGVTAVFEDHADLSGITE-DHNLKISKAVHKAL 331

K7FSH8 ---REIDLYIPVFSVSGTYDVKELFQKMGVIDVFTDKADLSGITG-ERNLKVSKAVHKAL 348

M7B5J6 ---REINLYIPAFSVSGTYDVKELFQKMGVIDVFTDEADLSGITE-TAKLKVSKVVHKAL 346

S7ME51 YS-RKVWLYLPKFSISGSYSLDQLLPRLGIQDLFSRQANLSGIST-QENLMVSKSVHKAV 336

M3XVL7 -----LELHFPKFSISGSYQLDQILPKMGFMNLFSKEVDLSGITE-ERRLRVSKSFHKAI 344

K9IXP7 ---SI-ELFLPRFSISSNYNLEDILPQLGMRQVFSTKADLSGITG-AKNLAVTQVIHRTL 334

A0A0D9REC0 ---HIDELYLPKFSISRDYDLEDVLLQLGIKEVFTSKADLSGITG-ARNIAVSQVVHKAV 339

K7A8Q9 ---EIGELYLPKFSISRDYNLKDILLQLGIEEAFTSKADLSGITG-ARNLVVSQVVHKAV 339

Q5R536 ---RIDELYLPKFSISRAFNLENILLQLGIVEAFTSKADLSGITG-ARNLVVSQVVHKAV 339

A2I7N2 ---RIHELYLPKFSIKSNYELNDTLSQMGIKKIFT-DADLSGITG-TADLVVSQVVHGAA 334

F1SCC6 ---RITALHLPRFSVSGDYTLHDILPHLGIKKVFSHQADLSGITD-QARLKVSQVVHRAV 339

F6SWD3 ----HMILIFPKFSISGHYGLETILPKLGIHDVFTTQADLSRISG-NRNLKVSKVFHRAV 318

F7CZD2 YFYRKLELHFPKFSISGQIKLEKILPELGLQDLFTEEADFSGITE-QSRLRVSKGVHKAV 343

A0A091CQE0 ---MSVTIHLPKLSISGNYDLKTVLGNLGITKVFSNAANLSGVTE-DAPLKLSKAVHKAV 333

G5B496 ---TSANLHFPKLSISGNYDLKIVLGNLGITNIFSNAADLSGVTK-DAPLKLSKAVHKAV 328

H0WJ03 ---RSANVYLPKLSISGTYDMKKVLSELGMTKLFSNGADLSGVTE-EVPLKLSKAVHKAV 334

U3FMP8 ---RSVYLHLPKLSITGTYDLKTVLAQLGITKVFSNGADLSGVTK-EVPLKLSKAVHKAV 336

1qlp ---RSASLHLPKLSITGTYDLKSVLGQLGITKVFSNGADLSGVTE-EAPLKLSKAVHKAV 337

G1S644 ---RSASLYLPKLSITGTYDLKTVLGQLGITKVFGNGADLSGVTE-EAPLKLSKALHKAV 336

P01010 ---RSANLHLPKLAITGTYDLKTVLGHLGITKVFSNGADLSGVTE-DAPLKLSKAVHKAV 336

F7CYP1 ---VYANVSLPKLSISGCYDLKRILPELGITKVFSLEADLSGIAE-ETSLTVSKALHKAV 341

B5BV12 ---SFVNVHLPKLSISGTYDLTSILPELGITKVFSRQADLSGITE-EAPLTVSKALHKAV 339

B5BV05 ---SFVNVHLPKLSISGTYDLTSILPELGITKVFSRQADLSEITE-EVPLTVSKALHKAV 339

L8YAZ3 ---RSANLQFPKFTISGTYDLKEVLSDMGITKVFGSEADLSGITE-DAPLKLSKAVHRAV 336

G3TDW3 ---RSAILSFPKLSISGTYDLKNLLGKLGITHVFSDGADLSGISE-SEGLKLSKAVHKAV 337

G1PM73 ---RSADLSMPKLSISGTYDLKAILGEMGITKVFSNGADLSGISE-EVPLKVSKAMHKAV 333

L5LNJ6 ---RSASLSMPKLSISGTYDLKAILGEMGITKVFSNGAELSGISE-EVPLKLSKAVHKAV 335

L5JP19 ---RSANLQLPKLSISGTYDLATVLGKLGITKVFSNAADLSGISE-QEPLKLSKAVHKAV 331

M3WCX1 ---RSANLHLPKLSISGTYDLQTVLRKMGITKVFSNEADLSGITE-QGPLKLSKALHKAV 337

F1PCE5 ---RSASLRLPKLSISGTYDLKSVLSKMGITKVFSAEADLSGITE-EGPLMLSKGLHKAV 335

M3XVV7 ---RSANLRLPKLTISGTYDLKTVLSKMGITRVFSNDADLSGITE-KEPLKLSKGVHKAV 317

D2HEM3 ---RSASLRLPKMSISGTYDLKTVLSKMGITKVFSNEAELSGITE-KEPLKLSKGLHKAV 339

P50447 ---SSANLHLPKLTISGTYDLKSLLGNLGITKVFSDEADLSGVTE-EQPLKLSKALHRAV 337

W5PZS7 ---SSANLHLPKLSISETYDLKTVLGELGINRVFSNGADLSGITE-EQPLMVSKALHKAA 332

P34955 ---SSANLHLPKLSISETYDLKSVLGDVGITEVFSDRADLSGITK-EQPLKVSKALHKAA 332

P23035 ---RSVTVHFPKLSISGTYDLKPLLGKLGITQVFSDNADLSGITE-QEPLKASQALHKAV 333

Q07298 ---RSVRVRFPKLSISGTYDLKPLLGKLGITQVFSDNADLSGITE-QEPLKVSQALHKAV 333

O62663 ---RSVTVRFPKLSISGTYDLKPLLGKLGITQVFSDNTDLSGITE-QEALKVSKALHKAV 333

O54761 ---SSVNLHFPKLNISGTMDLKPVLTRLGITNVFSYKADLSGITE-DDPLRVSQALHKAV 333

O54763 ---RTVNLYFPKLSITGTYDLRSVLSTLGITKVFSNEADLSGVTE-EAPLKLSKGVHKAV 333

Q76HP1 ---SKYQVYFPRVSISGTYDLKDVLSSLGITRVFSRVADLSGVTE-DAPLTVSKVLHKAV 333

Q76HP0 ---TLVNLYFPKVSISGTYDLKTVLHSLGITKVFSQEADLSGVTE-EAPLTVSKGLHKAV 333

Q76HN9 ---TRVSLYFPKVSISGTYGLKTVLSSLGITKVFSNAADLSGVTE-EAPLIVSKALHKAV 333

O54760 ---TKSQLYFPKVSISGTYDLKDVLSSLGITKVFSSEADLSGVTE-EAPLTVSKALHKAV 333

Q64118 ---RSANVHLPKLSISGTYNLKKVLSPLGITQVFSNGADLSGITT-DVPLKLSKAVHKAV 327

P97277 ---RSANVHFPKLSISGTYNLKTALDPLGITQVFSNGADLSGITE-DVPLKLGKAVHKAV 333

G3I296 ---RSANLHFPKLSISGTYDLKTALNALGITQVFSNKADLSGITE-DAPLKLGKAVHKAV 333

P17475 ---RSAILYFPKLSISGTYNLKTLLSSLGITRVFNNDADLSGITE-DAPLKLSQAVHKAV 332

Q63969 ---SLAEIHFPRLSISGSYNLKALMAPLGITRVFNNGADLSGITEENAPLRLSKAVHKAV 333

P07758 ---RLAQIHFPRLSISGEYNLKTLMSPLGITRIFNNGADLSGITEENAPLKLSQAVHKAV 333

Q00898 ---RLAQIHIPRLSISGNYNLETLMSPLGITRIFNSGADLSGITEENAPLKLSQAVHKAV 333

: :* : : . * ::* : : *

A0A093GX57 VDVNENGTEAAAVTVVEITATSL--QLPPPPVIRFNRPFLMLIFDKAA-SSIIFIGKIVN 383

A0A099YYG0 VDVNENGTEAAAVTVVEIIPMSL--PIPPPPVIKFNRPFMMIIFDKTV-HSILFLGKVMN 383

G1NKH9 LNINENGTEAAAVTMVEMKVFSA---MVTPLEIKFNRPFLMMIFDKTT-NSILFMGKVVN 392

U3IXC3 VDVNENGTEAAAVTMMELIRLSA--AFPLPHVIKFNRPFLMMIVDKTT-YSILFMGKIVN 390

A0A091KYD1 VDVSENGTEAAAVTVIGIVGMSA--HFHSPPHIRFNRPFLMLIIDKST-DGLLFMGKIVK 381

A0A091QVX2 VDVSENGTEAAAVTMIEMVPLSA--MFPPPTHIRFNRPFLMMIIDRNT-QSMLFMGKIMN 378

A0A091NC43 VDVSENGTEAAAVTVVEIMLMSSM-FETPRPHIKFNRPFLMMIMNKDT-HSILFMGKIVN 384

A0A091GSL7 VDVSENGTEAAAVTVIQVVEVSA--LSPPPPHIRFNRPFLMLVFNKPT-QNILFMGKIVN 381

A0A093PGJ8 VDVRENGTEASAATMLEITLLSR--RFPLPLHITFNRPFLMMIIDKTT-HSMLFLGKIVN 383

A0A091NIR3 VDVRENGTEAAAVTVIEVTLYSI--EIPPPPHISFNKPFLMIIIDKIT-NSILFLGKIVN 383

A0A091G0Q5 VDVRENGTEAAAATVIEAVPMSA--VIPPPPHISFNRPFLMLIFDEIT-RSILFMGKIMN 383

A0A093GVB9 VDVSENGTEAAAVTAIEIVPLSA--MFPPPPHIRFNRPFLLMITDKTT-RSILFMGKVVN 381

A0A093CUY6 VDVRENGTEAAAATVVELMLMSAGPASPPPLHIKFNRSFLMMIIDKTT-LNILFMGKIVN 385

A0A091QM83 VDVSENGTEAAAATVIELVPLSA--EIPPPPHITFDRPFLMIIAEKST-HSILFIGKIVN 383

A0A091PDX7 VDVSENGTEAAAVTMIELTLLSA--RFPPPPHIRFNRPFLMMIVDKTS-HGILFMGKIVN 383

A0A091WBP3 VDVSENGTEAAAVTEIEMVLMSA--QIPRPPYIRFNRPFLMMIIDQIS-HSILFMGKIVN 383

A0A091SNX6 VDVSENGTEAAAATAIEVVLLSA--LFPSPPHIRFNRPFLMMIVDKTT-HSILFMGKIVN 383

A0A094KEC1 VDVSENGTEAAAVTVIEMVPLSA--EFPPPPHISFNKPFLMMVIDKTT-DGVLFMGKIMN 383

A0A0A0ABV8 VDVSENGTEAAAVTVIEAVAMSA--QIPPPPHIRFNKPFLMIIVDKTT-HGILFMGKIVN 383

A0A093JEM7 VDVSENGTEAAAVTVIEVVPLSA--AFPPPPHIVFNRPFLMITVDKIT-HSILFMGKIVN 381

A0A093G1U7 VDVSENGTEAAAVTVIELVPLSA--EFPPPPHITFNRPFLMIIIDKTT-HSILFMGKIVN 383

A0A091JCG9 VDVSENGTEAAAVTVVELVLLSA--AYPLPPHIRFNRPFLMMIVDKTT-HSILFMGKIVN 383

A0A091U8N8 VDVSENGTEAAAVTVIEVVPLSA--EFPPPPQIKFNRPFLMIIVDKIS-HGILFMGKIVN 383

A0A091RXN3 VDVSENGTEAAAVTLIEMVPLSA--EFPPPPHIRFNRPFLMMIIDKIT-HSILFMGKIVN 381

A0A093R765 VDVSENGTEAAAVTVIELVPLSA--VIPPPPHIRFNRPFLMMIIDKTT-HGILFMGKIVN 383

A0A087VMC1 VDVSENGTEAAAVTAIELVPLSA--GFPPPPRIRFNRPFLMIIVDKST-HGILFMGKIVN 383

A0A093F995 VDVSENGTEAAAVTVIEVSLLSA--LFPLPPHIRFNRPFLMMVFDKTT-HSILFMGKIVN 383

A0A093DUW7 VDVSENGTEAAAVTVIEVVPMSA--EFPPPPRIRFNRPFLMMIVDKTT-HSILFMGKIVN 383

A0A091T9D5 VDVSENGTEAAAVTMIELVPLTA--EFPPPPHIRFNRPFLMMIIDKTT-HGILFMGKIVN 383

A0A091LUF5 VDVSENGTEAAAVTVIEMVPLLA--SFPPPPHIRFNRPFLMVILDKTT-HSILFMGKIVN 383

A0A091V8N3 VDVSENGTEAAAVTMIELVPLSA--PFPLPPHIKFNRPFLMIIVDKTT-HGILFMGKIVN 383

A0A091LFJ1 VDVSENGTEAAAVTVIEMVPMSA--EIPPPPHIRFNRPFLMIILDKTT-HSILFIGKIVN 383

A0A087R544 VDVSENGTEAAAVTVIEVVPLSA--QFPPPPHITFNRPFLMMIVDKTT-YGILFMGKIVN 383

A0A091LM39 VDVSENGTEAAAVTVIELVPLSA--EFPPPPHIRFNRPFLMIIVDKTT-HSIPFMGKIVN 383

Q66KX6 LSIDETGTEAAGVTGMELM------PMMVPSRIEFNKPFLIIIYGQET-RSNYFMGRIMN 386

A0A099Z110 LNIHENGTEASGGTYTELV------PHSVPPVVKFDRPFLLLIIDQYT-HSILFMGKIVN 369

G1NKI3 LNIHENGTEAAAVTGTEFA------PHSVPPVIKFNRPFLLLIVDQYT-QSILFIGKIVN 395

E1C206 LDIHENGTEAAAVTGTEFA------PHSVPPVIKFNRPFLLLIVDQYT-ESILFIGKIVN 393

R0LEI7 LNIHENGTEAAAVTGTDFA------PHSVPPVVKFNRPFLLLIVDQYT-QSILFMGKIVN 388

A0A091F2Q1 LKIHENGTEAAATSSIDFL------PHSAPPIVKFNHPFLLLIVDQYT-QSILFMGKIVN 369

A0A093IN74 LKIHENGTEAAAVSGTDFV------PHSVPPVVKFNRPFLLLIVDQYT-QSILFMGKIVK 369

A0A093C116 LKIHENGTEAAAVTGIDFL------PHSVPPVVKFNHPFLLLVVDQYT-QSILFMGKIVN 369

A0A093PVA9 LKIHENGTEAAAVTGIDFL------PHSLPPIVKFDRPFLLLIVDQYT-QSILFMGKIVN 369

A0A091KNU4 LKIHENGTEAAAVTSTDFL------PHSVPPVLKFNRPFLLLIVDQYT-QSILFMGKIVN 368

A0A087VMC2 LKIHENGTEVAAVSGTDFL------PHSVPPVVKFNRPFLLLIVDQHT-QSILFMGKIIN 369

A0A091PCC0 LKIHENGTEAAAVTGTDFL------PHSVPPAVKFNRPFLLLIVDQYT-QSILFMGKIVN 359

A0A093FFU5 LKIHENGTDAAAVTGTDFL------PHSVPPVVKFNRPFLLLIVDQYT-QSILFMGKIVN 362

A0A091XH23 LKIHENGTEAAAVTGTDFL------PHSVPPAVKFNRPFLLLIVDQYT-QSILFMGKIVN 369

A0A091T7I8 LEVHENGTEAAAVTGTDFL------PHSVPPVVKFNRPFLLLIVDQYT-QSILFMGKIVN 359

A0A093CLJ8 LKIHENGTEAAAVTSTDFL------PHSVPPVVKFNHPFLLLIVDQYT-QSILFMGKIVN 369

A0A093KV79 LKIHENGTEAAAVTGTDFL------PHSVPPVVKFNHPFLLLIVDQYT-QSILFMGKIVN 369

A0A087R545 LKIHENGTEAAAVTGTDFL------PHSVPPVVKFNRPFLLLIVDQYT-QSILFIGKIVN 359

G1KWY8 VDVHESGTEAAAVTVIQFVRMSF--SSPPPTVFKCDKPFLFLICDKMT-NSILFFGKVVN 403

G1NKH5 LDVDERGTEVAATTAPKIM------ALTLAPSIEFNRPFLMLIFDRDT-NSTLFIGKIAN 386

A0A093H648 LDVDERGTGAAAATAAEIM------TISLPPTIEFNRPFLMLIFDRAT-NSTLFIGKIVN 370

A0A091PB47 LDVDERGTEAAAATAVEIM------PVSIPPTIEFNRPFLMLIFDRDT-NSTLFIGKIVN 369

A0A093QJ65 LDVDERGTEAATATAGE-M------TMSLPATIEFNHPFLMLIFDRDT-NSTLFIGKIVN 368

A0A091K7I5 VDVDERGSEAAAGTAGEIM------QLSLPPIIEFNRPFLMLIFDRDT-NSTLFIGKIVN 369

A0A091K0Z5 LDVDERGTEAAAANAAEIW------TSSLSQTIEFNQPFLMLIFDRDT-NSTLFIGKIVN 369

R0LHU9 LDVDERGTEAAAATAAEIM------TMSLPPTIEFNRPFLMLIFDRDT-NSTLFIGKIVN 387

H0ZQY2 LDVDEKGTEAAAATAVEIM------PVSFPPTIEFSHPFLMLIFDRDT-NSTLFIGKIVN 370

A0A091GLC8 LDVDESGTEAAAAAAAEIV------TMSLPPTIEFNHPFLLLIFDRDT-NSTLFIGKIVN 369

A0A091HV30 VDVDESGTEAAAATVAEIM------TMSLPPTIEFNHPFLMLIFDRDT-NSTLFIGKIVN 369

A0A093RZ50 LDVDERGTEAAAATAAEIV------PMSLPPTIEFNHPFLMLIFDRDT-NSTLFIGKIVN 369

A0A091SWC2 LDVDERGTEAAAATAAEIM------TMSLPPTIEFNRPFLMLIFDRDT-NSTLFIGKIVN 369

A0A091QU84 LDVDESGTEAAAATAAEIM------TMALPPTIEFNHPFLMMIFDRDT-NSTLFIGKIVN 369

A0A093GS00 LDVDERGTEAAAATAVEIM------PMSLPPTIEFNHPFLMLIFDRDT-NSTLFIGKIVN 369

A0A093IEQ9 LDVDERGTEAAAATAAEIV------TMSLPPTVEFNHPFLMLIFDRDT-NSTLFIGKIVN 369

A0A091PLB4 LDVDESGTEAAAASAVEIM------TASLPPTIEFNQPFLLLIFDRDT-NSTLFIGKIVN 369

A0A093FVA3 LDVDERGTEAAAATAAEIM------TVSLPPTIEFNHPFLMMIFDRDT-NSTLFIGKVVN 369

A0A091U6W4 LDVDERGTEAAAATAAEIT------TISLPPTVEFNHPFLMLIFDRDT-NSTLFIGKIVN 369

A0A091KQX5 LDVDERGTEAAAATAAEIM------TASLPPTIEFNHPFLMLIFDRDT-NSTLFIGKIVN 369

A0A091MK58 LDVDERGTEAAAATAAEIT------TVSLPPTIDFNHPFLMLIFDRDT-NSTLFIGKIVN 369

A0A091T7N1 LDVDERGTEAAAATAAEMM------TMSLPPTIEFNRPFLMLIFDIAT-NSTLFIGKIVN 369

A0A0A0A7A0 VDVDERGTEAAAATAAEIV------TVSLPPTIEFNRPFLMLIFDRDT-NSTLFIGKIVN 369

A0A091VAK7 LDVDERGTEAAAATAAEIV------TMSLPPTIEFNRPFLMLIFDRDT-NSTLFIGKIVN 369

A0A093P9B6 LDVDERGTEAAAATAAEIM------TVSLPPTIEFNHPFLMLIFDRDT-NSTLFIGKIVN 369

K7FR61 LNVDERGTEAAAATAIEIM------PMSMPQTIEYNCPFLMLIFEKTT-NSTLFIGKINN 389

M7B3F1 LNIDERGTEAAAATAIEIM------PMSFPHIIEFNFPFLVLIFDRAT-NSTLFIGKINN 368

K7FUR6 LTVHENGTEAAAATAIVIS---K--LFRPSFIIKYNMPFLVMIVEKRS-LNILFMGKIVN 388

Q8JIA6 LNVHENGTEASATTVIELV------PMSLPPVVKLNRPFLFSIVDKTA-HSVLFMGKVVN 385

A0A093H779 LNVHENGTEAAGVTVKEIIWRSG--EFPAPRRVKFNRPFLLMILDKFT-HTILFIGKIVN 370

G1NKH1 LNVHENGTEAAGVTVKEITWRSG--DISRPPRVRFNRPFLLMILDKYA-HTILFIGKIVN 379

U3JIK3 LNVHENGTEAAGATVKEVTWRSG--DFPRPPRVRFNRPFLLMILDKFT-HTVLFIGKIVN 383

A0A091EGC4 LNVHENGTEAAGATVKEVTWRSG--DFPRPPRVRFNRPFLLMILDKFT-RSVLFIGKIVN 370

A0A093PHW0 LNVHENGTEATGVTVKEVTWRSG--DFPRPPRVRFNRPFLLVILDKFT-HTVLFIGKIVN 370

A0A091NZH9 LNVDENGTEAAAVTVKEITWRSG--DFPRPPRVKFNRPFLLVILDKFT-HTILFIGKIVN 362

R0JRB7 LSVHENGTEAAGATVKEITWRSG--DFPRPPRIKFNRPFLLTIVDKYT-RTVLFIGKIVN 396

A0A091KRT6 LNVHENGTEAAGATLVEIIRKSG--DIPRPHRVRFNRPFLLVILDKYT-RTVLFIGKIVN 370

A0A091FX98 LSVHENGTEAAGATVMEVTWRSG--DFPQPPRVRFNRPFLLVILDKCT-RTVLFIGKIVN 370

A0A091LT78 LNIHENGTEAVGVTVKEITWRSG--DFPHPPRIRFNRPFLLVILEKYT-CTVLFIGKIVN 367

A0A091QJI6 LNVHENGTEAAGTTVKEVTWRSG--DFPHPPRVRFNRPFLLVILDKYT-RTILFIGKIVN 365

A0A093H5C6 LNIHENGTEAAGATVKEITWRSG--DFPHPPRVKFNRPFLLVILDKYT-RTVLFMGKIVN 370

A0A091RAD2 LNVHENGTEAAGVTVKEVTWRSG--EFPHPPRVRFNRPFLLVILDKYT-RTVLFIGKIIN 363

A0A091KQF0 LNLHENGTEAAGATVKEVTWRSG--DFPHPPRVKFNRPFLLVILDKYT-RTVLFMGKIVN 370

A0A093IZH1 LNVHENGTEAAGTTVKEITWRSG--DFPHPPRVRFNRPFLLMILDKYT-RTVLFIGKIVN 370

A0A091V9J5 LNVHENGTEAAGATVKEVTWRSG--DFPHPPRVRFNRPFLLLILDKYT-RTVLFIGKIVN 373

K7FUQ7 VNVHENGTEAAGATITEISWRSG--RI--PPRIKFNRPFLLMIVDRNI-RSILFMGKIIN 374

M7B3E7 LNVHENGTEAAGVTVVEISWRSG--QISAPPRIKFNRPFLLMIFDRHT-RSILFLGKIIN 403

K7FUC4 LNVHENGTEAAAVTVAEMV------PTSLPPVLRINRPFLILIIDRNT-DFLLFLGKIVN 384

K7FSH8 VDVHENGTEAAAVTVVEIMFMSA--PIDDPPTIKFNRPFIIIIIDKTT-CSMLFLGKIVN 405

M7B5J6 VDVHENGTEAAAVTVVEIAFFSA--EFPPPPTIKFNRPFLMMILDKPT-RSILFMGKIIN 403

S7ME51 LDVDEVGTEAAAATSLSVTLFSA--PRIPG-ILKFNRPFLVAILSTDT-QSILFLGKVVN 392

M3XVL7 LEVDEVGTQAAAATGSFVTFMSA--QHNRR-VLRFNRPFLVVIFSTNS-QSIVFLGKVVN 400

K9IXP7 IDVAENGTEAAAATAISLSLMSL---RIPTISVNFNSPFLLAILSKDT-DSILFCAKVAN 390

A0A0D9REC0 LDVSEEGTEASAATGVKITLLSA--FVDPKITVRFNRPFLMIIVPMDT-QNIFFISKVIN 396

K7A8Q9 LDVFEEGTEASAATAVRITLLSA--LVETRTIVRFNRPFLMIIVPTDT-QNIFFMSKVTN 396

Q5R536 LDVFEEGTEASAATAVKITLLSA--LVDPMTIVRFNRPFLMIIVPTDT-QNLLFISKVIN 396

A2I7N2 LDVDEEGTEGAAATGIGIERTFL-----R-IIVRVNRPFLIAVVLKDT-QSIIFLGKVTN 387

F1SCC6 LDVDEEGTEGAAATGISIELTSI-----EFLTVHFNRPFLFSIIHKDT-QSIIFSGKVTD 393

F6SWD3 LDVGEKGTEAAAATGIKIMYKSA--PFIPPLVVHFNKPFLMYVFDKLT-GSILFLGKVVN 375

F7CZD2 LDIGEEGTEASAATSLGTVFLSA--PKITQ-PVRFNRPFLVVILSTET-QSLLFLGKVIN 399

A0A091CQE0 LTIDEKGTEAAGATVLEAV------PMSLPPDVHFNHPFIVIIIDHST-DSPLFVGRVMD 386

G5B496 LTIDEKGTEAAGATVLEAI------PMSMPPNVYFIRPFIFTIIDHST-YSPLFVGKVMD 381

H0WJ03 LTLDEKGTEAAGATFMEAI------PMSIPPDVKLNRPFVFLIYYEIT-KSPLFVGKIVN 387

U3FMP8 LTMDEKGTEAAGTTIFEAI------PMSMPPEVKFNKPFIFLMFEQNT-KSPLFMGKVVN 389

1qlp LTIDEKGTEAAGAMFLEAI------PMSIPPEVKFNKPFVFLMIEQNT-KSPLFMGKVVN 390

G1S644 LTIDEKGTEAAGVMFLEAI------PMSIPPEVKFNKPFVFLMIEQNT-KSPLFMGKVVN 389

P01010 LTIDEKGTEAAGAMFLEAI------PMSIPPEVKFNKPFVFLMIEQNT-KSPLFIGKVVN 389

F7CYP1 LTIDEKGTEAAGATIVEAI------RTLLHTNVEFNRPFVLIIYDRNT-KSPLFVGKVVD 394

B5BV12 LTIDEKGTEAAGATMWEMI------PMSLPPELEFNRPFILIIYGRNT-KSPLFVGKVVD 392

B5BV05 LTIDEKGTEAAGTTMWEIM------PISLPPDLKFNRPFVLIIYDRNT-KSPLFVGKVVD 392

L8YAZ3 LTIDEKGTEAAGATVLEAI------PMSIPPEVSFNKPFVIIIYDHVT-HSPLFMGKVVN 389

G3TDW3 LTIDEKGTEASGATILEAI------PMSMPPTVKFNSPFLAIIYDENT-KSPLFVGKVVN 390

G1PM73 LTLDEKGTEAAGATYMEIM------PMSLPPDIEFNRPFVVVIYDTVT-KAPLFVGKVVN 386

L5LNJ6 LTLDEKGTEAAGATFGEVM------PMSLPPNIEFNRPFVLVIYDTVT-KAPLFVGKVVN 388

L5JP19 LTIDEKGTEASAATILEAI------PMSIPPTVQFNRPFLFIIYDKNT-KSPLFMGKVMN 384

M3WCX1 LTIDEKGTEAAGTTVMEAI------PMSMPPTIQFNKPFVIILYDRNT-KNILFMGKVVN 390

F1PCE5 LTIDEKGTEAAGATFLEAI------PMSMPPSVDFNKPFLIIIVDRDT-KSPLFMGKVVN 388

M3XVV7 LTIDEKGTEATGATFMEAI------PMSMPPSVDFNSPFLIIIYDRNT-KSPLFVGKVVN 370

D2HEM3 LTVDEKGTEAAGTTVLEAI------PMSMPPVVDFSSPFLVIIYDRNT-KSPLFVGKVVD 392

P50447 LTIDEKGTEATGATILEAI------PMSIPPNVKFNKPFLFLIYDTKT-KAVLFMGKVMN 390

W5PZS7 LTIDEKGTEAAGATFLEAI------PMSLPPDVEFNRPFLCILYDRNT-KSPLFVGKVVN 385

P34955 LTIDEKGTEAVGSTFLEAI------PMSLPPDVEFNRPFLCILYDRNT-KSPLFVGKVVN 385

P23035 LTIDERGTEAAGATYMEII------PMSLPDSITLDRPFLFVIYSHEI-KSPLFVGKVVD 386

Q07298 LTIDERGTEAAGATFVGIM------PSSLPESVIFDRPFLFVIYSHEL-KSPLFVGKVVD 386

O62663 LTIDERGTEAAGATFVEYV------LYSMPQRVTFDRPFLFVIYSHEV-KSPLFVGKVVD 386

O54761 LTIDERGTEAAGATFLEMM------PMSLPPEVKFDKPFLVVIIEHST-KSPLFVGKVVN 386

O54763 LTIDERGTEAAGVTVLEAI------PMSLPPDVRFDRPFLIIIYEHYT-KSPLFVGKVVN 386

Q76HP1 LDMDEEGTEAAGATVLGAE------AMLQAPIMKFDRPFLVVIYEHNT-KSPLFVGKVVN 386

Q76HP0 LDIHEKGTDAAGATFLEMI------PMMLPSDLKFDRPFLVVIYEHHT-KSPLFVGKVVN 386

Q76HN9 LDIDEEGTEAAGGTVGGIT------FMSRPDEVIFDRPFLVVIYEHHT-KSPLFVGKLVN 386

O54760 LDIDEEGTEAAGGTVLGNI------RSILRYEVIFDRPFLVVIYEHHT-KSPLFVGKVVN 386

Q64118 LTLDERGTEAAGTTVLEAV------PMSIPPDVCFKNPFVVIICDKHT-QSPLFVGKVVN 380

P97277 LTIDERGTEAAGATFMEII------PMSVPPEVNFNSPFIAIIYDRQTAKSPLFVGKVVD 387

G3I296 LTLDERGTEAAGATVFEII------PMSVPPEVIFNSPFLAIIYDRQT-QSPLFVGKVVD 386

P17475 LTLDERGTEAAGATVVEAV------PMSLPPQVKFDHPFIFMIVESET-QSPLFVGKVID 385

Q63969 LTIDERGTEAAATTIVEAV------FMSLPPILHFNHPFVFTIVETHT-QTPLFVGKVVD 386

P07758 LTIDETGTEAAAVTVLQMV------PMSMPPILRFDHPFLFIIFEEHT-QSPIFLGKVVD 386

Q00898 LTIDETGTEAAAATVLQGG------FLSMPPILHFNRPFLFIIFEEHS-QSPLFVGKVVD 386

: : * *: . *: * .:: .

A0A093GX57 P--- 384

A0A099YYG0 P--- 384

G1NKH9 P--- 393

U3IXC3 PTLK 394

A0A091KYD1 P--- 382

A0A091QVX2 P--- 379

A0A091NC43 P--- 385

A0A091GSL7 P--- 382

A0A093PGJ8 PT-- 385

A0A091NIR3 P--- 384

A0A091G0Q5 P--- 384

A0A093GVB9 P--- 382

A0A093CUY6 P--- 386

A0A091QM83 P--- 384

A0A091PDX7 P--- 384

A0A091WBP3 P--- 384

A0A091SNX6 P--- 384

A0A094KEC1 P--- 384

A0A0A0ABV8 P--- 384

A0A093JEM7 P--- 382

A0A093G1U7 P--- 384

A0A091JCG9 P--- 384

A0A091U8N8 P--- 384

A0A091RXN3 P--- 382

A0A093R765 P--- 384

A0A087VMC1 PT-- 385

A0A093F995 P--- 384

A0A093DUW7 P--- 384

A0A091T9D5 P--- 384

A0A091LUF5 P--- 384

A0A091V8N3 P--- 384

A0A091LFJ1 P--- 384

A0A087R544 P--- 384

A0A091LM39 P--- 384

Q66KX6 PKK- 389

A0A099Z110 PT-- 371

G1NKI3 PLK- 398

E1C206 PLK- 396

R0LEI7 P--- 389

A0A091F2Q1 PT-- 371

A0A093IN74 PT-- 371

A0A093C116 PT-- 371

A0A093PVA9 PT-- 371

A0A091KNU4 PT-- 370

A0A087VMC2 P--- 370

A0A091PCC0 PT-- 361

A0A093FFU5 PT-- 364

A0A091XH23 PT-- 371

A0A091T7I8 PT-- 361

A0A093CLJ8 PT-- 371

A0A093KV79 PT-- 371

A0A087R545 PT-- 361

G1KWY8 PTEK 407

G1NKH5 PS-- 388

A0A093H648 PT-- 372

A0A091PB47 PT-- 371

A0A093QJ65 PT-- 370

A0A091K7I5 PT-- 371

A0A091K0Z5 PT-- 371

R0LHU9 PT-- 389

H0ZQY2 PT-- 372

A0A091GLC8 PS-- 371

A0A091HV30 PT-- 371

A0A093RZ50 PT-- 371

A0A091SWC2 PT-- 371

A0A091QU84 PT-- 371

A0A093GS00 P--- 370

A0A093IEQ9 PT-- 371

A0A091PLB4 PT-- 371

A0A093FVA3 PT-- 371

A0A091U6W4 P--- 370

A0A091KQX5 PT-- 371

A0A091MK58 PT-- 371

A0A091T7N1 PT-- 371

A0A0A0A7A0 PT-- 371

A0A091VAK7 PT-- 371

A0A093P9B6 PT-- 371

K7FR61 PLE- 392

M7B3F1 PAE- 371

K7FUR6 PTIK 392

Q8JIA6 PKEK 389

A0A093H779 P--- 371

G1NKH1 PLKK 383

U3JIK3 PQK- 386

A0A091EGC4 P--- 371

A0A093PHW0 P--- 371

A0A091NZH9 P--- 363

R0JRB7 PLK- 399

A0A091KRT6 P--- 371

A0A091FX98 P--- 371

A0A091LT78 P--- 368

A0A091QJI6 P--- 366

A0A093H5C6 P--- 371

A0A091RAD2 P--- 364

A0A091KQF0 P--- 371

A0A093IZH1 P--- 371

A0A091V9J5 P--- 374

K7FUQ7 PN-- 376

M7B3E7 PSGK 407

K7FUC4 PNE- 387

K7FSH8 PTEK 409

M7B5J6 PIEK 407

S7ME51 PTE- 395

M3XVL7 PTK- 403

K9IXP7 PKE- 393

A0A0D9REC0 PKQ- 399

K7A8Q9 PKQ- 399

Q5R536 PKQ- 399

A2I7N2 PSE- 390

F1SCC6 PSQ- 396

F6SWD3 PTLK 379

F7CZD2 PKDQ 403

A0A091CQE0 PTQK 390

G5B496 PTQK 385

H0WJ03 PTQK 391

U3FMP8 PTQK 393

1qlp PTQK 394

G1S644 PTQK 393

P01010 PTQK 393

F7CYP1 PTQK 398

B5BV12 PTQK 396

B5BV05 PTQK 396

L8YAZ3 PTLK 393

G3TDW3 PLK- 393

G1PM73 PTQK 390

L5LNJ6 PTQK 392

L5JP19 PTQK 388

M3WCX1 PT-- 392

F1PCE5 PTQK 392

M3XVV7 PTQ- 373

D2HEM3 PTQK 396

P50447 PTQK 394

W5PZS7 PTQ- 388

P34955 PTQ- 388

P23035 PTQ- 389

Q07298 PTQ- 389

O62663 PTQ- 389

O54761 PT-- 388

O54763 PTQ- 389

Q76HP1 PTQQ 390

Q76HP0 PTQQ 390

Q76HN9 PTQQ 390

O54760 PTQQ 390

Q64118 PTQ- 383

P97277 PT-- 389

G3I296 PT-- 388

P17475 PT-- 387

Q63969 PTRK 390

P07758 PTHK 390

Q00898 PTHK 390

*

ClustalW Omega MSA of the human inhibitory serpins.

**>sp|P01009|A1AT_HUMAN Alpha-1-antitrypsin OS=Homo sapiens OX=9606 GN=SERPINA1 PE=1 SV=3**

>sp|P01008|ANT3_HUMAN Antithrombin-III OS=Homo sapiens OX=9606 GN=SERPINC1 PE=1 SV=1

>sp|P05121|PAI1_HUMAN Plasminogen activator inhibitor 1 OS=Homo sapiens OX=9606 GN=SERPINE1 PE=1 SV=1

>sp|P05155|IC1_HUMAN Plasma protease C1 inhibitor OS=Homo sapiens OX=9606 GN=SERPING1 PE=1 SV=2

>sp|Q99574|NEUS_HUMAN Neuroserpin OS=Homo sapiens OX=9606 GN=SERPINI1 PE=1 SV=1

>sp|P01011|AACT_HUMAN Alpha-1-antichymotrypsin OS=Homo sapiens OX=9606 GN=SERPINA3 PE=1 SV=2

>sp|P29622|KAIN_HUMAN Kallistatin OS=Homo sapiens OX=9606 GN=SERPINA4 PE=1 SV=3

>sp|P05154|IPSP_HUMAN Plasma serine protease inhibitor OS=Homo sapiens OX=9606 GN=SERPINA5 PE=1 SV=3

>sp|Q9UK55|ZPI_HUMAN Protein Z-dependent protease inhibitor OS=Homo sapiens OX=9606 GN=SERPINA10 PE=1 SV=1

>sp|P30740|ILEU_HUMAN Leukocyte elastase inhibitor OS=Homo sapiens OX=9606 GN=SERPINB1 PE=1 SV=1

>sp|P05120|PAI2_HUMAN Plasminogen activator inhibitor 2 OS=Homo sapiens OX=9606 GN=SERPINB2 PE=1 SV=2

>sp|P29508|SPB3_HUMAN Serpin B3 OS=Homo sapiens OX=9606 GN=SERPINB3 PE=1 SV=2

>sp|P48594|SPB4_HUMAN Serpin B4 OS=Homo sapiens OX=9606 GN=SERPINB4 PE=1 SV=2

>sp|P35237|SPB6_HUMAN Serpin B6 OS=Homo sapiens OX=9606 GN=SERPINB6 PE=1 SV=3

>sp|O75635|SPB7_HUMAN Serpin B7 OS=Homo sapiens OX=9606 GN=SERPINB7 PE=1 SV=1

>sp|P50452|SPB8_HUMAN Serpin B8 OS=Homo sapiens OX=9606 GN=SERPINB8 PE=1 SV=2

>sp|P50453|SPB9_HUMAN Serpin B9 OS=Homo sapiens OX=9606 GN=SERPINB9 PE=1 SV=1

>sp|P48595|SPB10_HUMAN Serpin B10 OS=Homo sapiens OX=9606 GN=SERPINB10 PE=1 SV=1

>sp|Q96P63|SPB12_HUMAN Serpin B12 OS=Homo sapiens OX=9606 GN=SERPINB12 PE=1 SV=1

>sp|Q9UIV8|SPB13_HUMAN Serpin B13 OS=Homo sapiens OX=9606 GN=SERPINB13 PE=1 SV=2

>sp|P05546|HEP2_HUMAN Heparin cofactor 2 OS=Homo sapiens OX=9606 GN=SERPIND1 PE=1 SV=3

>sp|P07093|GDN_HUMAN Glia-derived nexin OS=Homo sapiens OX=9606 GN=SERPINE2 PE=1 SV=1

>sp|P08697|A2AP_HUMAN Alpha-2-antiplasmin OS=Homo sapiens OX=9606 GN=SERPINF2 PE=1 SV=3

>sp|O75830|SPI2_HUMAN Serpin I2 OS=Homo sapiens OX=9606 GN=SERPINI2 PE=1 SV=1

CLUSTAL Omega (1.2.4) MSA of the human inhibitory serpins.

CLUSTAL O(1.2.4) multiple sequence alignment

sp|P08697|A2AP_HUMAN ------------------MALLWGLLVLSWSCLQGPCSVFS----PVSAMEPLGRQLTS- 37

sp|P05155|IC1_HUMAN ---------MASRLTLLTLL--LLLLAGDRASS--NPNATS-----SSSQDPESLQDRGE 42

sp|Q9UK55|ZPI_HUMAN -------------MKVVPSLLLSVLLAQVWLVPGLAPSPQSPE-----TPAPQNQTSRV- 41

sp|P01009|A1AT_HUMAN ---------MPSSVSWGILL----L------AGLCCLVPVSLA-----E-DPQGDAAQK- 34

sp|P29622|KAIN_HUMAN ----------MHLIDYLLLLLVGLL------ALSHGQLHVE----------HDGESCSN- 33

sp|P01011|AACT_HUMAN ------------MERMLPLLALGLL------AAGFCPAVLC----------HPNSPLDE- 31

sp|P05154|IPSP_HUMAN -------------MQLFL---------------LLCLVLLS----------PQGASLHR- 21

sp|P05121|PAI1_HUMAN ------------------------------------------------------------ 0

sp|P07093|GDN_HUMAN ------------------------------------------------------------ 0

sp|Q99574|NEUS_HUMAN ------------------------------------------------------------ 0

sp|O75830|SPI2_HUMAN ------------------------------------------------------------ 0

sp|P05546|HEP2_HUMAN ---------MKHSL---NALLIFLIITSAWGGSKGPLDQLEKGGETAQSADPQWEQLNN- 47

sp|P01008|ANT3_HUMAN MYSNVIGTVTSGKR---KVYLLSLLLIGFWDCVTCH-------GSPVD-------ICTA- 42

sp|O75635|SPB7_HUMAN ------------------------------------------------------------ 0

sp|P48595|SPB10_HUMAN ------------------------------------------------------------ 0

sp|Q96P63|SPB12_HUMAN ------------------------------------------------------------ 0

sp|P29508|SPB3_HUMAN ------------------------------------------------------------ 0

sp|P48594|SPB4_HUMAN ------------------------------------------------------------ 0

sp|Q9UIV8|SPB13_HUMAN ------------------------------------------------------------ 0

sp|P05120|PAI2_HUMAN ------------------------------------------------------------ 0

sp|P30740|ILEU_HUMAN ------------------------------------------------------------ 0

sp|P50453|SPB9_HUMAN ------------------------------------------------------------ 0

sp|P35237|SPB6_HUMAN ------------------------------------------------------------ 0

sp|P50452|SPB8_HUMAN ------------------------------------------------------------ 0

sp|P08697|A2AP_HUMAN -GPNQEQVSPLTLLKLGN--------------------------Q--------------- 55

sp|P05155|IC1_HUMAN GKVATTVISKMLFVEP-ILEVSSLPTTNSTTN--SATKITANTTDEPTTQPTTEPTTQPT 99

sp|Q9UK55|ZPI_HUMAN -VQAP--------------------KEE-------------------------------- 48

sp|P01009|A1AT_HUMAN -TDTS--------------------H---------------------------------- 39

sp|P29622|KAIN_HUMAN -SSHQ--------------------QIL-------------------------------- 40

sp|P01011|AACT_HUMAN -ENLT--------------------QEN-------------------------------- 38

sp|P05154|IPSP_HUMAN --HHP--------------------REM-------------------------------- 27

sp|P05121|PAI1_HUMAN -----MQMSPALTCLV-------------------------------------------- 11

sp|P07093|GDN_HUMAN -------MNWHLPLFL-------------------------------------------- 9

sp|Q99574|NEUS_HUMAN ----------MAFLGL-------------------------------------------- 6

sp|O75830|SPI2_HUMAN ----------MDTIFL-------------------------------------------- 6

sp|P05546|HEP2_HUMAN -KNLSMPLLPADFHKENTVTNDWIPEGEEDDDYLDLEKIFSED--------------DDY 92

sp|P01008|ANT3_HUMAN -KPRDIPMNPMCIYR--------SPEK--------------------------------- 60

sp|O75635|SPB7_HUMAN ------------------------------------------------------------ 0

sp|P48595|SPB10_HUMAN ------------------------------------------------------------ 0

sp|Q96P63|SPB12_HUMAN ------------------------------------------------------------ 0

sp|P29508|SPB3_HUMAN ------------------------------------------------------------ 0

sp|P48594|SPB4_HUMAN ------------------------------------------------------------ 0

sp|Q9UIV8|SPB13_HUMAN ------------------------------------------------------------ 0

sp|P05120|PAI2_HUMAN ------------------------------------------------------------ 0

sp|P30740|ILEU_HUMAN ------------------------------------------------------------ 0

sp|P50453|SPB9_HUMAN ------------------------------------------------------------ 0

sp|P35237|SPB6_HUMAN ------------------------------------------------------------ 0

sp|P50452|SPB8_HUMAN ------------------------------------------------------------ 0

sp|P08697|A2AP_HUMAN ----------------EPGGQTALKSPPGVCSRDPTPEQTHRLARAMMAFTADLFSLVAQ 99

sp|P05155|IC1_HUMAN IQPTQPTTQLPTDSPTQPTTGSFCPGPVTLCSDLESHSTEAVLGDALVDFSLKLYHAFSA 159

sp|Q9UK55|ZPI_HUMAN ----------------EEDEQEASEEKASEEEKAWLMASRQQLAKETSNFGFSLLRKISM 92

sp|P01009|A1AT_HUMAN --------------------------------HDQDHPTFNKITPNLAEFAFSLYRQLAH 67

sp|P29622|KAIN_HUMAN -----------------------------ET-GE--GSPSLKIAPANADFAFRFYYLIAS 68

sp|P01011|AACT_HUMAN -----------------------------QDRGTHVDLG---LASANVDFAFSLYKQLVL 66

sp|P05154|IPSP_HUMAN -----------------------------KKRVEDLHVGATVAPSSRRDFTFDLYRALAS 58

sp|P05121|PAI1_HUMAN -----------LGLAL------------VFGEGSAVHHPPSYVAHLASDFGVRVFQQVAQ 48

sp|P07093|GDN_HUMAN -------------LAS------------VTLPSICSHFNPLSLEELGSNTGIQVFNQIVK 44

sp|Q99574|NEUS_HUMAN -----------FSLLV------------LQSMATGATFP----EEAIADLSVNMYNRLRA 39

sp|O75830|SPI2_HUMAN -----------WSLLL------------LFFGSQASRCS----AQKNTEFAVDLYQEVS- 38

sp|P05546|HEP2_HUMAN I-------DIVDSLSVSPTDSDVSAGNILQLFH--GKSRIQRLNILNAKFAFNLYRVLKD 143

sp|P01008|ANT3_HUMAN ----------------KATEDEGSEQ--KIPEA--TNRRVWELSKANSRFATTFYQHLAD 100

sp|O75635|SPB7_HUMAN ---------------------------------------MASLAAANAEFCFNLFREMDD 21

sp|P48595|SPB10_HUMAN ---------------------------------------MDSLATSINQFALELSKKLAE 21

sp|Q96P63|SPB12_HUMAN ---------------------------------------MDSLVTANTKFCFDLFQEIGK 21

sp|P29508|SPB3_HUMAN ---------------------------------------MNSLSEANTKFMFDLFQQFRK 21

sp|P48594|SPB4_HUMAN ---------------------------------------MNSLSEANTKFMFDLFQQFRK 21

sp|Q9UIV8|SPB13_HUMAN ---------------------------------------MDSLGAVSTRLGFDLFKELKK 21

sp|P05120|PAI2_HUMAN ---------------------------------------MEDLCVANTLFALNLFKHLAK 21

sp|P30740|ILEU_HUMAN ---------------------------------------MEQLSSANTRFALDLFLALSE 21

sp|P50453|SPB9_HUMAN ---------------------------------------METLSNASGTFAIRLLKILCQ 21

sp|P35237|SPB6_HUMAN ---------------------------------------MDVLAEANGTFALNLLKTLGK 21

sp|P50452|SPB8_HUMAN ---------------------------------------MDDLCEANGTFAISLFKILGE 21

. .

sp|P08697|A2AP_HUMAN TS-TCPNLILSPLSVALALSHLALGAQNHTLQRLQQVLHAGSG----------------- 141

sp|P05155|IC1_HUMAN MKKVETNMAFSPFSIASLLTQVLLGAGENTKTNLESILSYPKDF---------------- 203

sp|Q9UK55|ZPI_HUMAN R--HDGNMVFSPFGMSLAMTGLMLGATGPTETQIKRGLHLQALKPT-------------- 136

sp|P01009|A1AT_HUMAN QS-NSTNIFFSPVSIATAFAMLSLGTKADTHDEILEGLNFNLTEIP-------------- 112

sp|P29622|KAIN_HUMAN ET-PGKNIFFSPLSISAAYAMLSLGACSHSRSQILEGLGFNLTELS-------------- 113

sp|P01011|AACT_HUMAN KA-PDKNVIFSPLSISTALAFLSLGAHNTTLTEILKGLKFNLTETS-------------- 111

sp|P05154|IPSP_HUMAN AA-PSQSIFFSPVSISMSLAMLSLGAGSSTKMQILEGLGLNLQKSS-------------- 103

sp|P05121|PAI1_HUMAN AS-KDRNVVFSPYGVASVLAMLQLTTGGETQQQIQAAMGFKIDD---------------- 91

sp|P07093|GDN_HUMAN SR-PHDNIVISPHGIASVLGMLQLGADGRTKKQLAMVMRYGVN----------------- 86

sp|Q99574|NEUS_HUMAN TG-EDENILFSPLSIALAMGMMELGAQGSTQKEIRHSMGYDSLK---------------- 82

sp|O75830|SPI2_HUMAN LS-HKDNIIFSPLGITLVLEMVQLGAKGKAQQQIRQTLKQQETS---------------- 81

sp|P05546|HEP2_HUMAN QVNTFDNIFIAPVGISTAMGMISLGLKGETHEQVHSILHFKDFVNASSKY---------- 193

sp|P01008|ANT3_HUMAN SKNDNDNIFLSPLSISTAFAMTKLGACNDTLQQLMEVFKFDTISEKT------------- 147

sp|O75635|SPB7_HUMAN NQ-GNGNVFFSSLSLFAALALVRLGAQDDSLSQIDKLLHVNTASGYGNSSNS-------- 72

sp|P48595|SPB10_HUMAN SA-QGKNIFFSSWSISTSLTIVYLGAKGTTAAQMAQVLQFNRDQGVKCDPESEK------ 74

sp|Q96P63|SPB12_HUMAN DD-RHKNIFFSPLSLSAALGMVRLGARSDSAHQIDEVLHFNEFSQNESKEPDPC-----L 75

sp|P29508|SPB3_HUMAN -S-KENNIFYSPISITSALGMVLLGAKDNTAQQIKKVLHFDQVTENTTGKAA-------- 71

sp|P48594|SPB4_HUMAN -S-KENNIFYSPISITSALGMVLLGAKDNTAQQISKVLHFDQVTENTTEKAA-------- 71

sp|Q9UIV8|SPB13_HUMAN -T-NDGNIFFSPVGILTAIGMVLLGTRGATASQLEEVFHSEKETKSSRIKAE-------- 71

sp|P05120|PAI2_HUMAN AS-PTQNLFLSPWSISSTMAMVYMGSRGSTEDQMAKVLQFNEVGANAVTPMTPENFTSCG 80

sp|P30740|ILEU_HUMAN NN-PAGNIFISPFSISSAMAMVFLGTRGNTAAQLSKTFHFNT------------------ 62

sp|P50453|SPB9_HUMAN DN-PSHNVFCSPVSISSALAMVLLGAKGNTATQMAQALSLNT------------------ 62

sp|P35237|SPB6_HUMAN -D-NSKNVFFSPMSMSCALAMVYMGAKGNTAAQMAQILSFNKSGG--------------- 64

sp|P50452|SPB8_HUMAN ED-NSRNVFFSPMSISSALAMVFMGAKGSTAAQMSQALCLYK------------------ 62

.: : .: : : .: :

sp|P08697|A2AP_HUMAN --------------------P----CLPHLLSRLC-QDLGPGAFRLAARMYLQKGFPIKE 176

sp|P05155|IC1_HUMAN --------------------TCVHQALKGFTT---------KGVTSVSQIFHSPDLAIRD 234

sp|Q9UK55|ZPI_HUMAN -------------------KPGLLPSLFKGLRETL-SRNLELGLTQGSFAFIHKDFDVKE 176

sp|P01009|A1AT_HUMAN -------------------EAQIHEGFQELLRTLN-QPDSQLQLTTGNGLFLSEGLKLVD 152

sp|P29622|KAIN_HUMAN -------------------ESDVHRGFQHLLHTLN-LPGHGLETRVGSALFLSHNLKFLA 153

sp|P01011|AACT_HUMAN -------------------EAEIHQSFQHLLRTLN-QSSDELQLSMGNAMFVKEQLSLLD 151

sp|P05154|IPSP_HUMAN -------------------EKELHRGFQQLLQELN-QPRDGFQLSLGNALFTDLVVDLQD 143

sp|P05121|PAI1_HUMAN -------------------KGMAP-ALRHLYKELM-GPWNKDEISTTDAIFVQRDLKLVQ 130

sp|P07093|GDN_HUMAN --------------------GVGK-ILKKINKAIV-SKKNKDIVTVANAVFVKNASEIEV 124

sp|Q99574|NEUS_HUMAN -------------------NGEEFSFLKEFSNMVT-AKESQYVMKIANSLFVQNGFHVNE 122

sp|O75830|SPI2_HUMAN -------------------AGEEFFVLKSFFSAIS-EKKQEFTFNLANALYLQEGFTVKE 121

sp|P05546|HEP2_HUMAN ------------------EITTIHNLFRKLTHRLF-RRNFGYTLRSVNDLYIQKQFPILL 234

sp|P01008|ANT3_HUMAN -------------------SDQIHFFFAKLNCRLYRKANKSSKLVSANRLFGDKSLTFNE 188

sp|O75635|SPB7_HUMAN -------------------QSGLQSQLKRVFSDIN-ASHKDYDLSIVNGLFAEKVYGFHK 112

sp|P48595|SPB10_HUMAN ---------KRKMEFNLSNSEEIHSDFQTLISEIL-KPNDDYLLKTANAIYGEKTYAFHN 124

sp|Q96P63|SPB12_HUMAN KSNKQKAG------SLNNESGLVSCYFGQLLSKLD-RIKTDYTLSIANRLYGEQEFPICQ 128

sp|P29508|SPB3_HUMAN -------------TYHVDRSGNVHHQFQKLLTEFN-KSTDAYELKIANKLFGEKTYLFLQ 117

sp|P48594|SPB4_HUMAN -------------TYHVDRSGNVHHQFQKLLTEFN-KSTDAYELKIANKLFGEKTYQFLQ 117

sp|Q9UIV8|SPB13_HUMAN ------------EKEVIENTEAVHQQFQKFLTEIS-KLTNDYELNITNRLFGEKTYLFLQ 118

sp|P05120|PAI2_HUMAN FMQQIQKGSYPDAILQAQAADKIHSSFRSLSSAIN-ASTGNYLLESVNKLFGEKSASFRE 139

sp|P30740|ILEU_HUMAN -------------------VEEVHSRFQSLNADIN-KRGASYILKLANRLYGEKTYNFLP 102

sp|P50453|SPB9_HUMAN -------------------EEDIHRAFQSLLTEVN-KAGTQYLLRTANRLFGEKTCQFLS 102

sp|P35237|SPB6_HUMAN -------------------GGDIHQGFQSLLTEVN-KTGTQYLLRMANRLFGEKSCDFLS 104

sp|P50452|SPB8_HUMAN -------------------DGDIHRGFQSLLSEVN-RTGTQYLLRTANRLFGEKTCDFLP 102

: : .

sp|P08697|A2AP_HUMAN DFLEQSEQLFGAKPVSL--TGKQEDDLANINQWVKEATEGKIQEFL--SGLP-EDTVLLL 231

sp|P05155|IC1_HUMAN TFVNASRTLYSSSPRVL--SNNSDANLELINTWVAKNTNNKISRLL--DSLP-SDTRLVL 289

sp|Q9UK55|ZPI_HUMAN TFFNLSKRYFDTECVPMNFRNAS-QAKRLMNHYINKETRGKIPKLF--DEIN-PETKLIL 232

sp|P01009|A1AT_HUMAN KFLEDVKKLYHSEAFTVNFGDTE-EAKKQINDYVEKGTQGKIVDLV--KELD-RDTVFAL 208

sp|P29622|KAIN_HUMAN KFLNDTMAVYEAKLFHTNFYDTV-GTIQLINDHVKKETRGKIVDLV--SELK-KDVLMVL 209

sp|P01011|AACT_HUMAN RFTEDAKRLYGSEAFATDFQDSA-AAKKLINDYVKNGTRGKITDLI--KDLD-SQTMMVL 207

sp|P05154|IPSP_HUMAN TFVSAMKTLYLADTFPTNFRDSA-GAMKQINDYVAKQTKGKIVDLL--KNLD-SNAVVIM 199

sp|P05121|PAI1_HUMAN GFMPHFFRLFRSTVKQVDFSEVE-RARFIINDWVKTHTKGMISNLLGKGAVD-QLTRLVL 188

sp|P07093|GDN_HUMAN PFVTRNKDVFQCEVRNVNFEDPA-SACDSINAWVKNETRDMIDNLLSPDLIDGVLTRLVL 183

sp|Q99574|NEUS_HUMAN EFLQMMKKYFNAAVNHVDFSQNV-AVANYINKWVENNTNNLVKDLVSPRDFD-AATYLAL 180

sp|O75830|SPI2_HUMAN QYLHGNKEFFQSAIKLVDFQDAK-ACAEMISTWVERKTDGKIKDMFSGEEFG-PLTRLVL 179

sp|P05546|HEP2_HUMAN DFKTKVREYYFAEAQIADFSDPAFISK--TNNHIMKLTKGLIKDAL--ENID-PATQMMI 289

sp|P01008|ANT3_HUMAN TYQDISELVYGAKLQPLDFKENAEQSRAAINKWVSNKTEGRITDVIPSEAIN-ELTVLVL 247

sp|O75635|SPB7_HUMAN DYIECAEKLYDAKVERVDFTNHLEDTRRNINKWVENETHGKIKNVIGEGGIS-SSAVMVL 171

sp|P48595|SPB10_HUMAN KYLEDMKTYFGAEPQPVNFVEASDQIRKDINSWVERQTEGKIQNLLPDDSVD-STTRMIL 183

sp|Q96P63|SPB12_HUMAN EYLDGVIQFYHTTIESVDFQKNPEKSRQEINFWVECQSQGKIKELFSKDAIN-AETVLVL 187

sp|P29508|SPB3_HUMAN EYLDAIKKFYQTSVESVDFANAPEESRKKINSWVESQTNEKIKNLIPEGNIG-SNTTLVL 176

sp|P48594|SPB4_HUMAN EYLDAIKKFYQTSVESTDFANAPEESRKKINSWVESQTNEKIKNLFPDGTIG-NDTTLVL 176

sp|Q9UIV8|SPB13_HUMAN KYLDYVEKYYHASLEPVDFVNAADESRKKINSWVESKTNEKIKDLFPDGSIS-SSTKLVL 177

sp|P05120|PAI2_HUMAN EYIRLCQKYYSSEPQAVDFLECAEEARKKINSWVKTQTKGKIPNLLPEGSVD-GDTRMVL 198

sp|P30740|ILEU_HUMAN EFLVSTQKTYGADLASVDFQHASEDARKTINQWVKGQTEGKIPELLASGMVD-NMTKLVL 161

sp|P50453|SPB9_HUMAN TFKESCLQFYHAELKELSFIRAAEESRKHINTWVSKKTEGKIEELLPGSSID-AETRLVL 161

sp|P35237|SPB6_HUMAN SFRDSCQKFYQAEMEELDFISAVEKSRKHINTWVAEKTEGKIAELLSPGSVD-PLTRLVL 163

sp|P50452|SPB8_HUMAN DFKEYCQKFYQAELEELSFAEDTEECRKHINDWVAEKTEGKISEVLDAGTVD-PLTKLVL 161

: : . : : : . . . . :

sp|P08697|A2AP_HUMAN LNAIHFQGFWRNKFDPSLTQRDSFHLDEQFTVPVEMMQARTYPLRWFLLEQPE------I 285

sp|P05155|IC1_HUMAN LNAIYLSAKWKTTFDPKKTRMEPFHFK-NSVIKVPMMNSKKYPVAHFIDQTLK------A 342

sp|Q9UK55|ZPI_HUMAN VDYILFKGKWLTPFDPVFTEVDTFHLDKYKTIKVPMMYGAG-KFASTFDKNFR------C 285

sp|P01009|A1AT_HUMAN VNYIFFKGKWERPFEVKDTEEEDFHVDQVTTVKVPMMKRLG-MFNIQHCKKLS------S 261

sp|P29622|KAIN_HUMAN VNYIYFKALWEKPFISSRTTPKDFYVDENTTVRVPMMLQDQEHHWYLHDRYLP------C 263

sp|P01011|AACT_HUMAN VNYIFFKAKWEMPFDPQDTHQSRFYLSKKKWVMVPMMSLHHLTIPYFRDEELS------C 261

sp|P05154|IPSP_HUMAN VNYIFFKAKWETSFNHKGTQEQDFYVTSETVVRVPMMSRED-QYHYLLDRNLS------C 252

sp|P05121|PAI1_HUMAN VNALYFNGQWKTPFPDSSTHRRLFHKSDGSTVSVPMMAQTN-KFNYTEFTTPD---GHYY 244

sp|P07093|GDN_HUMAN VNAVYFKGLWKSRFQPENTKKRTFVAADGKSYQVPMLAQLS-VFRCGSTSAPN---DLWY 239

sp|Q99574|NEUS_HUMAN INAVYFKGNWKSQFRPENTRTFSFTKDDESEVQIPMMYQQG-EFYYGEFSDGSNEAGGIY 239

sp|O75830|SPI2_HUMAN VNAIYFKGDWKQKFRKEDTQLINFTKKNGSTVKIPMMKALL-RTKYGYFSESS----LNY 234

sp|P05546|HEP2_HUMAN LNCIYFKGSWVNKFPVEMTHNHNFRLNEREVVKVSMMQTKG-NFLAANDQELD------C 342

sp|P01008|ANT3_HUMAN VNTIYFKGLWKSKFSPENTRKELFYKADGESCSASMMYQEG-KFRYRRVAE-G------T 299

sp|O75635|SPB7_HUMAN VNAVYFKGKWQSAFTKSETINCHFKSPKCSGKAVAMMHQER-KFNLSVIEDPS------M 224

sp|P48595|SPB10_HUMAN VNALYFKGIWEHQFLVQNTTEKPFRINETTSKPVQMMFMKK-KLHIFHIEKPK------A 236

sp|Q96P63|SPB12_HUMAN VNAVYFKAKWETYFDHENTVDAPFCLNANENKSVKMMTQKG-LYRIGFIEEVK------A 240

sp|P29508|SPB3_HUMAN VNAIYFKGQWEKKFNKEDTKEEKFWPNKNTYKSIQMMRQYT-SFHFASLEDVQ------A 229

sp|P48594|SPB4_HUMAN VNAIYFKGQWENKFKKENTKEEKFWPNKNTYKSVQMMRQYN-SFNFALLEDVQ------A 229

sp|Q9UIV8|SPB13_HUMAN VNMVYFKGQWDREFKKENTKEEKFWMNKSTSKSVQMMTQSH-SFSFTFLEDLQ------A 230

sp|P05120|PAI2_HUMAN VNAVYFKGKWKTPFEKKLNGLYPFRVNSAQRTPVQMMYLRE-KLNIGYIEDLK------A 251

sp|P30740|ILEU_HUMAN VNAIYFKGNWKDKFMKEATTNAPFRLNKKDRKTVKMMYQKK-KFAYGYIEDLK------C 214

sp|P50453|SPB9_HUMAN VNAIYFKGKWNEPFDETYTREMPFKINQEEQRPVQMMYQEA-TFKLAHVGEVR------A 214

sp|P35237|SPB6_HUMAN VNAVYFRGNWDEQFDKENTEERLFKVSKNEEKPVQMMFKQS-TFKKTYIGEIF------T 216

sp|P50452|SPB8_HUMAN VNAIYFKGKWNEQFDRKYTRGMLFKTN-EEKKTVQMMFKEA-KFKMGYADEVH------T 213

:: : : . * * . * *:

sp|P08697|A2AP_HUMAN QVAHFPFKNN-MSFVVLVPTHF---EWNVSQVLANLSWDTLHP----PLVWERP----TK 333

sp|P05155|IC1_HUMAN KVGQLQLSHN-LSLVILVPQNL---KHRLEDMEQALSPSVFKAIMEKLEMSK---FQPTL 395

sp|Q9UK55|ZPI_HUMAN HVLKLPYQGN-ATMLVVLMEKM----GDHLALEDYLTTDLVETWL--RNMKTRN----ME 334

sp|P01009|A1AT_HUMAN WVLLMKYLGN-ATAIFFLP-DE----GKLQHLENELTHDIITKFL--ENEDRRS----AS 309

sp|P29622|KAIN_HUMAN SVLRMDYKGD-ATVFFILP-NQ----GKMREIEEVLTPEMLMRWN--NLLRKRNFYKKLE 315

sp|P01011|AACT_HUMAN TVVELKYTGN-ASALFILP-DQ----DKMEEVEAMLLPETLKRWR--DSLEFRE---IGE 310

sp|P05154|IPSP_HUMAN RVVGVPYQGN-ATALFILP-SE----GKMQQVENGLSEKTLRKWL--KMFKKRQ----LE 300

sp|P05121|PAI1_HUMAN DILELPYHGDTLSMFIAAPYEK---EVPLSALTNILSAQLISHWK--GNMTRLP----RL 295

sp|P07093|GDN_HUMAN NFIELPYHGESISMLIALPTES---STPLSAIIPHISTKTIDSWM--SIMVPKR----VQ 290

sp|Q99574|NEUS_HUMAN QVLEIPYEGDEISMMLVLSRQ----EVPLATLEPLVKAQLVEEWA--NSVKKQK----VE 289

sp|O75830|SPI2_HUMAN QVLELSYKGDEFSLIIILPAE----GMDIEEVEKLITAQQILKWL--SEMQEEE----VE 284

sp|P05546|HEP2_HUMAN DILQLEYVGG-ISMLIVVPHKM----SGMKTLEAQLTPRVVERWQ--KSMTNRT----RE 391

sp|P01008|ANT3_HUMAN QVLELPFKGDDITMVLILPKPE----KSLAKVEKELTPEVLQEWL--DELEEMM----LV 349

sp|O75635|SPB7_HUMAN KILELRYNGG-INMYVLLPEN------DLSEIENKLTFQNLMEWTNPRRMTSKY----VE 273

sp|P48595|SPB10_HUMAN VGLQLYYKSRDLSLLILLPEDI----NGLEQLEKAITYEKLNEWTSADMMELYE----VQ 288

sp|Q96P63|SPB12_HUMAN QILEMRYTKGKLSMFVLLPSHSKDNLKGLEELERKITYEKMVAWSSSENMSEES----VV 296

sp|P29508|SPB3_HUMAN KVLEIPYKGKDLSMIVLLPNEI----DGLQKLEEKLTAEKLMEWTSLQNMRETR----VD 281

sp|P48594|SPB4_HUMAN KVLEIPYKGKDLSMIVLLPNEI----DGLQKLEEKLTAEKLMEWTSLQNMRETC----VD 281

sp|Q9UIV8|SPB13_HUMAN KILGIPYKNNDLSMFVLLPNDI----DGLEKIIDKISPEKLVEWTSPGHMEERK----VN 282

sp|P05120|PAI2_HUMAN QILELPYAGD-VSMFLLLPDEIADVSTGLELLESEITYDKLNKWTSKDKMAEDE----VE 306

sp|P30740|ILEU_HUMAN RVLELPYQGEELSMVILLPDDIEDESTGLKKIEEQLTLEKLHEWTKPENLDFIE----VN 270

sp|P50453|SPB9_HUMAN QLLELPYARKELSLLVLLPDDG----VELSTVEKSLTFEKLTAWTKPDCMKSTE----VE 266

sp|P35237|SPB6_HUMAN QILVLPYVGKELNMIIMLPDET----TDLRTVEKELTYEKFVEWTRLDMMDEEE----VE 268

sp|P50452|SPB8_HUMAN QVLELPYVEEELSMVILLPDDN----TDLAVVEKALTYEKFKAWTNSEKLTKSK----VQ 265

. . . : : .

sp|P08697|A2AP_HUMAN VRLPKLYLKHQMDLVATLSQLGLQELFQA--PDLRGISE---QSLVVSGVQHQSTLELSE 388

sp|P05155|IC1_HUMAN LTLPRIKVTTSQDMLSIMEKLEFFDF-SY-DLNLCGLTE--DPDLQVSAMQHQTVLELTE 451

sp|Q9UK55|ZPI_HUMAN VFFPKFKLDQKYEMHELLRQMGIRRIFSP-FADLSELSA-TGRNLQVSRVLQRTVIEVDE 392

sp|P01009|A1AT_HUMAN LHLPKLSITGTYDLKSVLGQLGITKVFSN-GADLSGVTE--EAPLKLSKAVHKAVLTIDE 366

sp|P29622|KAIN_HUMAN LHLPKFSISGSYVLDQILPRLGFTDLFSK-WADLSGITK--QQKLEASKSFHKATLDVDE 372

sp|P01011|AACT_HUMAN LYLPKFSISRDYNLNDILLQLGIEEAFTS-KADLSGITG--ARNLAVSQVVHKAVLDVFE 367

sp|P05154|IPSP_HUMAN LYLPKFSIEGSYQLEKVLPSLGISNVFTS-HADLSGISN--HSNIQVSEMVHKAVVEVDE 357

sp|P05121|PAI1_HUMAN LVLPKFSLETEVDLRKPLENLGMTDMFRQFQADFTSLS--DQEPLHVAQALQKVKIEVNE 353

sp|P07093|GDN_HUMAN VILPKFTAVAQTDLKEPLKVLGITDMFDSSKANFAKITT-GSENLHVSHILQKAKIEVSE 349

sp|Q99574|NEUS_HUMAN VYLPRFTVEQEIDLKDVLKALGITEIFIK-DANLTGLS--DNKEIFLSKAIHKSFLEVNE 346

sp|O75830|SPI2_HUMAN ISLPRFKVEQKVDFKDVLYSLNITEIFSG-GCDLSGIT--DSSEVYVSQVTQKVFFEINE 341

sp|P05546|HEP2_HUMAN VLLPKFKLEKNYNLVESLKLMGIRMLFDK-NGNMAGISD---QRIAIDLFKHQGTITVNE 447

sp|P01008|ANT3_HUMAN VHMPRFRIEDGFSLKEQLQDMGLVDLFSPEKSKLPGIVAEGRDDLYVSDAFHKAFLEVNE 409

sp|O75635|SPB7_HUMAN VFFPQFKIEKNYEMKQYLRALGLKDIFDESKADLSGIAS--GGRLYISRMMHKSYIEVTE 331

sp|P48595|SPB10_HUMAN LHLPKFKLEDSYDLKSTLSSMGMSDAFSQSKADFSGMSS--ARNLFLSNVFHKAFVEINE 346

sp|Q96P63|SPB12_HUMAN LSFPRFTLEDSYDLNSILQDMGITDIFDETRADLTGISP--SPNLYLSKIIHKTFVEVDE 354

sp|P29508|SPB3_HUMAN LHLPRFKVEESYDLKDTLRTMGMVDIFNG-DADLSGMTG--SRGLVLSGVLHKAFVEVTE 338

sp|P48594|SPB4_HUMAN LHLPRFKMEESYDLKDTLRTMGMVNIFNG-DADLSGMTW--SHGLSVSKVLHKAFVEVTE 338

sp|Q9UIV8|SPB13_HUMAN LHLPRFEVEDGYDLEAVLAAMGMGDAFSEHKADYSGMSS--GSGLYAQKFLHSSFVAVTE 340

sp|P05120|PAI2_HUMAN VYIPQFKLEEHYELRSILRSMGMEDAFNKGRANFSGMSE--RNDLFLSEVFHQAMVDVNE 364

sp|P30740|ILEU_HUMAN VSLPRFKLEESYTLNSDLARLGVQDLFNSSKADLSGMSG--ARDIFISKIVHKSFVEVNE 328

sp|P50453|SPB9_HUMAN VLLPKFKLQEDYDMESVLRHLGIVDAFQQGKADLSAMSA--ERDLCLSKFVHKSFVEVNE 324

sp|P35237|SPB6_HUMAN VSLPRFKLEESYDMESVLRNLGMTDAFELGKADFSGMSQ---TDLSLSKVVHKSFVEVNE 325

sp|P50452|SPB8_HUMAN VFLPRLKLEESYDLEPFLRRLGMIDAFDEAKADFSGMST--EKNVPLSKVAHKCFVEVNE 323

: :*:: : : : . . : : : . : *

sp|P08697|A2AP_HUMAN VGVEAAAATSIAMS-RMS-----LSSFSVNRPFLFFIFEDTTGLPLFVGSVRNPNPSAPR 442

sp|P05155|IC1_HUMAN TGVEAAAASAISVA-------RTLLVFEVQQPFLFVLWDQQHKFPVFMGRVYDPRA---- 500

sp|Q9UK55|ZPI_HUMAN RGTEAVAGILSEIT-A----YSMPPVIKVDRPFHFMIYEETSGMLLFLGRVVNPTLL--- 444

sp|P01009|A1AT_HUMAN KGTEAAGAMFLEAI-----PMSIPPEVKFNKPFVFLMIEQNTKSPLFMGKVVNPTQK--- 418

sp|P29622|KAIN_HUMAN AGTEAAAATSFAIK-FFSAQTNR-HILRFNRPFLVVIFSTSTQSVLFLGKVVDPTKP--- 427

sp|P01011|AACT_HUMAN EGTEASAATAVKIT-LLSALVETRTIVRFNRPFLMIIVPTDTQNIFFMSKVTNPKQA--- 423

sp|P05154|IPSP_HUMAN SGTRAAAATGTIFT-FRSARLNSQ-RLVFNRPFLMFIVD---NNILFLGKVNRP------ 406

sp|P05121|PAI1_HUMAN SGTVASSSTAVIVS-ARMA----PEEIIMDRPFLFVVRHNPTGTVLFMGQVMEP------ 402

sp|P07093|GDN_HUMAN DGTKASAATTAILI-ARSS----PPWFIVDRPFLFFIRHNPTGAVLFMGQINKP------ 398

sp|Q99574|NEUS_HUMAN EGSEAAAVSGMIAI-SRMA--VLYPQVIVDHPFFFLIRNRRTGTILFMGRVMHPETMNTS 403

sp|O75830|SPI2_HUMAN DGSEAATSTGIHIP-VIMS--LAQSQFIANHPFLFIMKHNPTESILFMGRVTNPDTQEIK 398

sp|P05546|HEP2_HUMAN EGTQATTVTTV---GFMPL--STQVRFTVDRPFLFLIYEHRTSCLLFMGRVANPSRS--- 499

sp|P01008|ANT3_HUMAN EGSEAAASTAVVIAGRSLN--PNRVTFKANRPFLVFIREVPLNTIIFMGRVANPCVK--- 464

sp|O75635|SPB7_HUMAN EGTEATAATGSNIV-EKQL--PQSTLFRADHPFLFVIRKD--DIILFSGKVSCP------ 380

sp|P48595|SPB10_HUMAN QGTEAAAGSGSEID-IRIR--VPSIEFNANHPFLFFIRHNKTNTILFYGRLCSP------ 397

sp|Q96P63|SPB12_HUMAN NGTQAAAATGAVVS-ERSL--RSWVEFNANHPFLFFIRHNKTQTILFYGRVCSP------ 405

sp|P29508|SPB3_HUMAN EGAEAAAATAVVGFGSSPT--STNEEFHCNHPFLFFIRQNKTNSILFYGRFSSP------ 390

sp|P48594|SPB4_HUMAN EGVEAAAATAVVVVELSSP--STNEEFCCNHPFLFFIRQNKTNSILFYGRFSSP------ 390

sp|Q9UIV8|SPB13_HUMAN EGTEAAAAT-GIGFTVTSA--PGHENVHCNHPFLFFIRHNESNSILFFGRFSSP------ 391

sp|P05120|PAI2_HUMAN EGTEAAAGTGGVMT-GRTG--HGGPQFVADHPFLFLIMHKITNCILFFGRFSSP------ 415

sp|P30740|ILEU_HUMAN EGTEAAAATAGIAT-FCML--MPEENFTADHPFLFFIRHNSSGSILFLGRFSSP------ 379

sp|P50453|SPB9_HUMAN EGTEAAAASSCFVVAECCM--ESGPRFCADHPFLFFIRHNRANSILFCGRFSSP------ 376

sp|P35237|SPB6_HUMAN EGTEAAAATAAIMM-MRCA--RFVPRFCADHPFLFFIQHSKTNGILFCGRFSSP------ 376

sp|P50452|SPB8_HUMAN EGTEAAAATAVVRN-SRCS--RMEPRFCADHPFLFFIRHHKTNCILFCGRFSSP------ 374

* * . ::** ..: .* . . *

sp|P08697|A2AP_HUMAN ELKEQQDSPGNKDFLQSLKGFPRGDKLFGPDLKLVPPMEEDYPQFGSPK 491

sp|P05155|IC1_HUMAN ------------------------------------------------- 500

sp|Q9UK55|ZPI_HUMAN ------------------------------------------------- 444

sp|P01009|A1AT_HUMAN ------------------------------------------------- 418

sp|P29622|KAIN_HUMAN ------------------------------------------------- 427

sp|P01011|AACT_HUMAN ------------------------------------------------- 423

sp|P05154|IPSP_HUMAN ------------------------------------------------- 406

sp|P05121|PAI1_HUMAN ------------------------------------------------- 402

sp|P07093|GDN_HUMAN ------------------------------------------------- 398

sp|Q99574|NEUS_HUMAN GHDFEEL------------------------------------------ 410

sp|O75830|SPI2_HUMAN GRDLDSL------------------------------------------ 405

sp|P05546|HEP2_HUMAN ------------------------------------------------- 499

sp|P01008|ANT3_HUMAN ------------------------------------------------- 464

sp|O75635|SPB7_HUMAN ------------------------------------------------- 380

sp|P48595|SPB10_HUMAN ------------------------------------------------- 397

sp|Q96P63|SPB12_HUMAN ------------------------------------------------- 405

sp|P29508|SPB3_HUMAN ------------------------------------------------- 390

sp|P48594|SPB4_HUMAN ------------------------------------------------- 390

sp|Q9UIV8|SPB13_HUMAN ------------------------------------------------- 391

sp|P05120|PAI2_HUMAN ------------------------------------------------- 415

sp|P30740|ILEU_HUMAN ------------------------------------------------- 379

sp|P50453|SPB9_HUMAN ------------------------------------------------- 376

sp|P35237|SPB6_HUMAN ------------------------------------------------- 376

sp|P50452|SPB8_HUMAN ------------------------------------------------- 374

ClustalW Omega MSA of the reported human non-inhibitory serpins.

**>sp|P01009|A1AT_HUMAN Alpha-1-antitrypsin OS=Homo sapiens GN=SERPINA1 PE=1 SV=3**

>sp|P08185|CBG_HUMAN Corticosteroid-binding globulin OS=Homo sapiens OX=9606 GN=SERPINA6 PE=1 SV=1

>sp|P05543|THBG_HUMAN Thyroxine-binding globulin OS=Homo sapiens OX=9606 GN=SERPINA7 PE=1 SV=2

>sp|P01019|ANGT_HUMAN Angiotensinogen OS=Homo sapiens OX=9606 GN=AGT PE=1 SV=1

>sp|Q86WD7|SPA9_HUMAN Serpin A9 OS=Homo sapiens OX=9606 GN=SERPINA9 PE=1 SV=3

>sp|Q86U17|SPA11_HUMAN Serpin A11 OS=Homo sapiens OX=9606 GN=SERPINA11 PE=2 SV=2

>sp|Q8IW75|SPA12_HUMAN Serpin A12 OS=Homo sapiens OX=9606 GN=SERPINA12 PE=1 SV=1

>sp|Q6UXR4|SPA13_HUMAN Putative serpin A13 OS=Homo sapiens OX=9606 GN=SERPINA13P PE=5 SV=1

>sp|P36952|SPB5_HUMAN Serpin B5 OS=Homo sapiens OX=9606 GN=SERPINB5 PE=1 SV=2

>sp|Q96P15|SPB11_HUMAN Serpin B11 OS=Homo sapiens OX=9606 GN=SERPINB11 PE=2 SV=1

>sp|A8MV23|SERP3_HUMAN Serpin E3 OS=Homo sapiens OX=9606 GN=SERPINE3 PE=2 SV=2

>sp|P36955|PEDF_HUMAN Pigment epithelium-derived factor OS=Homo sapiens OX=9606 GN=SERPINF1 PE=1 SV=4

>sp|P50454|SERPH_HUMAN Serpin H1 OS=Homo sapiens OX=9606 GN=SERPINH1 PE=1 SV=2

CLUSTAL Omega (1.2.4) MSA of human non-inhibitory serpins.

sp|A8MV23|SERP3_HUMAN -----------------------------------------MPPFLITLFLFHSCCLRAN 19

sp|P50454|SERPH_HUMAN ---------------------------------------------MRSLLLLSAFCLLEA 15

sp|P36952|SPB5_HUMAN ------------------------------------------------------------ 0

sp|Q96P15|SPB11_HUMAN ------------------------------------------------------------ 0

sp|P01019|ANGT_HUMAN MRKRAPQSEMAPAGVSLRAT-ILCLLAWAGL-AAGDRVYI--HPFHLVIHNESTCEQLAK 56

sp|P36955|PEDF_HUMAN -----------------------------MQ-------ALVLLLCIGALLGHSSCQNPAS 24

sp|Q6UXR4|SPA13_HUMAN -----------------------------MEASRW-------WLLVTVLMAGAHCVALVD 24

sp|Q8IW75|SPA12_HUMAN -----------------------------MNPTLGLAIFLAVLLTVKGLLKPSFS--PRN 29

sp|Q86U17|SPA11_HUMAN -----------------------------MGP-AWL------WLLGTGILASVHCQPLLA 24

sp|P05543|THBG_HUMAN -----------------------------MSPFLYL------VLLVLGLHATIHCASPEG 25

sp|Q86WD7|SPA9_HUMAN -----------------------------MASYLYG------VLFAVGLCAPIYCVSPAN 25

sp|P01009|A1AT_HUMAN ----------------------------MPSSVSWG------ILLLAGLCCLVPVSLAED 26

sp|P08185|CBG_HUMAN ----------------MPLLLYTCL-LWLPTSGLWT------VQAM-------------D 24

sp|A8MV23|SERP3_HUMAN G-------------------------------------------HLREGMTLLKTEFALH 36

sp|P50454|SERPH_HUMAN ALAAEVKKPAAAAAP---GT-------------------AEKLSPKAATLAERSAGLAFS 53

sp|P36952|SPB5_HUMAN ----------------------------------------------MDALQLANSAFAVD 14

sp|Q96P15|SPB11_HUMAN ----------------------------------------------MGSLSTANVEFCLD 14

sp|P01019|ANGT_HUMAN ANAGKPKDPTFIPAPIQAKTSPVDEKALQDQLVLVAAKLDTEDKLRAAMVGMLANFLGFR 116

sp|P36955|PEDF_HUMAN PPEEGSPDPDST-GALVEEE-------------------DPFFKVPVNKLAAAVSNFGYD 64

sp|Q6UXR4|SPA13_HUMAN QEASDLIHSG-P-----QDS-------------------SPGPALPCHKISVSNIDFAFK 59

sp|Q8IW75|SPA12_HUMAN --YKALSEV----------Q-------------------GWKQRMAAKELARQNMDLGFK 58

sp|Q86U17|SPA11_HUMAN HGDKSLQGPQPP-----RHQ-------------------LSEPAPAYHRITPTITNFALR 60

sp|P05543|THBG_HUMAN KVTA-------C-----HSS---------------------QPNATLYKMSSINADFAFN 52

sp|Q86WD7|SPA9_HUMAN APSA-------Y-----PRP-------------------SSTKSTPASQVYSLNTDFAFR 54

sp|P01009|A1AT_HUMAN PQGDAAQKTD-------TSH-------------------HDQDHPTFNKITPNLAEFAFS 60

sp|P08185|CBG_HUMAN PNAA---YVN-------MSN-------------------H------HRGLASANVDFAFS 49

: :

sp|A8MV23|SERP3_HUMAN LYQSVAA--CRNETNFVISPAGVSLPLEILQFGAEGSTGQQLADALGYTVHDKRVKDFL- 93

sp|P50454|SERPH_HUMAN LYQAMAK--DQAVENILVSPVVVASSLGLVSLGGKATTASQAKAVLSAEQ----LRDEE- 106

sp|P36952|SPB5_HUMAN LFKQLCE--KEPLGNVLFSPICLSTSLSLAQVGAKGDTANEIGQVLHFEN----VKDV-P 67

sp|Q96P15|SPB11_HUMAN VFKELNS--NNIGDNIFFSSLSLLYALSMVLLGARGETAEQLEKVLHFSH----TVDSLK 68

sp|P01019|ANGT_HUMAN IYGMHSELWGVVHGATVLSPTAVFGTLASLYLGALDHTADRLQAILGVPWKDKNCT---- 172

sp|P36955|PEDF_HUMAN LYRVRSS--TSPTTNVLLSPLSVATALSALSLGAEQRTESIIHRALYYDLISS--P---- 116

sp|Q6UXR4|SPA13_HUMAN LYRQLAL--NAPGENILFFPVSISLALAMLSWGAPVASRTQLLEGLGFTLTVVPEE---- 113

sp|Q8IW75|SPA12_HUMAN LLKKLAF--YNPGRNIFLSPLSISTAFSMLCLGAQDSTLDEIKQG--FNFRKMPEK---- 110

sp|Q86U17|SPA11_HUMAN LYKELAA--DAP-GNIFFSPVSISTTLALLSLGAQANTSALILEGLGFNLTETPEA---- 113

sp|P05543|THBG_HUMAN LYRRFTV--ETPDKNIFFSPVSISAALVMLSFGACCSTQTEIVETLGFNLTDTPMV---- 106

sp|Q86WD7|SPA9_HUMAN LYRRLVL--ETPSQNIFFSPVSVSTSLAMLSLGAHSVTKTQILQGLGFNLTHTPES---- 108

sp|P01009|A1AT_HUMAN LYRQLAH--QSNSTNIFFSPVSIATAFAMLSLGTKADTHDEILEGLNFNLTEIPEA---- 114

sp|P08185|CBG_HUMAN LYKHLVA--LSPKKNIFISPVSISMALAMLSLGTCGHTRAQLLQGLGFNLTERSET---- 103

: .. : : * :

sp|A8MV23|SERP3_HUMAN ---------------HAV------YAT------LPTSSQGTEMELACSLFVQVGTPLSPC 126

sp|P50454|SERPH_HUMAN --------------VHAG------LGELLR-SLSNSTARNVTWKLGSRLYGPSSVSFADD 145

sp|P36952|SPB5_HUMAN FGFQ----------------------T-VT-SDVNKLSSFYSLKLIKRLYVDKSLNLSTE 103

sp|Q96P15|SPB11_HUMAN PGFKDSPKCSQAGRIHSE------FGV-EF-SQINQPDSNCTLSIANRLYGTKTMAFHQQ 120

sp|P01019|ANGT_HUMAN ----------SRLDAHKVLSALQAVQGLLVAQGRADSQAQLLLSTVVGVFTAPGLHLKQP 222

sp|P36955|PEDF_HUMAN -------------DIHGT------YKELLD--TVTAPQKNL--KSASRIVFEKKLRIKSS 153

sp|Q6UXR4|SPA13_HUMAN -------------EIQEG------FWDLLI--RLRGQGPRLLLTMDQRRFSGLGAR---- 148

sp|Q8IW75|SPA12_HUMAN -------------DLHEG------FHYIIH--ELTQKTQDLKLSIGNTLFIDQRLQPQRK 149

sp|Q86U17|SPA11_HUMAN -------------DIHQG------FRSLLH--TLALPSPKLELKVGNSLFLDKRLKPRQH 152

sp|P05543|THBG_HUMAN -------------EIQHG------FQHLIC--SLNFPKKELELQIGNALFIGKHLKPLAK 145

sp|Q86WD7|SPA9_HUMAN -------------AIHQG------FQHLVH--SLTVPSKDLTLKMGSALFVKKELQLQAN 147

sp|P01009|A1AT_HUMAN -------------QIHEG------FQELLR--TLNQPDSQLQLTTGNGLFLSEGLKLVDK 153

sp|P08185|CBG_HUMAN -------------EIHQG------FQHLHQ--LFAKSDTSLEMTMGNALFLDGSLELLES 142

sp|A8MV23|SERP3_HUMAN FVEHVSWWAN-SSLEPADLSEPNSTAIQTSEGASRETAGGGPSEGPGGWPWEQVSAAFAQ 185

sp|P50454|SERPH_HUMAN FVRSSKQHYN-CEHSKINFRDKRSA-LQSINEWAAQTTDGKLPEVTKD------VERTDG 197

sp|P36952|SPB5_HUMAN FISSTKRPYA-KELETVDFKDKLEETKGQINNSIKDLTDGHFENILAD----NSVNDQTK 158

sp|Q96P15|SPB11_HUMAN YLSCSEKWYQ-ARLQTVDFEQSTEETRKMINAWVENKTNGKVANLFGK----STIDPSSV 175

sp|P01019|ANGT_HUMAN FVQGLALYTPVVLPRSLDFTELDVA-AEKIDRFMQAVTGWKTGCSLMG------ASVDST 275

sp|P36955|PEDF_HUMAN FVAPLEKSYG-TRPRV-LTGNPRLD-LQEINNWVQAQMKGKLARSTKE------IPDEIS 204

sp|Q6UXR4|SPA13_HUMAN -----------------ANQSLEEA-QKHIDEYTEQQTQGKLGAWEKD------LGSETT 184

sp|Q8IW75|SPA12_HUMAN FLEDAKNFYS-AETILTNFQNLEMA-QKQINDFISQKTHGKINNLIEN------IDPGTV 201

sp|Q86U17|SPA11_HUMAN YLDSIKELYG-AFAFSANFTDSVTT-GRQINDYLRRQTYGQVVDCLPE------FSQDTF 204

sp|P05543|THBG_HUMAN FLNDVKTLYE-TEVFSTDFSNISAA-KQEINSHVEMQTKGKVVGLIQD------LKPNTI 197

sp|Q86WD7|SPA9_HUMAN FLGNVKRLYE-AEVFSTDFSNPSIA-QARINSHVKKKTQGKVVDIIQG------LDLLTA 199

sp|P01009|A1AT_HUMAN FLEDVKKLYH-SEAFTVNFGDTEEA-KKQINDYVEKGTQGKIVDLVKE------LDRDTV 205

sp|P08185|CBG_HUMAN FSADIKHYYE-SEVLAMNFQDWATA-SRQINSYVKNKTQGKIVDLFSG------LDSPAI 194

. :

sp|A8MV23|SERP3_HUMAN LVLVSTMSFQGTWRKRFSSTDTQ-ILPFTCAYGLVLQVPMMHQ-TTEVNYGQFQDTAGHQ 243

sp|P50454|SERPH_HUMAN ALLVNAMFFKPHWDEKFHHKMVD-NRGFMVTRSYTVGVMMMHR-TGLYNYYDDE---KEK 252

sp|P36952|SPB5_HUMAN ILVVNAAYFVGKWMKKFSESETK-ECPFRVNKTDTKPVQMMNM-EATFCMGNID---SIN 213

sp|Q96P15|SPB11_HUMAN MVLVNIIYFKGQRQNKFQVRETV-KSPFQLSEGKNVTVEMMYQ-IGTFKLAFVK---EPQ 230

sp|P01019|ANGT_HUMAN LAFNTYVHFQGKMKG-FSL--LAEPQEFWVDNSTSVSVPMLSG-MGTFQHWSDI---QDN 328

sp|P36955|PEDF_HUMAN ILLLGVAHFKGQWVTKFDSRKTS-LEDFYLDEERTVRVPMMSDPKAVLRYGLDS---DLS 260

sp|Q6UXR4|SPA13_HUMAN AVLVNHMLLRAEWMKPFDSHATS-PKEFFVDEHSAVWVPMMKE-KASHRFLHDR---ELQ 239

sp|Q8IW75|SPA12_HUMAN MLLANYIFFRARWKHEFDPNVTK-EEDFFLEKNSSVKVPMMFR-SGIYQVGYDD---KLS 256

sp|Q86U17|SPA11_HUMAN MVLANYIFFKAKWKHPFSRYQTQKQESFFVDERTSLQVPMMHQ-KEMHRFLYDQ---DLA 260

sp|P05543|THBG_HUMAN MVLVNYIHFKAQWANPFDPSKTEDSSSFLIDKTTTVQVPMMHQ-MEQYYHLVDM---ELN 253

sp|Q86WD7|SPA9_HUMAN MVLVNHIFFKAKWEKPFHPEYTRKNFPFLVGEQVTVHVPMMHQ-KEQFAFGVDT---ELN 255

sp|P01009|A1AT_HUMAN FALVNYIFFKGKWERPFEVKDTE-EEDFHVDQVTTVKVPMMKR-LGMFNIQHCK---KLS 260

sp|P08185|CBG_HUMAN LVLVNYIFFKGTWTQPFDLASTR-EENFYVDETTVVKVPMMLQ-SSTISYLHDS---ELP 249

. : * * * *:

sp|A8MV23|SERP3_HUMAN VGVLELPYLGSAVSLFLVLPRDKD---TPLSHIEPHLTASTIHLWTT--SLRRARMDVFL 298

sp|P50454|SERPH_HUMAN LQIVEMPLAHKLSSLIILMPHHVE----PLERLEKLLTKEQLKIWMG--KMQKKAVAISL 306

sp|P36952|SPB5_HUMAN CKIIELPFQNKHLSMFILLPKDVEDESTGLEKIEKQLNSESLSQWTNPSTMANAKVKLSI 273

sp|Q96P15|SPB11_HUMAN MQVLELPYVNNKLSMIILLPVGIA----NLKQIEKQLNSGTFHEWTSSSNMMEREVEVHL 286

sp|P01019|ANGT_HUMAN FSVTQVPFTESACLLLIQPHYA-S----DLDKVEGLTFQQNSLNWMK--KLSPRTIHLTM 381

sp|P36955|PEDF_HUMAN CKIAQLPLTGSMSIIFFLPLKVTQ----NLTLIEESLTSEFIHDIDR--ELKTVQAVLTV 314

sp|Q6UXR4|SPA13_HUMAN CSVLRMDHAGNTTTFFIFPNRG------KMRHLEDALLPETLIKWDS--LLRTRELDFHF 291

sp|Q8IW75|SPA12_HUMAN CTILEIPYQKNITAIFILPDEG------KLKHLEKGLQVDTFSRWKT--LLSRRVVDVSV 308

sp|Q86U17|SPA11_HUMAN CTVLQIEYRGNALALLVLPDPG------KMKQVEAALQPQTLRKWGQ--LLLPSLLDLHL 312

sp|P05543|THBG_HUMAN CTVLQMDYSKNALALFVLPKEG------QMESVEAAMSSKTLKKWNR--LLQKGWVDLFV 305

sp|Q86WD7|SPA9_HUMAN CFVLQMDYKGDAVAFFVLPSKG------KMRQLEQALSARTLRKWSH--SLQKRWIEVFI 307

sp|P01009|A1AT_HUMAN SWVLLMKYLGNATAIFFLPDEG------KLQHLENELTHDIITKFLE--NEDRRSASLHL 312

sp|P08185|CBG_HUMAN CQLVQMNYVGNGTVFFILPDKG------KMNTVIAALSRDTINRWSA--GLTSSQVDLYI 301

: : . ::. : : . .

sp|A8MV23|SERP3_HUMAN PRFRIQNQFNLKSILNSWGVTDLFDPLKANLKGISGQDGFYVSEAIHKAKIEVLEEGTKA 358

sp|P50454|SERPH_HUMAN PKGVVEVTHDLQKHLAGLGLTEAIDKNKADLSRMSGKKDLYLASVFHATAFELDTDGNPF 366

sp|P36952|SPB5_HUMAN PKFKVEKMIDPKACLENLGLKHIFSEDTSDFSGMSETKGVALSNVIHKVCLEITEDGGDS 333

sp|Q96P15|SPB11_HUMAN PRFKLEIKYELNSLLKPLGVTDLFNQVKADLSGMSPTKGLYLSKAIHKSYLDVSEEGTEA 346

sp|P01019|ANGT_HUMAN PQLVLQGSYDLQDLLAQAELPAILHT-ELNLQKLSND-RIRVGEVLNSIFFELEADEREP 439

sp|P36955|PEDF_HUMAN PKLKLSYEGEVTKSLQEMKLQSLFD--SPDFSKITGK-PIKLTQVEHRAGFEWNEDGAGT 371

sp|Q6UXR4|SPA13_HUMAN PKFSISRTCRLEMLLP-------------------------------------------- 307

sp|Q8IW75|SPA12_HUMAN PRLHMTGTFDLKKTLSYIGVSKIFEE-HGDLTKIAPHRSLKVGEAVHKAELKMDERGTEG 367

sp|Q86U17|SPA11_HUMAN PRFSISGTYNLEDILPQIGLTNILNL-EADFSGVTGQLNKTISKVSHKAMVDMSEKGTEA 371

sp|P05543|THBG_HUMAN PKFSISATYDLGATLLKMGIQHAYSE-NADFSGLTEDNGLKLSNAAHKAVLHIGEKGTEA 364

sp|Q86WD7|SPA9_HUMAN PRFSISASYNLETILPKMGIQNVFDK-NADFSGIAKRDSLQVSKATHKAVLDVSEEGTEA 366

sp|P01009|A1AT_HUMAN PKLSITGTYDLKSVLGQLGITKVFSN-GADLSGVTEEAPLKLSKAVHKAVLTIDEKGTEA 371

sp|P08185|CBG_HUMAN PKVTISGVYDLGDVLEEMGIADLFTN-QANFSRITQDAQLKSSKVVHKAVLQLNEEGVDT 360

*: : *

sp|A8MV23|SERP3_HUMAN SGATALLLLK--R--SRIPIFKADRPFIYFLREPNTGITVFFDRIQIIYQCLSSNKGSFV 414

sp|P50454|SERPH_HUMAN DQ---DIYGREEL--RSPKLFYADHPFIFLVRDTQSGSLLFIGRLVRP-------KGDKM 414

sp|P36952|SPB5_HUMAN IEVPGARI----L--QHKDELNADHPFIYIIRHNKTRNIIFFGKFCSP------------ 375

sp|Q96P15|SPB11_HUMAN AAATGDSIAVKSL--PMRAQFKANHPFLFFIRHTHTNTILFCGKLASP------------ 392

sp|P01019|ANGT_HUMAN TESTQ------QLNKPEVLEVTLNRPFLFAVYDQSATALHFLGRVANPLSTA-------- 485

sp|P36955|PEDF_HUMAN TPSPGLQPAHLTF----PLDYHLNQPFIFVLRDTDTGALLFIGKILDPRGP--------- 418

sp|Q6UXR4|SPA13_HUMAN ------------------------------------------------------------ 307

sp|Q8IW75|SPA12_HUMAN AAGTGAQTLPMET----PLVVKIDKPYLLLIYSEKIPSVLFLGKIVNPIGK--------- 414

sp|Q86U17|SPA11_HUMAN GAASGLLSQPPSLNTMSDPHAHFNRPFLLLLWEVTTQSLLFLGKVVNPVAG--------- 422

sp|P05543|THBG_HUMAN AAVPEVELSDQPENTFLHPIIQIDRSFMLLILERSTRSILFLGKVVNPTEA--------- 415

sp|Q86WD7|SPA9_HUMAN TAATTTKFIVRSKDGPSYFTVSFNRTFLMMITNKATDGILFLGKVENPTKS--------- 417

sp|P01009|A1AT_HUMAN AGAMFLEAIPMSI----PPEVKFNKPFVFLMIEQNTKSPLFMGKVVNPTQK--------- 418

sp|P08185|CBG_HUMAN AGSTGVTLNLTSK----PIILRFNQPFIIMIFDHFTWSSLFLARVMNPV----------- 405

sp|A8MV23|SERP3_HUMAN HYPLKNKHSF 424

sp|P50454|SERPH_HUMAN RDEL------ 418

sp|P36952|SPB5_HUMAN ---------- 375

sp|Q96P15|SPB11_HUMAN ---------- 392

sp|P01019|ANGT_HUMAN ---------- 485

sp|P36955|PEDF_HUMAN ---------- 418

sp|Q6UXR4|SPA13_HUMAN ---------- 307

sp|Q8IW75|SPA12_HUMAN ---------- 414

sp|Q86U17|SPA11_HUMAN ---------- 422

sp|P05543|THBG_HUMAN ---------- 415

sp|Q86WD7|SPA9_HUMAN ---------- 417

sp|P01009|A1AT_HUMAN ---------- 418

sp|P08185|CBG_HUMAN ---------- 405
